# DNA barcoding of marine fish species from Rongcheng Bay, China

**DOI:** 10.7717/peerj.5013

**Published:** 2018-06-25

**Authors:** Lijuan Wang, Zhihao Wu, Mengxia Liu, Wei Liu, Wenxi Zhao, Hongjun Liu, Feng You

**Affiliations:** 1 CAS Key Laboratory of Experimental Marine Biology, Institute of Oceanology, Chinese Academy of Sciences, Qingdao, Shandong, China; 2 Laboratory for Marine Biology and Biotechnology, Qingdao National Laboratory for Marine Science and Technology, Qingdao, Shandong, China; 3 Marine Biology Institute of Shandong Province, Qingdao, Shandong, China; 4 Center for Ocean Mega-Science, Chinese Academy of Sciences, Qingdao, Shandong, China

**Keywords:** Cytochrome c oxidase subunit I (COI), DNA barcoding, Fish identification, Rongcheng Bay

## Abstract

Rongcheng Bay is a coastal bay of the Northern Yellow Sea, China. To investigate and monitor the fish resources in Rongcheng Bay, 187 specimens from 41 different species belonging to 28 families in nine orders were DNA-barcoded using the mitochondrial cytochrome c oxidase subunit I gene (*COI*). Most of the fish species could be discriminated using this COI sequence with the exception of *Cynoglossus joyneri* and *Cynoglossus lighti.* The average GC% content of the 41 fish species was 47.3%. The average Kimura 2-parameter genetic distances within the species, genera, families, and orders were 0.21%, 5.28%, 21.30%, and 23.63%, respectively. Our results confirmed that the use of combined morphological and DNA barcoding identification methods facilitated fish species identification in Rongcheng Bay, and also established a reliable DNA barcode reference library for these fish. DNA barcodes will contribute to future efforts to achieve better monitoring, conservation, and management of fisheries in this area.

## Introduction

There are approximately 30,000 fish species worldwide, constituting slightly more than one-half of the recognized living vertebrates (Nelson, Grande & Wilson, 2016). The Chinese fish fauna also has high species richness, with more than 3,200 marine species described from Chinese coastal waters (Liu, 2011). Owing to dramatic expansion in fish production, particularly from aquaculture, fish availability in China has grown steadily, with apparent per capita fish consumption increasing by an average of 6% annually in the period 1990–2010 (FAO, 2014). It is imperative that the ichthyofauna of China be well studied for effective conservation and resource management. Accurate and unambiguous identification of fish will assist in managing fisheries for long-term sustainability, and will improve ecosystem research and conservation.

However, this presents a resource challenge. Traditional determination methods, such as morphological identification, require a considerable amount of taxonomic expertise. The DNA-based barcoding method has been proven to be a valuable molecular tool for species identification and it is accessible to non-specialists (Hebert, Ratnasingham & Dewaard, 2003; Frézal & Leblois, 2008; Leray & Knowlton, 2015). A number of international campaigns are focused on DNA barcoding whole biota, including fish; FISH-BOL (http://www.fishbol.org), for example, is now well established and aims at DNA barcoding all the fishes of the world (Ward, Hanner & Hebert, 2009). DNA barcoding is useful not only for the identification of whole fish but also for the identification of larvae, eggs, fillets, fins, and other fragments of the body that are difficult to identify based on morphology (Trivedi et al., 2016). The mitochondrial *COI* gene has been accepted as the standard region for DNA barcoding (Hebert, Ratnasingham & Dewaard, 2003; Hajibabaei et al., 2007a; Hajibabaei et al., 2007b) and it is extremely effective at discriminating fish species (Ward et al., 2005; Hubert et al., 2008; Valdez-Moreno et al., 2009). Approximately 98% of reported marine fish species can be distinguished by COI barcoding, and this approach has been used to catalogue and record fish in many geographic regions (Aquilino et al., 2011; Asgharian et al., 2011; Cawthorn, Steinman & Witthuhn, 2011; Lakra et al., 2011; Becker et al., 2015). However, there have been only a few DNA barcoding studies of the marine fish resources of China, and most of them have focused on the South China Sea (Zhang, 2011; Wang et al., 2012; Zhang & Hanner, 2012).

Rongcheng Bay is a coastal bay of the Northern Yellow Sea in Shandong Province, China, and it is one of the most important coastal regions for fisheries in China. The fisheries in this area are a major source of food and have helped coastal communities to maintain their livelihoods and community structure. Although it is an important spawning and nursery area for many marine species, scant information is available on fish species richness, barring two reports by Yu et al. (2013) and Wang et al. (2016). These investigators studied species composition and seasonal variation in community structure and reported the length–weight relationships of 13 fish species in Rongcheng Bay, but they did not undertake systematic DNA barcoding or other molecular data collection. Our study aimed to complement their dataset using DNA barcoding in order to better investigate and monitor fish resources and implement conservation efforts.

## Materials and methods

### Ethics statement

The study was conducted in accordance with the guidelines and regulations established by China Government Principles for the Utilization and Care of Animals Used in Testing, Research, and Training. All other applicable international, national, and institutional guidelines for the care and use of animals were followed by the authors. The animal work and animal protocols were approved by Institute of Oceanology, Chinese Academy of Science. Permits and approval of field studies have been obtained by the authors from the Institute of Oceanology, Chinese Academy of Sciences (201305043 and 200805069).

### Fish samples

**Figure 1:**
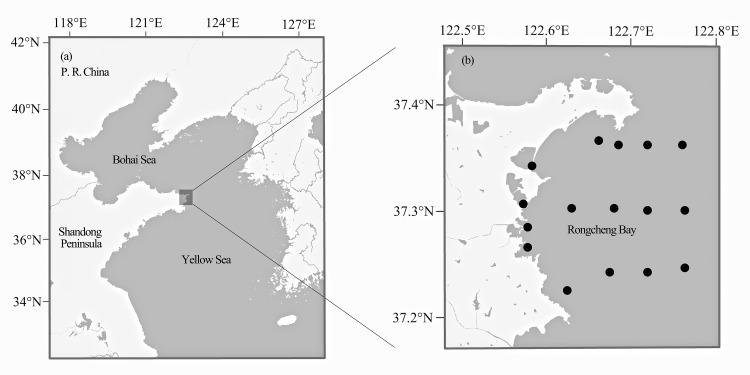
Map of Rongcheng Bay showing sampling sites. Locations of Rongcheng Bay (A) and sampling sites (B).

Fish samples were collected from Rongcheng Bay, Northern Yellow Sea (37°12′–37°24′N, 122°33′–122°48′E) (Fig. 1), using bottom trawl nets and stake nets from spring 2011 to winter 2014. One to 10 individual specimens were collected for each fish species. All captured fish samples were immediately stored on ice and transported to the laboratory for identification. Whole-specimen vouchers were deposited in the Key Laboratory of Experimental Marine Biology, Institute of Oceanology, Chinese Academy of Sciences. All specimens were identified to the species level based on morphology by consulting a standard fish reference (Cheng & Zheng, 1987) and FishBase (http://www.fishbase.org). Muscle tissue was excised from each specimen and stored at −20 °C until use. To avoid cross-contamination between fish samples, the inner part muscle of each individual was collected with tweezers and scissors, which were sterilized with alcohol and alcohol lamp. Every sample was collected by using a separate tool set.

### DNA extraction and PCR amplification

Total genomic DNA was extracted from muscle tissue by salt extraction (Aljanabi & Martinez, 1997). Concentration and purity of the extracted DNA were measured using a NanoDrop 2000 spectrophotometer (Thermo Scientific, Wilmington, DE, USA). Approximately 650 bp was amplified from the 5′ region of the mitochondrial COI gene using different combinations of the following primers designed by Ward et al. (2005).

FishF1 5′-TCAACCAACCACAAAGACATTGGCAC-3′

FishF2 5′-TCGACTAATCATAAAGATATCGGCAC-3′

FishR1 5′-TAGACTTCTGGGTGGCCAAAGAATCA-3′

FishR2 5′-ACTTCAGGGTGACCGAAGAATCAGAA-3′

The 50-µl PCR mixtures included 5 µl of 10 × PCR buffer, 2.5 µl of MgCl_2_ (50 mM), 1 µl of dNTP (0.05 mM), 2 µl of each primer (0.01 mM), 0.125µl of each dNTP (0.05 mM), 1.25 U of Taq polymerase, 2.0 µl of DNA template, and ultrapure water to 50 µl. The amplification procedure consisted of an initial denaturation step at 95 °C for 5 min, followed by 35 cycles at 95 °C for 30 s, 52 °C 30 s, and 72 °C for 60 s, then a final extension at 72 °C for 10 min. PCR products were visualized on 1.2% agarose gels and purified by the EZNA TM gel extraction kit (Omega Bio-Tek, Norcross, GA, USA). The purified PCR products were sent to Shanghai Sunny Biotechnology Co. Ltd., China, for bidirectional sequencing using an ABI 3730 capillary sequencer (Applied Biosystems, Foster City, CA, USA).

### DNA sequence analysis

The DNA sequences were assembled, aligned, and annotated using BioEdit software (Hall, 1999). The obtained sequences were double compared to sequences of fishes in both GenBank and BOLD databases, and the similarity index with available sequences of same fish species from databases were over 98% for all sequences. Distance-and character-based DNA barcoding methods for species discrimination were used in this study. Pairwise genetic distances were calculated using the Kimura 2-parameter (K_2_P) distance model (Kimura, 1980). Neighbor-joining (NJ) trees of K_2_P distances with 1,000 bootstrap replications (Saitou & Nei, 1987) were generated to provide a graphic representation of the patterning of divergence between species. The K_2_P distance and the neighbor-joining (NJ) tree were calculated and generated using MEGA version 5 (Tamura et al., 2011). BLOG 2.0 (Weitschek et al., 2013) was used for character-based identification of fish species represented by more than two individuals. The COI sequences of *Cynoglossus joyneri* and *Cynoglossus lighti* downloaded from GenBank (accession numbers: GU479053.1, JQ738602.1, JQ738613.1, DQ116752.1, JQ738430.1, JQ738456.1, JQ738468.1, KF979127.1, HQ711865.1) together with the sequences generated from this study were used to construct the NJ tree with 1000 bootstrap replications by using MEGA.

## Results

A total of 187 COI sequences (GenBank accession numbers: KU236800–KU236892 and KY275270–KY275363) were obtained from 41 fish species belonging to 28 families and nine orders (Table 1). Sequence lengths ranged from 605 to 655 bp, with an average of 635 bp. No stop codons, insertions, or deletions were observed in any of the amplified sequences. The overall average nucleotide composition of the sequences was 23.5% A, 29.2% T, 18.7% G, and 28.5% C, with an A + T bias (Table 2). The percent GC content of five fish species, namely *Chelidonichthys kumu*, *Neosalanx anderssoni*, *Lateolabrax japonicus*, *Saurida elongata*, and *Zoarces elongatus,* was found to be more than 50% (Table 3). Among these, *N. anderssoni* (Osmeriformes) showed the highest GC content (53.4%). Among the other 36 species, *Pterogobius zacalles* (Perciformes) showed the lowest GC content (41.4%).

**Table 1:** Fish species collected from Rongcheng Bay.

Order	Family	Species	*N*	GenBank Accession no.
Scorpaeniformes	Sebastidae	*Sebastes schlegelii*	10	KU236800, KU236801, KU236802, KU236803, KU236804, KU236805, KU236806, KU236807, KU236808, KY275270
		*Sebastes koreanus*	10	KY275271, KY275272, KY275273, KY275274, KY275275, KY275276, KY275277, KY275278, KY275279, KY275280
	Liparidae	*Liparis tanakae*	3	KU236809, KU236810, KY275281
		*Liparis chefuensis*	1	KY275282
	Hemitripteridae	*Hemitripterus villosus*	3	KU236811, KU236812, KU236813
	Platycephalidae	*Platycephalus indicus*	1	KU236814
	Triglidae	*Chelidonichthys kumu*	5	KU236815, KU236816, KU236817, KU236818, KU236819
	Hexagrammidae	*Hexagrammos otakii*	10	KU236820, KU236821, KU236822, KU236823, KY275283, KY275284, KY275285, KY275286, KY275287, KY275288
		*Hexagrammos agrammus*	10	KY275289, KY275290, KY275291, KY275292, KY275293, KY275294, KY275295, KY275296, KY275297, KY275298
Pleuronectiformes	Pleuronectidae	*Kareius bicoloratus*	3	KU236824, KU236825, KU236826
		*Pseudopleuronectes yokohamae*	3	KY275299, KY275300, KY275301
	Paralichthyidae	*Paralichthys olivaceus*	7	KU236827, KU236828, KU236829, KU236830, KU236831, KU236832, KU236833
	Cynoglossidae	*Cynoglossus joyneri*	5	KU236834, KU236835, KY275302, KY275303, KY275304
		*Cynoglossus lighti*	3	KU236836, KU236837, KU236838
Perciformes	Ammodytidae	*Ammodytes personatus*	1	KU236839
	Pholidae	*Pholis fangi*	6	KU236840, KU236841, KU236842, KU236843, KU236844, KU236845
		*Pholis crassispina*	10	KY275305, KY275306, KY275307, KY275308, KY275309, KY275310, KY275311, KY275312, KY275313, KY275314
	Stichaeidae	*Chirolophis japonicus*	10	KU236846, KU236847, KU236848, KY275315, KY275316, KY275317, KY275318, KY275319, KY275320, KY275321
		*Ernogrammus hexagrammus*	1	KY275322
	Zoarcidae	*Zoarces elongatus*	10	KU236849, KU236850, KU236851, KU236852, KU236853, KU236854, KY275323, KY275324, KY275325, KY275326
	Gobiidae	*Chaeturichthys stigmatias*	8	KU236855, KU236856, KY275327, KY275328, KY275329, KY275330, KY275331, KY275332
		*Synechogobius ommaturus*	10	KY275333, KY275334, KY275335, KY275336, KY275337, KY275338, KY275339, KY275340, KY275341, KY275342
		*Acanthogobius flavimanus*	6	KY275343, KY275344, KY275345, KY275346, KY275347, KY275348
		*Pterogobius zacalles*	5	KY275349, KY275350, KY275351, KY275352, KY275353
	Sciaenidae	*Larimichthys polyactis*	1	KU236857
		*Pennahia argentata*	5	KU236858, KU236859, KU236860, KU236861, KU236862
	Sillaginidae	*Sillago japonica*	1	KU236863
	Apogonidae	*Apogon lineatus*	3	KU236864, KU236865, KU236866
	Sparidae	*Acanthopagrus schlegelii*	2	KU236867, KY275354
	Scombridae	*Scomber japonicus*	1	KU236868
		*Saurida elongata*	1	KU236869
	Percichthyidae	*Lateolabrax japonicus*	3	KY275355, KY275356, KY275357
Anguilliformes	Congridae	*Conger myriaster*	10	KU236870, KU236871, KU236872, KU236873, KU236874, KY275358, KY275359, KY275360, KY275361, KY275362
Osmeriformes	Salangidae	*Neosalanx anderssoni*	1	KU236875
Lophiiformes	Lophiidae	*Lophius litulon*	2	KU236876, KU236877
Tetraodontiformes	Monacanthidae	*Thamnaconus modestus*	1	KU236878
	Tetraodontidae	*Takifugu niphobles*	1	KY275363
Clupeiformes	Clupeidae	*Konosirus punctatus*	2	KU236879, KU236880
	Engraulidae	*Engraulis japonicus*	1	KU236881
		*Thryssa kammalensis*	5	KU236882, KU236883, KU236884, KU236885, KU236886
Rajiformes	Rajidae	*Okamejei kenojei*	6	KU236887, KU236888, KU236889, KU236890, KU236891, KU236892

**Notes.**

*N* number of specimens

**Table 2:** Mean percentage base composition and GC content of the first, second, and third codon positions from 187 COI sequences of 41 fishes collected from Rongcheng Bay.

	Mean	Minimum	Maximum	Standard error
A%	23.5	20.6	26.8	0.1098
T%	29.2	25.7	34.3	0.1475
C%	28.5	24.3	34.4	0.1475
G%	18.7	16.2	21.0	0.0694
GC%	47.3	41.4	53.4	0.1912
GC% codon 1st position	56.6	54.1	58.9	0.3626
GC% codon 2nd position	43.0	41.5	44.7	0.0401
GC% codon 3rd position	42.2	24.8	60.3	0.5717

**Figure 2:**
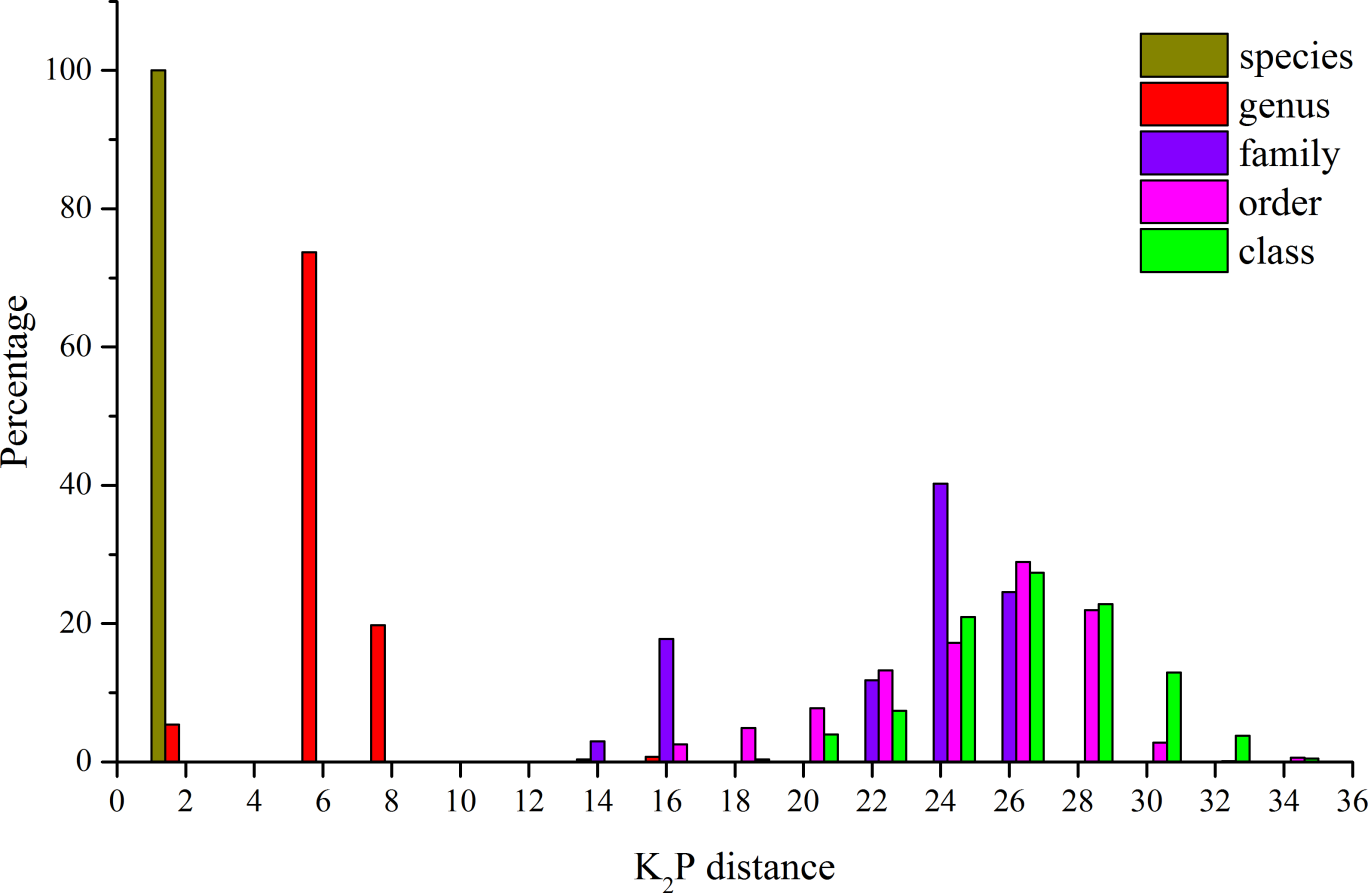
Distribution of *K*_2_*P* distances (percent) for mitochondrial COI at different taxonomic levels for the 41 species analyzed.

The K_2_P genetic distances within each taxonomic level are summarized in Table 4. The average genetic distance using K_2_P analysis of individuals within species was 0.21% (Table 4). Within genera, families, and orders, the distances were 5.28%, 21.30%, and 23.63%, respectively (Table 4). An increase in genetic variation at increasing taxonomic levels was observed, but the rate of increase declined in the higher taxonomic categories (Fig. 2). In this study, some species were represented by a single specimen, but for the majority of the species (29), multiple specimens were analyzed (Table 1) and character-based analyses successfully identified those species. The BLOG method of species diagnosis is based only on nucleotides of specific sites in particular taxa, and these diagnostic sites are referred to as nucleotide diagnostics (ND). Thirteen species were identified with two ND, while the other species were diagnosed using an ND of three to four nucleotide positions in combination (Table 5).

**Table 3:** Mean percent base composition ± SD of COI sequences of fishes collected from Rongcheng Bay.

Species	T	C	A	G	GC
*Sebastes schlegelii*	28.96 ± 0.05	28.52 ± 0.05	23.90 ± 0.05	18.61 ± 0.05	47.13 ± 0.08
*Sebastes koreanus*	28.99 ± 0.09	28.99 ± 0.05	23.86 ± 0.10	18.17 ± 0.08	47.15 ± 0.09
*Liparis tanakae*	29.25 ± 0.00	29.25 ± 0.00	23.48 ± 0.09	18.02 ± 0.09	47.27 ± 0.09
*Liparis chefuensis*	31.88 ± 0.00	26.86 ± 0.00	23.95 ± 0.00	17.31 ± 0.00	44.17 ± 0.00
*Hemitripterus villosus*	28.25 ± 0.00	30.16 ± 0.00	22.38 ± 0.00	19.21 ± 0.00	49.37 ± 0.00
*Platycephalus indicus*	27.45 ± 0.00	30.52 ± 0.00	22.85 ± 0.00	19.17 ± 0.00	49.69 ± 0.00
*Chelidonichthys kumu*	26.73 ± 0.00	32.50 ± 0.10	21.30 ± 0.20	19.50 ± 0.20	52.00 ± 0.20
*Hexagrammos otakii*	29.59 ± 0.07	28.96 ± 0.07	21.88 ± 0.11	19.57 ± 0.11	48.53 ± 0.08
*Hexagrammos agrammus*	29.66 ± 0.00	29.06 ± 0.00	22.06 ± 0.07	19.23 ± 0.07	48.29 ± 0.07
*Kareius bicoloratus*	26.42 ± 0.00	29.25 ± 0.00	24.00 ± 0.09	20.34 ± 0.09	49.58 ± 0.09
*Pseudopleuronectes yokohamae*	26.56 ± 0.00	30.08 ±0.00	24.68 ± 0.09	18.68 ± 0.09	48.75 ± 0.09
*Paralichthys olivaceus*	28.34 ± 0.08	29.70 ± 0.06	24.03 ± 0.09	17.93 ± 0.06	47.63 ± 0.12
*Cynoglossus joyneri*	30.46 ± 0.14	26.58 ± 0.07	26.23 ± 0.10	16.73 ± 0.11	43.31 ± 0.15
*Cynoglossus lighti*	29.89 ± 0.00	26.55 ± 0.00	26.34 ± 0.09	17.22 ± 0.09	43.77 ± 0.09
*Ammodytes personatus*	30.20 ± 0.00	28.79 ± 0.00	22.22 ± 0.00	18.78 ± 0.00	47.57 ± 0.00
*Pholis fangi*	29.74 ± 0.07	28.73 ± 0.12	21.70 ± 0.07	19.83 ± 0.07	48.56 ± 0.10
*Pholis crassispina*	30.71 ± 0.13	27.58 ± 0.13	21.93 ± 0.08	19.79 ± 0.15	47.37 ± 0.15
*Chirolophis japonicus*	29.83 ± 0.08	27.92 ± 0.08	22.91 ± 0.11	19.34 ± 0.11	47.26 ± 0.00
*Ernogrammus hexagrammus*	29.67 ± 0.00	28.26 ± 0.00	24.02 ± 0.00	18.05 ± 0.00	46.31 ± 0.00
*Zoarces elongatus*	26.01 ± 0.19	32.81 ± 0.16	21.71 ± 0.17	19.47 ± 0.19	52.28 ± 0.23
*Chaeturichthys stigmatias*	31.10 ± 0.23	27.75 ± 0.23	23.76 ± 0.00	17.38 ± 0.00	45.14 ± 0.23
*Synechogobius ommaturus *	30.25 ± 0.09	27.28 ± 0.05	23.58 ± 0.06	18.89 ± 0.10	46.17 ± 0.11
*Acanthogobius flavimanus*	28.08 ± 0.00	28.71 ± 0.00	24.13 ± 0.00	19.09 ± 0.00	47.97 ± 0.00
*Pterogobius zacalles*	34.30 ± 0.00	25.24±0.00	24.27 ± 0.00	16.18 ± 0.00	41.42 ± 0.00
*Larimichthys polyactis*	26.11 ± 0.00	30.38 ± 0.00	24.53 ± 0.00	18.99 ± 0.00	49.37 ± 0.00
*Pennahia argentata*	28.30 ± 0.20	29.70 ±0.30	23.60 ± 0.10	18.40 ± 0.10	48.00 ± 0.20
*Sillago japonica*	30.00 ± 0.00	27.80 ± 0.00	22.60 ± 0.00	19.60 ± 0.00	47.40 ± 0.00
*Apogon lineatus*	29.62 ± 0.00	27.87 ± 0.00	24.36 ± 0.00	18.15 ± 0.00	46.02 ± 0.00
*Acanthopagrus schlegelii*	31.00 ± 0.13	26.63±0.32	24.49 ± 0.16	17.88 ± 0.34	44.51 ± 0.02
*Scomber japonicus*	27.40 ± 0.00	30.20 ± 0.00	22.60 ± 0.00	19.80 ± 0.00	50.00 ± 0.00
*Saurida elongata*	28.97 ± 0.00	30.53 ± 0.00	20.56 ± 0.00	19.94 ± 0.00	50.47 ± 0.00
*Lateolabrax japonicus*	26.71 ± 0.09	31.94 ± 0.09	22.60 ± 0.28	18.75 ± 0.28	50.69 ± 0.37
*Conger myriaster*	31.39 ± 0.00	24.35 ± 0.00	26.68 ± 0.06	17.58 ± 0.08	41.93 ± 0.06
*Neosalanx anderssoni*	25.80 ± 0.00	34.35 ± 0.00	20.76 ± 0.00	19.08 ± 0.00	53.44 ± 0.00
*Lophius litulon*	27.04 ± 0.00	29.40 ± 0.00	24.37 ± 0.00	19.18 ± 0.00	48.58 ± 0.00
*Thamnaconus modestus*	29.13 ± 0.00	28.80 ± 0.00	23.14 ± 0.00	18.93 ± 0.00	47.73 ± 0.00
*Takifugu niphobles*	28.06 ± 0.00	29.39 ± 0.00	24.52 ± 0.00	18.02 ± 0.00	47.42 ± 0.00
*Konosirus punctatus*	28.03 ± 0.00	28.82 ± 0.00	22.13 ± 0.00	21.02 ± 0.00	49.84 ± 0.00
*Engraulis japonicus*	25.96 ± 0.22	32.82 ± 0.19	21.73 ± 0.22	19.49 ± 0.17	52.32 ± 0.28
*Thryssa kammalensis*	29.72 ± 0.22	25.63 ± 0.11	26.26 ± 0.00	18.40 ± 0.00	44.03 ± 0.11
*Okamejei kenojei*	27.12 ± 0.06	29.74 ± 0.08	24.87 ± 0.16	18.28 ± 0.19	48.02 ± 0.22

**Table 4:** Summary of genetic divergences (K_2_P percent) within various taxonomic levels.

Comparisons within	Distance			Standard error
	Mean	Minimum	Maximum	
Species	0.21	0.00	1.91	0.0120
Genera	5.28	0.00	14.05	0.0965
Families	21.30	7.45	25.72	0.2325
Orders	23.63	14.41	33.05	0.0506
Classes	25.24	17.54	33.88	0.0265

**Figure 3:**
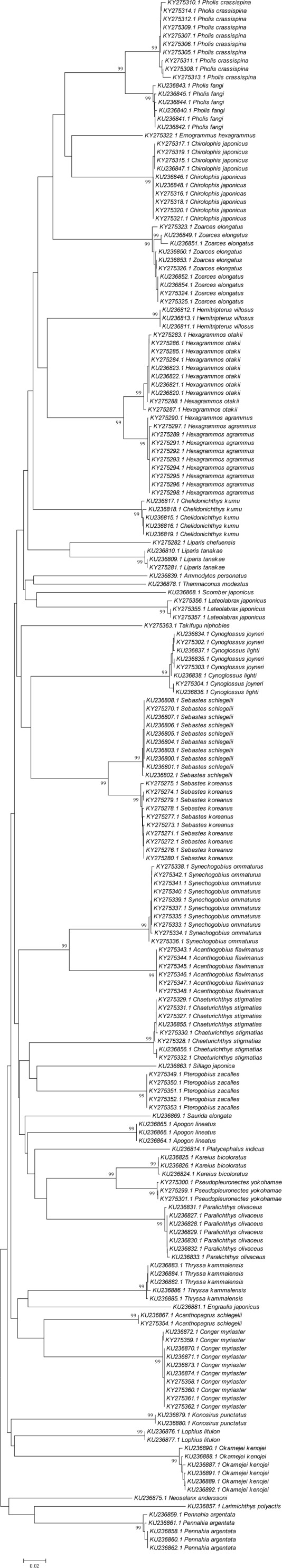
Neighbor-joining (NJ) tree of 187 COI sequences from 41 fish species, using K_2_P distances. This phylogenetic tree was constructed using the NJ method, and bootstrap analysis with 1,000 replicates was used to assess the strength of the nodes.

**Figure 4:**
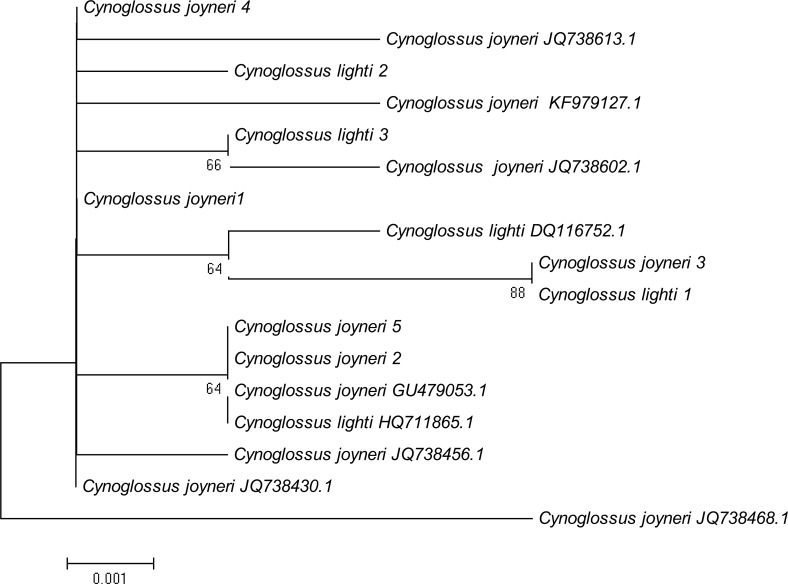
NJ tree using K_2_P distances of COI sequences of *Cynoglossus joyneri* and *Cynoglossus lighti.* This phylogenetic tree was constructed using the NJ method, and bootstrap analysis with 1,000 replicates was used to assess the strength of the nodes.

A phylogenetic NJ tree was generated based on all individuals’ DNA barcode sequences (Fig. 3). Most individuals from each species belonged to single monophyletic clusters, except for *Cynoglossus joyneri* and *Cynoglossus lighti*. Instead of forming two separate branches, these individuals clustered under a single node. In order to validate our sequences, additional COI sequences of these species were downloaded from GenBank and analyzed together with the sequences generated from our study. The resulting NJ tree (Fig. 4) showed that the *C. joyneri and C. lighti* voucher sequences were also non-monophyletic. The K_2_P distances within *C. joyneri* ranged from 0% to 1.23%*,* with an average value of 0.48%, and the values within *C. lighti* ranged from 0.35% to 0.70%*,* with an average value of 0.53%. The minimum, maximum, and average K_2_P distances between *C. joyneri and C. lighti* were 0%, 1.23%, and 0.48%, respectively.

**Table 5:** Nucleotide diagnostics (NDs) for 29 fish species collected from Rongcheng Bay.

Species	NDs
*Sebastes schlegelii*	C-250+A-286+A-493
*Sebastes koreanus*	T-206+C-T-231+A-474
*Liparis tanakae*	T-251+A-287+T-494
*Hemitripterus villosus*	C-252+A-288+T-495
*Chelidonichthys kumu*	C-327+G-501
*Hexagrammos otakii*	C-251+T-320+A-494
*Hexagrammos agrammus*	T-251+T-287+T-320+A-494
*Kareius bicoloratus*	C-291+A-327+A-501
*Pseudopleuronectes yokohamae*	C-274+T-310+A-343
*Paralichthys olivaceus*	A-242+C-311
*Cynoglossus joyneri*	C-276+T-309+T-483
*Cynoglossus lighti*	T-320+C-494
*Pholis fangi*	A-284+C-491
*Pholis crassispina*	G-310+C-517
*Chirolophis japonicus*	T-259+G-295+T-502
*Zoarces elongatus*	C-241+G-277+T-484
*Chaeturichthys stigmatias*	T-249+C-285+C-318
*Synechogobius ommaturus *	T-296+G-329
*Acanthogobius flavimanus*	A-253+G-322
*Pterogobius zacalles*	C-240+T-256+C-309
*Pennahia argentata*	G-286+G-493
*Apogon lineatus*	C-286+T-319+A-493
*Acanthopagrus schlegelii*	T-179+A-259
*Lateolabrax japonicus*	T-163+C-279+A-486
*Conger myriaster*	A-274+A-310
*Lophius litulon*	T-250+C-286+G-319
*Konosirus punctatus*	T-286+A-493
*Thryssa kammalensis*	A-294+G-501
*Okamejei kenojei*	A-321+T-495

## Discussion

Traditional morphological species identification requires experienced taxonomists, and the phenotypic plasticity of taxa may lead to misidentifications. The DNA barcoding method has been proven to be an effective tool for species identification, particularly with specimens that are damaged, incomplete, or consisting of several morphologically distinct stages. Nevertheless, DNA barcoding also has limitations. In some cases, related species may present identical sequences making DNA barcodes useless for species discrimination. Therefore, DNA barcoding can serve as a complementary tool for the identification of species, but it cannot replace morphological taxonomic analyses (Pečnikar & Buzan, 2014). In this study, DNA barcode analysis based on the COI gene was able to identify most fishes in Rongcheng Bay, and the identification results were in agreement with that of morphological identification. DNA barcoding was successfully used to identify the marine ichthyofauna assignment in other geographic regions (for example, the Mediterranean Sea, Landi et al., 2014). In this study, a reliable DNA barcode reference library for the fish in Rongcheng Bay was established, which could be used to assign fish species by screening sequences against it in the future. This would contribute to achieving better monitoring, conservation, and management of fisheries in this area.

The variation of GC content affects different codon positions. Generally, the second codon position shows the lowest variation and the third codon position shows the largest range of variation (Min & Hickey, 2007), which is consistent with our results (Table 2). These differences between the codon positions may reflect the degree of selective constraint, therefore GC content could provide a significant insight into the nature of selective pressures impacting nucleotide usage (Clare et al., 2008). GC content was also proved to be correlated with some bio-functions, such as DNA helix (Vinogradov, 2003) and gene expression (Quax et al., 2015). The mitochondrial GC shifts in nucleotide composition can be explained by mutational biases, natural selection (Mooers & Holmes, 2000) and other factors, such as environmental temperature (Bernardi & Bernardi, 1986) and amino acid content (Foster & Hickey, 1999). The sequences obtained in this study, together with other datasets, may facilitate further investigation of these hypotheses.

Although it is highly controversial (Srivathsan & Meier, 2012), the distance-based technique remains as the standard approach in DNA barcoding (Reid et al., 2011). In this study, the K2P model was used in this study to ensure consistency and comparability with other barcoding studies. The intraspecific genetic divergence (about 0.2%) was much smaller than the interspecific genetic divergence (about 10%) in Rongcheng Bay (with the exception of *C. joyneri* and *C. lighti*). These results indicate that using COI gene sequences as DNA barcodes to discriminate fish species in Rongcheng Bay is feasible. Increasing average genetic distance values were obtained at increasing taxonomic levels in this study. The average genetic distances between individuals within species, genera, families, and orders were 0.21%, 5.28%, 21.3%, and 23.63%, respectively, consistent with the patterns observed in other fish barcoding studies. For example, the K_2_P values of Australian fish within species, families, and orders were 0.39%, 15.46%, and 22.18%, respectively (Ward et al., 2005); the values of Indian marine fishes were 0.30% within species, 9.91% within families, and 16.00% within orders (Lakra et al., 2011); and the values of fishes from South China Sea within species, families, and orders were 0.32%, 20.20%, and 24.66%, respectively (Zhang, 2011).

Most of the 41 fishes in this study have been recorded previously in the Yellow Sea (Liu & Ning, 2011; Yu et al., 2013; Wang et al., 2016). According to Liu & Ning (2011), five of these fish are warm water species, 20 are warm-temperate species, and 16 are cold-temperate species (Table 6). No cold-water species were present. However, cold-temperate fish accounted for a high proportion of the species were observed (39% of the total) in this study. This may be a result of the Yellow Sea cold water mass, wherein a 70-80 meter depression in the central part of the Yellow Sea (He, Wang & Lei, 1959), holds cold water throughout the year and provides an important habitat for cold-temperate fish. Based on the literature and fish databases (Froese & Pauly, 1998; Liu & Ning, 2011; Shao, 2011), the habitat preferences of these 41 fish species can be grouped into five categories. In the most dominant category, 31 were associated with continental shelf demersal habitats. The other species sampled included two continental shelf reef-associated species, four continental shelf pelagic-neritic species, three oceanic pelagic species, and one oceanic bathydemersal species. The species composition in Rongcheng Bay is similar to that in other bays along the coast of the Yellow Sea, such as Laizhou Bay and Jiaozhou Bay (Xu et al., 2013; Sun et al., 2014). However, these regions do not yet have an analogous DNA barcoding database for comparison with our study.

**Table 6:** Summary of economic values, suitable temperature, habitat type and seasonal occurrence of 41 fish species sampled in Rongcheng Bay.

Species	Economic value	Suitable temperature	Habitat type	Occurrence Seasons			
				Spring	Summer	Autumn	Winter
*Sebastes schlegelii*	H	CT	CD	+	+	+	+
*Sebastes koreanus*	N	CT	CD	+	+	+	
*Liparis tanakae*	L	CT	CD	+	+	+	+
*Liparis chefuensis*	L	CT	CD		+		
*Hemitripterus villosus*	N	CT	CD			+	
*Platycephalus indicus*	H	WW	CRA	+		+	+
*Chelidonichthys kumu*	N	WT	CD		+	+	
*Hexagrammos otakii*	H	CT	CD	+	+	+	+
*Hexagrammos agrammus*	N	CT	CD	+	+	+	+
*Kareius bicoloratus*	H	CT	CD	+	+		+
*Pseudopleuronectes yokohamae*	H	CT	CD				
*Paralichthys olivaceus*	H	WT	CD	+	+		
*Cynoglossus joyneri*	N	WT	CD	+	+	+	+
*Cynoglossus lighti*	N	WT	CD	+	+		
*Ammodytes personatus*	N	CT	CD	+	+	+	+
*Pholis fangi*	L	CT	CD	+	+		+
*Pholis crassispina*	L	CT	CD	+	+	+	+
*Chirolophis japonicus*	N	CT	CD	+			
*Ernogrammus hexagrammus*	L	CT	CD	+			
*Zoarces elongatus*	N	CT	CD	+		+	+
*Chaeturichthys stigmatias*	L	WT	CD	+	+	+	
*Synechogobius ommaturus*	N	WT	CD	+	+	+	+
*Acanthogobius flavimanus*	L	CT	CD	+	+	+	+
*Pterogobius zacalles*	L	WT	CD	+	+	+	
*Larimichthys polyactis*	H	WT	CBD	+			
*Pennahia argentata*	H	WW	CBD			+	
*Sillago japonica*	N	WW	CD	+			
*Apogon lineatus*	L	WT	CD		+		
*Acanthopagrus schlegelii*	H	WT	CD			+	
*Scomber japonicus*	H	WW	CPN		+		
*Saurida elongata*	H	WT	CD			+	
*Lateolabrax japonicus*	H	WT	CRA	+	+		
*Conger myriaster*	H	WT	CD	+	+	+	
*Neosalanx anderssoni*	N	WT	CD	+			
*Lophius litulon*	N	WT	OMP	+	+		
*Thamnaconus modestus*	N	WT	CRA			+	
*Takifugu niphobles*	N	WT	CD			+	
*Konosirus punctatus*	N	WT	CPN			+	
*Engraulis japonicus*	N	WT	CPN		+		
*Thryssa kammalensis*	N	WW	CPN	+			
*Okamejei kenojei*	H	WT	CD	+	+	+	+

**Notes.**

H high economic value species N normal economic value species L low economic value species WT warm-temperate species WW warm water species CT cold-temperate species CW cold water species CD continental shelf demersal fish CBD continental shelf benthopelagic fish CPN continental shelf pelagic-neritic fish CRA continental shelf reef-associated fish OMP oceanic bathydemersal fish

Of the 41 species sampled in this study, there are 14 with high commercial value (Table 6). Some traditionally and economically important fishes, such as *Larimichthys polyactis* and *Scomber japonicus,* were caught seldom or only once during our investigation. Meanwhile, some less-valuable species have become dominant, implying that fish species assemblages and population sizes in this area have changed in keeping with other reports (Jin & Tang, 1996; Jin, 2003; Xu & Jin, 2005). Therefore, the economically valuable fish species should be better protected, and DNA barcoding of the ichthyofauna of this bay has contributed additional information toward this goal.

Finally, our study has brought to light an interesting relationship between two species belonging to the subfamily Cynoglossinae, *C. joyneri* and *C. lighti*, which exhibited genetic distances that were very small (from 0% to 1.23%). The resulting NJ tree showed that these two fishes did not form distinct monophyletic clusters and were not clearly separated from each other. Voucher sequences from GenBank were consistent with these findings. Moreover, the morphological taxonomy in the subfamily Cynoglossinae is contentious, and several disputes about species delimitation have arisen (Matsubara, 1955; Ochiai, 1963; Li & Wang, 1995). It is difficult to morphologically distinguish *C. joyneri* from *C. lighti*. The appearance of these two fishes is very similar, and the main differences are the number of lateral-line scales and the head to tail length ratio (Li & Wang, 1995). On a molecular level, our study did not find sufficient interspecific genetic differentiation to regard these species as truly separate. These results support the findings of Liu et al. (2010), who analyzed genetic differentiation among individuals of these two species using partial 16S rRNA and *Cyt b* mitochondrial gene sequences, and found that genetic differentiation in these gene sequences was small. On this basis, they hypothesized that *C. joyneri* and *C. lighti* are probably the same species. Owing to the limited number of samples and genes surveyed in our study we hesitate to say conclusively that they are conspecific, however. Further investigation combining morphological data and the divergence of multiple molecular markers, including nuclear genes, will be required to confirm the taxonomic status of *C. joyneri* and *C. lighti*.

## Conclusions

Rongcheng Bay is a coastal bay of the Northern Yellow Sea, China, and its ichthyofauna has its own unique features. Therefore, it is very important to identify the fish species from this area. DNA barcoding is a molecular method that uses a short standardized DNA sequence of the mitochondrial COI as a species identification tool. In this study, 187 specimens from 41 different species belonging to 28 families in nine orders were DNA-barcoded. The average genetic distance using K_2_P analysis of individuals within species, genera, families, and orders were 0.21%, 5.28%, 21.30%, and 23.63%, respectively. There are no overlaps of pairwise genetic variations between conspecific and interspecific comparisons apart from the species *Cynoglossus joyneri* and *Cynoglossus lighti* in genus *Cynoglossus.* Our results confirm that DNA barcoding can be used as an effective tool for fast and accurate fish identification in Rongcheng Bay.

##  Supplemental Information

10.7717/peerj.5013/supp-1 File S1 The COI sequences obtained in this study

10.7717/peerj.5013/supp-2 File S2 Alignment of COI sequences obtained from this study

10.7717/peerj.5013/supp-3 File S3 The COI sequences of *Cynoglossus joyneri* and *Cynoglossus lighti* used to construct NJ tree

## Additional Information and Declarations

### Field Study Permissions

The following information was supplied relating to field study approvals (i.e., approving body and any reference numbers): The research permit was issued from the Institute of Oceanology, Chinese Academy of Sciences (Permit number: 201305043 and 200805069).

### DNA Deposition

The following information was supplied regarding the deposition of DNA sequences: The Cytochrome Oxidase I sequences obtained in this study are accessible via GenBank accession numbers KU236800–KU236892 and KY275270–KY275363.

### Data Availability

The following information was supplied regarding data availability: The raw data are provided in the Supplemental Files.
