# Supplementary material for: DNA barcoding of marine fish species from Rongcheng Bay, China
Source: PeerJ. 2018 Jun 25;6:e5013. doi: 10.7717/peerj.5013 (PMC6022726; doi:10.7717/peerj.5013)
Supplement: File S1 [file peerj-06-5013-s001.docx]

>KU236800.1 *Sebastes schlegelii*

AGCCGGTATAGTAGGCACAGCCCTCAGCCTACTTATTCGAGCAGAACTAAGCCAACCGGGCGCTCTCCTTGGAGACGACCAAATTTATAATGTAATCGTTACAGCACATGCCTTCGTAATGATTTTCTTTATAGTAATGCCAATTATGATTGGAGGTTTTGGGAACTGGTTAATTCCCCTAATGATCGGAGCCCCAGATATAGCATTTCCTCGTATGAATAATATAAGTTTCTGACTTCTACCCCCTTCCTTCCTACTACTACTTGCCTCCTCTGGTGTAGAAGCAGGTGCCGGAACCGGATGAACAGTGTACCCGCCCCTGGCTGGTAATTTAGCCCACGCAGGAGCATCAGTCGACCTGACAATCTTTTCACTTCACCTGGCAGGTATTTCCTCAATCCTTGGGGCTATCAATTTTATTACCACAATTATTAATATGAAACCTCCAGCTATCTCTCAGTACCAGACACCTCTATTTGTGTGAGCCGTCCTAATTACCGCTGTTCTTCTCCTTCTCTCTTTACCAGTCCTCGCTGCCGGCATCACAATGCTCCTTACCGACCGAAACCTTAATACCACCTTCTTTGACCCGGCCGGGGGAGGGGACCCAATCCTTTACCAGCACTTA

>KU236801.1 *Sebastes schlegelii*

AGCCGGTATAGTAGGCACAGCCCTCAGCCTACTTATTCGAGCAGAACTAAGCCAACCGGGCGCTCTCCTTGGAGACGACCAAATTTATAATGTAATCGTTACAGCACATGCCTTCGTAATGATTTTCTTTATAGTAATGCCAATTATGATTGGAGGTTTTGGGAACTGGTTAATTCCCCTAATGATCGGAGCCCCAGATATAGCATTTCCTCGTATAAATAATATAAGTTTCTGACTTCTACCCCCTTCCTTCCTACTACTACTTGCCTCCTCTGGTGTAGAAGCAGGTGCCGGAACCGGATGAACAGTGTACCCGCCCCTGGCTGGTAATTTAGCCCACGCAGGAGCATCAGTCGACCTGACAATCTTTTCACTTCACCTGGCAGGTATTTCCTCAATCCTTGGGGCTATCAATTTTATTACCACAATTATTAATATGAAACCTCCAGCTATCTCTCAGTACCAGACACCTCTATTTGTGTGAGCCGTCCTAATTACCGCTGTTCTTCTCCTTCTCTCTTTACCAGTCCTCGCTGCCGGCATCACAATGCTCCTTACCGACCGAAACCTTAATACCACCTTCTTTGACCCGGCCGGGGGAGGGGACCCAATCCTTTACCAGCACTTA

>KU236802.1 *Sebastes schlegelii*

AGCCGGTATAGTAGGCACAGCCCTCAGCCTACTTATTCGAGCAGAACTAAGCCAACCGGGCGCTCTCCTTGGAGACGACCAAATTTATAATGTAATCGTTACAGCACATGCCTTCGTAATGATTTTCTTTATAGTAATGCCAATTATGATTGGAGGTTTTGGGAACTGGTTAATTCCCCTAATGATCGGAGCCCCAGATATAGCATTTCCTCGTATGAATAATATAAGTTTCTGACTTCTACCCCCCTCCTTCCTACTACTACTTGCCTCCTCTGGTGTAGAAGCAGGTGCCGGAACCGGATGAACAGTGTACCCGCCCCTGGCTGGTAATTTAGCCCACGCAGGAGCATCAGTCGACCTGACAATCTTTTCACTTCACCTGGCAGGTATTTCCTCAATCCTTGGGGCTATCAATTTTATTACCACAATTATTAATATGAAACCTCCAGCTATCTCTCAGTACCAGACACCTCTATTTGTGTGAGCCGTCCTAATTACCGCTGTTCTTCTCCTTCTCTCTTTACCAGTCCTCGCTGCCGGCATCACAATGCTCCTTACCGACCGAAACCTTAATACCACCTTCTTTGACCCGGCCGGGGGAGGGGACCCAATCCTTTACCAGCACTTA

>KU236803.1 *Sebastes schlegelii*

AGCCGGTATAGTAGGCACAGCCCTCAGCCTACTTATTCGAGCAGAACTAAGCCAACCGGGCGCTCTCCTTGGAGACGACCAAATTTATAATGTAATCGTTACAGCACATGCCTTCGTAATGATTTTCTTTATAGTAATGCCAATTATGATTGGAGGTTTTGGGAACTGGTTAATTCCCCTAATGATCGGAGCCCCAGATATAGCATTTCCTCGTATGAATAATATAAGTTTCTGACTTCTACCCCCTTCCTTCCTACTACTACTTGCCTCCTCTGGTGTAGAAGCAGGTGCCGGAACCGGATGAACAGTGTACCCGCCCCTGGCTGGTAATTTAGCCCACGCAGGAGCATCAGTCGACCTGACAATCTTTTCACTTCACCTGGCAGGTATTTCCTCAATCCTTGGGGCTATCAATTTTATTACCACAATTATTAATATGAAACCTCCAGCTATCTCTCAGTACCAGACACCTCTATTTGTGTGAGCCGTCCTAATTACCGCTGTTCTTCTCCTTCTCTCTTTACCAGTCCTCGCTGCCGGCATCACAATGCTCCTTACCGACCGAAACCTTAATACCACCTTCTTTGACCCGGCCGGGGGAGGGGACCCAATCCTTTACCAGCACTTA

>KU236804.1 *Sebastes schlegelii*

AGCCGGTATAGTAGGCACAGCCCTCAGCCTACTTATTCGAGCAGAACTAAGCCAACCGGGCGCTCTCCTTGGAGACGACCAAATTTATAATGTAATCGTTACAGCACATGCCTTCGTAATGATTTTCTTTATAGTAATGCCAATTATGATTGGAGGTTTTGGGAACTGGTTAATTCCCCTAATGATCGGAGCCCCAGATATAGCATTTCCTCGTATGAATAATATAAGTTTCTGACTTCTACCCCCTTCCTTCCTACTACTACTTGCCTCCTCTGGTGTAGAAGCAGGTGCCGGAACCGGATGAACAGTGTACCCGCCCCTGGCTGGTAATTTAGCCCACGCAGGAGCATCAGTCGACCTGACAATCTTTTCACTTCACCTGGCAGGTATTTCCTCAATCCTTGGGGCTATCAATTTTATTACCACAATTATTAATATGAAACCTCCAGCTATCTCTCAGTACCAGACACCTCTATTTGTGTGAGCCGTCCTAATTACCGCTGTTCTTCTCCTTCTCTCTTTACCAGTCCTCGCTGCCGGCATCACAATGCTCCTTACCGACCGAAACCTTAATACCACCTTCTTTGACCCGGCCGGGGGAGGGGACCCAATCCTTTACCAGCACTTA

>KU236805.1 *Sebastes schlegelii*

AGCCGGTATAGTAGGCACAGCCCTCAGCCTACTTATTCGAGCAGAACTAAGCCAACCGGGCGCTCTCCTTGGAGACGACCAAATTTATAATGTAATCGTTACAGCACATGCCTTCGTAATGATTTTCTTTATAGTAATGCCAATTATGATTGGAGGTTTTGGGAACTGGTTAATTCCCCTAATGATCGGAGCCCCAGATATAGCATTTCCTCGTATGAATAATATAAGTTTCTGACTTCTACCCCCTTCCTTCCTACTACTACTTGCCTCCTCTGGTGTAGAAGCAGGTGCCGGAACCGGATGAACAGTGTACCCGCCCCTGGCTGGTAATTTAGCCCACGCAGGAGCATCAGTCGACCTGACAATCTTTTCACTTCACCTGGCAGGTATTTCCTCAATCCTTGGGGCTATCAATTTTATTACCACAATTATTAATATGAAACCTCCAGCTATCTCTCAGTACCAGACACCTCTATTTGTGTGAGCCGTCCTAATTACCGCTGTTCTTCTCCTTCTCTCTTTACCAGTCCTCGCTGCCGGCATCACAATGCTCCTTACCGACCGAAACCTTAATACCACCTTCTTTGACCCGGCCGGGGGAGGGGACCCAATCCTTTACCAGCACTTA

>KU236806.1 *Sebastes schlegelii*

AGCCGGTATAGTAGGCACAGCCCTCAGCCTACTTATTCGAGCAGAACTAAGCCAACCGGGCGCTCTCCTTGGAGACGACCAAATTTATAATGTAATCGTTACAGCACATGCCTTCGTAATGATTTTCTTTATAGTAATGCCAATTATGATTGGAGGTTTTGGGAACTGGTTAATTCCCCTAATGATCGGAGCCCCAGATATAGCATTTCCTCGTATGAATAATATAAGTTTCTGACTTCTACCCCCTTCCTTCCTACTACTACTTGCCTCCTCTGGTGTAGAAGCAGGTGCCGGAACCGGATGAACAGTGTACCCGCCCCTGGCTGGTAATTTAGCCCACGCAGGAGCATCAGTCGACCTGACAATCTTTTCACTTCACCTGGCAGGTATTTCCTCAATCCTTGGGGCTATCAATTTTATTACCACAATTATTAATATGAAACCTCCAGCTATCTCTCAGTACCAGACACCTCTATTTGTGTGAGCCGTCCTAATTACCGCTGTTCTTCTCCTTCTCTCTTTACCAGTCCTCGCTGCCGGCATCACAATGCTCCTTACCGACCGAAACCTTAATACCACCTTCTTTGACCCGGCCGGGGGAGGGGACCCAATCCTTTACCAGCACTTA

>KU236807.1 *Sebastes schlegelii*

AGCCGGTATAGTAGGCACAGCCCTCAGCCTACTTATTCGAGCAGAACTAAGCCAACCGGGCGCTCTCCTTGGAGACGACCAAATTTATAATGTAATCGTTACAGCACATGCCTTCGTAATGATTTTCTTTATAGTAATGCCAATTATGATTGGAGGTTTTGGGAACTGGTTAATTCCCCTAATGATCGGAGCCCCAGATATAGCATTTCCTCGTATGAATAATATAAGTTTCTGACTTCTACCCCCTTCCTTCCTACTACTACTTGCCTCCTCTGGTGTAGAAGCAGGTGCCGGAACCGGATGAACAGTGTACCCGCCCCTGGCTGGTAATTTAGCCCACGCAGGAGCATCAGTCGACCTGACAATCTTTTCACTTCACCTGGCAGGTATTTCCTCAATCCTTGGGGCTATCAATTTTATTACCACAATTATTAATATGAAACCTCCAGCTATCTCTCAGTACCAGACACCTCTATTTGTGTGAGCCGTCCTAATTACCGCTGTTCTTCTCCTTCTCTCTTTACCAGTCCTCGCTGCCGGCATCACAATGCTCCTTACCGACCGAAACCTTAATACCACCTTCTTTGACCCGGCCGGGGGAGGGGACCCAATCCTTTACCAGCACTTA

>KU236808.1 *Sebastes schlegelii*

AGCCGGTATAGTAGGCACAGCCCTCAGCCTACTTATTCGAGCAGAACTAAGCCAACCGGGCGCTCTCCTTGGAGACGACCAAATTTATAATGTAATCGTTACAGCACATGCCTTCGTAATGATTTTCTTTATAGTAATGCCAATTATGATTGGAGGTTTTGGGAACTGGTTAATTCCCCTAATGATCGGAGCCCCAGATATAGCATTTCCTCGTATGAATAATATAAGTTTCTGACTTCTACCCCCTTCCTTCCTACTACTACTTGCCTCCTCTGGTGTAGAAGCAGGTGCCGGAACCGGATGAACAGTGTACCCGCCCCTGGCTGGTAATTTAGCCCACGCAGGAGCATCAGTCGACCTGACAATCTTTTCACTTCACCTGGCAGGTATTTCCTCAATCCTTGGGGCTATCAATTTTATTACCACAATTATTAATATGAAACCTCCAGCTATCTCTCAGTACCAGACACCTCTATTTGTGTGAGCCGTCCTAATTACCGCTGTTCTTCTCCTTCTCTCTTTACCAGTCCTCGCTGCCGGCATCACAATGCTCCTTACCGACCGAAACCTTAATACCACCTTCTTTGACCCGGCCGGGGGAGGGGACCCAATCCTTTACCAGCACTTA

>KY275270.1 *Sebastes schlegelii*

AGCCGGTATAGTAGGCACAGCCCTCAGCCTACTTATTCGAGCAGAACTAAGCCAACCGGGCGCTCTCCTTGGAGACGACCAAATTTATAATGTAATCGTTACAGCACATGCCTTCGTAATGATTTTCTTTATAGTAATGCCAATTATGATTGGAGGTTTTGGGAACTGGTTAATTCCCCTAATGATCGGAGCCCCAGATATAGCATTTCCTCGTATGAATAATATAAGTTTCTGACTTCTACCCCCTTCCTTCCTACTACTACTTGCCTCCTCTGGTGTAGAAGCAGGTGCCGGAACCGGATGAACAGTGTACCCGCCCCTGGCTGGTAATTTAGCCCACGCAGGAGCATCAGTCGACCTGACAATCTTTTCACTTCACCTGGCAGGTATTTCCTCAATCCTTGGGGCTATCAATTTTATTACCACAATTATTAATATGAAACCTCCAGCTATCTCTCAGTACCAGACACCTCTATTTGTGTGAGCCGTCCTAATTACCGCTGTTCTTCTCCTTCTCTCTTTACCAGTCCTCGCTGCCGGCATCACAATGCTCCTTACCGACCGAAACCTTAATACCACCTTCTTTGACCCGGCCGGGGGAGGGGACCCAATCCTTTACCAGCACTTA

>KY275271.1 *Sebastes koreanus*

CCCTCAGCCTACTCATTCGAGCAGAACTAAGCCAACCGGGCGCTCTCCTTGGAGACGACCAAATTTACAATGTAATCGTTACAGCACATGCCTTCGTAATGATTTTCTTTATAGTAATGCCAATTATAATTGGAGGTTTTGGAAACTGATTAATTCCCCTAATGATTGGAGCCCCAGATATAGCATTTCCTCGTATAAATAATATAAGTTTCTGACTTCTTCCCCCTTCTTTCCTGCTACTACTTGCCTCCTCTGGAGTAGAGGCAGGTGCCGGAACCGGGTGAACAGTGTACCCGCCCCTGGCTGGTAATTTAGCCCACGCAGGAGCATCAGTCGACCTGACAATCTTTTCACTCCACCTGGCAGGTATTTCCTCAATCCTTGGGGCAATCAATTTTATTACCACAATTATTAATATGAAACCTCCGGCCATCTCCCAATACCAGACACCCCTGTTTGTGTGGGCCGTCTTAATTACCGCTGTCCTTCTCCTTCTCTCTCTACCAGTCCTCGCTGCTGGCATCACAATACTCCTTACGGACCGGAACCTTAATACCACCTTCTTTGACCCGGCAGGAGGGGGAGATCCAATCCTTTACCAGCACTTATTC

>KY275272.1 *Sebastes koreanus*

CCCTCAGCCTACTCATTCGAGCAGAACTAAGCCAACCGGGCGCTCTCCTTGGAGACGACCAAATTTACAATGTAATCGTTACAGCACATGCCTTCGTAATGATTTTCTTTATAGTAATGCCAATTATAATTGGAGGTTTTGGAAACTGATTAATTCCCCTAATGATTGGAGCCCCAGATATAGCATTTCCTCGTATAAATAATATAAGTTTCTGACTTCTTCCCCCTTCTTTCCTGCTACTACTTGCCTCCTCTGGAGTAGAGGCAGGTGCCGGAACCGGGTGAACAGTGTACCCGCCCCTGGCTGGTAATTTAGCCCACGCAGGAGCATCAGTCGACCTGACAATCTTTTCACTCCACCTGGCAGGTATTTCCTCAATCCTTGGGGCAATCAATTTTATTACCACAATTATTAATATGAAACCTCCGGCCATCTCCCAATACCAGACACCCCTGTTTGTGTGGGCCGTCTTAATTACCGCTGTCCTTCTCCTTCTCTCTCTACCAGTCCTCGCTGCTGGCATCACAATACTCCTTACGGACCGGAACCTTAATACCACCTTCTTTGACCCGGCAGGAGGGGGAGATCCAATCCTTTACCAGCACTTATTC

>KY275273.1 *Sebastes koreanus*

CCCTCAGCCTACTCATTCGAGCAGAACTAAGCCAACCGGGCGCTCTCCTTGGAGACGACCAAATTTACAATGTAATCGTTACAGCACATGCCTTCGTAATGATTTTCTTTATAGTAATGCCAATTATAATTGGAGGTTTTGGAAACTGATTAATTCCCCTAATGATTGGAGCCCCAGATATAGCATTTCCTCGTATAAATAATATAAGTTTCTGACTTCTTCCCCCTTCTTTCCTGCTACTACTTGCCTCCTCTGGAGTAGAGGCGGGTGCCGGAACCGGGTGAACAGTGTACCCGCCCCTGGCTGGTAATTTAGCCCACGCAGGAGCATCAGTCGACCTGACAATCTTTTCACTCCACCTGGCAGGTATTTCCTCAATCCTTGGGGCAATCAATTTTATTACCACAATTATTAATATGAAACCTCCGGCCATCTCCCAATACCAGACACCCCTGTTTGTGTGGGCCGTCTTAATTACCGCTGTCCTTCTCCTTCTCTCTCTACCAGTCCTCGCTGCTGGCATCACAATACTCCTTACGGACCGGAACCTTAATACCACCTTCTTTGACCCGGCAGGAGGGGGAGATCCAATCCTTTACCAGCACTTATTC

>KY275274.1 *Sebastes koreanus*

CCCTCAGCCTACTCATTCGAGCAGAACTAAGCCAACCGGGCGCTCTCCTTGGAGACGACCAAATTTACAATGTAATCGTTACAGCACATGCCTTCGTAATGATTTTCTTTATAGTAATGCCAATTATAATTGGAGGTTTTGGAAACTGATTAATTCCCCTAATGATTGGAGCCCCAGATATAGCATTTCCTCGTATAAATAATATAAGTTTCTGACTTCTTCCCCCTTCTTTCCTGCTACTACTTGCCTCCTCTGGAGTAGAGGCAGGTGCCGGAACCGGGTGAACAGTGTACCCGCCCCTGGCTGGTAATTTAGCCCACGCAGGAGCATCAGTCGACCTGACAATCTTTTCACTCCACCTGGCAGGTATTTCCTCAATCCTTGGGGCAATCAATTTTATTACCACAATTATTAATATGAAACCTCCGGCCATCTCCCAATACCAGACACCCCTGTTTGTGTGGGCCGTCTTAATTACCGCTGTCCTTCTCCTTCTCTCTCTACCAGTCCTCGCTGCTGGCATCACAATACTCCTTACGGACCGGAACCTTAATACCACCTTCTTTGACCCGGCAGGAGGGGGTGATCCAATCCTTTACCAGCACTTATTC

>KY275275.1 *Sebastes koreanus*

CCCTCAGCCTACTCATTCGAGCAGAACTAAGCCAACCGGGCGCTCTCCTTGGAGACGACCAAATTTACAATGTAATCGTTACAGCACATGCCTTCGTAATGATTTTCTTTATAGTAATGCCAATTATAATTGGAGGTTTTGGAAACTGATTAATTCCCCTAATGATTGGAGCCCCAGATATAGCATTTCCTCGTATAAATAATATAAGTTTCTGACTTCTTCCCCCTTCTTTCCTGCTACTACTTGCCTCCTCTGGAGTAGAGGCAGGTGCCGGAACCGGGTGAACAGTGTACCCGCCCCTGGCTGGTAATTTAGCCCACGCAGGAGCATCAGTCGACCTGACAATCTTTTCACTCCACCTGGCAGGTATTTCCTCAATCCTTGGGGCAATCAATTTTATTACCACAATTATTAATATGAAACCTCCGGCCATCTCCCAATACCAGACACCCCTGTTTGTGTGGGCCGTCTTAATTACCGCTGTCCTTCTTCTCCTCTCTCTACCAGTCCTCGCTGCTGGCATCACAATACTCCTTACGGACCGGAACCTTAATACCACCTTCTTTGACCCGGCAGGAGGGGGAGATCCAATCCTTTACCAGCACTTATTC

>KY275276.1 *Sebastes koreanus*

CCCTCAGCCTACTCATTCGAGCAGAACTAAGCCAACCGGGCGCTCTCCTTGGAGACGACCAAATTTACAATGTAATCGTTACAGCACATGCCTTCGTAATGATTTTCTTTATAGTAATGCCAATTATAATTGGAGGTTTTGGAAACTGATTAATTCCCCTAATGATTGGAGCCCCAGATATAGCATTTCCTCGTATAAATAATATAAGTTTCTGACTTCTTCCCCCTTCTTTCCTGCTACTACTTGCCTCCTCTGGAGTAGAGGCAGGTGCCGGAACCGGGTGAACAGTGTACCCGCCCCTGGCTGGTAATTTAGCCCACGCAGGAGCATCAGTCGACCTGACAATCTTTTCACTCCACCTGGCAGGTATTTCCTCAATCCTTGGGGCAATCAATTTTATTACCACAATTATTAATATGAAACCTCCGGCCATCTCCCAATACCAGACACCCCTGTTTGTGTGGGCCGTCTTAATTACCGCTGTCCTTCTCCTTCTCTCTCTACCAGTCCTCGCTGCTGGCATCACAATACTCCTTACGGACCGGAACCTTAATACCACCTTCTTTGACCCGGCAGGAGGGGGAGATCCAATCCTTTACCAGCACTTATTC

>KY275277.1 *Sebastes koreanus*

CCCTCAGCCTACTCATTCGAGCAGAACTAAGCCAACCGGGCGCTCTCCTTGGAGACGACCAAATTTACAATGTAATCGTTACAGCACATGCCTTCGTAATGATTTTCTTTATAGTAATGCCAATTATAATTGGAGGTTTTGGAAACTGATTAATTCCCCTAATGATTGGAGCCCCAGATATAGCATTTCCTCGTATAAATAATATAAGTTTCTGACTTCTTCCCCCTTCTTTCCTGCTACTACTTGCCTCCTCTGGAGTAGAGGCAGGTGCCGGAACCGGATGAACAGTGTACCCGCCCCTGGCTGGTAATTTAGCCCACGCAGGAGCATCAGTCGACCTGACAATCTTTTCACTCCACCTGGCAGGTATTTCCTCAATCCTTGGGGCAATCAATTTTATTACCACAATTATTAATATGAAACCTCCGGCCATCTCCCAATACCAGACACCCCTGTTTGTGTGGGCCGTCTTAATTACCGCTGTCCTTCTCCTTCTCTCTCTACCAGTCCTCGCTGCTGGCATCACAATACTCCTTACGGACCGGAACCTTAATACCACCTTCTTTGACCCGGCAGGAGGGGGAGATCCAATCCTTTACCAGCACTTATTC

>KY275278.1 *Sebastes koreanus*

CCCTCAGCCTACTCATTCGAGCAGAACTAAGCCAACCGGGCGCTCTCCTTGGAGACGACCAAATTTACAATGTAATCGTTACAGCACATGCCTTCGTAATGATTTTCTTTATAGTAATGCCAATTATAATTGGAGGTTTTGGAAACTGATTAATTCCCCTAATGATTGGAGCCCCAGATATAGCATTTCCTCGTATAAATAATATAAGTTTCTGACTTCTTCCCCCTTCTTTCCTGCTACTACTTGCCTCCTCTGGAGTAGAGGCAGGTGCCGGAACCGGGTGAACAGTGTACCCGCCCCTGGCTGGTAATTTAGCCCACGCAGGAGCATCAGTCGACCTGACAATCTTTTCACTCCACCTGGCAGGTATTTCCTCAATCCTTGGGGCAATCAATTTTATTACCACAATTATTAATATGAAACCTCCGGCCATCTCCCAATACCAGACACCCCTGTTTGTGTGGGCCGTCTTAATTACCGCTGTCCTTCTCCTTCTCTCCCTACCAGTCCTCGCTGCTGGCATCACAATACTCCTTACGGACCGGAACCTTAATACCACCTTCTTTGACCCGGCAGGAGGGGGAGATCCAATCCTTTACCAGCACTTATTC

>KY275279.1 *Sebastes koreanus*

CCCTCAGCCTACTCATTCGAGCAGAACTAAGCCAACCGGGCGCTCTCCTTGGAGACGACCAAATTTACAATGTAATCGTTACAGCACATGCCTTCGTAATGATTTTCTTTATAGTAATGCCAATTATAATTGGAGGTTTTGGAAACTGATTAATTCCCCTAATGATTGGAGCCCCAGATATAGCATTTCCTCGTATAAATAATATAAGTTTCTGACTTCTTCCCCCTTCTTTCCTGCTACTACTTGCCTCCTCTGGAGTAGAGGCAGGTGCCGGAACCGGGTGAACAGTGTACCCGCCCCTGGCTGGTAATTTAGCCCACGCAGGAGCATCAGTCGACCTGACAATCTTTTCACTCCACCTGGCAGGTATTTCCTCAATCCTTGGGGCAATCAATTTTATTACCACAATTATTAATATGAAACCTCCGGCCATCTCCCAATACCAGACACCCCTGTTTGTGTGGGCCGTCTTAATTACCGCTGTCCTTCTCCTTCTCTCTCTACCAGTCCTCGCTGCTGGCATCACAATACTCCTTACGGACCGGAACCTTAATACCACCTTCTTTGACCCGGCAGGAGGGGGTGATCCAATCCTTTACCAGCACTTATTC

>KY275280.1 *Sebastes koreanus*

CCCTCAGCCTACTCATTCGAGCAGAACTAAGCCAACCGGGCGCTCTCCTTGGAGACGACCAAATTTACAATGTAATCGTTACAGCACATGCCTTCGTAATGATTTTCTTTATAGTAATGCCAATTATAATTGGAGGTTTTGGAAACTGATTAATTCCCCTAATGATTGGAGCCCCAGATATAGCATTTCCTCGTATAAATAATATAAGTTTCTGACTTCTTCCCCCTTCTTTCCTGCTACTACTTGCCTCCTCTGGAGTAGAGGCAGGTGCCGGAACCGGGTGAACAGTGTACCCGCCCCTGGCTGGTAATTTAGCCCACGCAGGAGCATCAGTCGACCTGACAATCTTTTCACTCCACCTGGCAGGTATTTCCTCAATCCTTGGGGCAATCAATTTTATTACCACAATTATTAATATGAAACCTCCGGCCATCTCCCAATACCAGACACCCCTGTTTGTGTGGGCCGTCTTAATTACCGCTGTCCTTCTCCTTCTCTCTCTACCAGTCCTCGCTGCTGGCATCACAATACTCCTTACGGACCGGAACCTTAATACCACCTTCTTTGACCCGGCAGGAGGGGGAGATCCAATCCTTTACCAGCACTTATTC

>KU236809.1 *Liparis tanakae*

GAGCTGGAATGGTCGGGACAGGCCTGAGCTTACTAATTCGGGCCGAACTAAGCCAACCCGGAGCCCTTTTAGGCGACGACCAAATCTACAACGTAATTGTTACAGCACATGCTTTCGTAATAATTTTCTTTATAGTGATGCCAATTATAATCGGAGGTTTCGGGAATTGACTTGTCCCCCTAATGATTGGGGCCCCCGATATAGCCTTCCCCCGAATAAATAACATAAGTTTTTGACTTCTCCCTCCCTCTTTCTTGCTCCTCCTTGCCTCTTCTGGCGTAAAAGCAGGGGCCGGAACCGGATGGACTGTCTACCCCCCTCTTGCCGGTAACCTGGCCCACGCTGGAGCTTCTGTAGACCTGACAATCTTCTCACTACATTTAGCAGGGATTTCTTCAATTCTTGGGGCAATTAATTTTATCACAACCATCATCAATATAAAACCCCCCGCTATATCTCAGTACCAAACCCCCCTTTTCGTTTGATCTGTACTTATTACAGCCGTACTTCTCCTTCTCTCTCTCCCTGTTCTTGCTGCCGGCATTACCATACTCCTGACAGACCGAAATCTTAACACAACCTTCTTTGACCCTGCAGGAGGAGGAGACCCAATTCTTTACCAACATCTT

>KU236810.1 *Liparis tanakae*

GAGCTGGAATGGTCGGGACAGGCCTGAGCTTACTAATTCGGGCCGAACTAAGCCAACCCGGAGCCCTTTTAGGCGACGACCAAATCTACAACGTAATTGTTACAGCACATGCTTTCGTAATAATTTTCTTTATAGTGATGCCAATTATAATCGGAGGTTTCGGGAATTGACTTGTCCCCCTAATGATTGGGGCCCCCGATATAGCCTTCCCCCGAATAAATAACATAAGTTTTTGACTTCTCCCTCCCTCTTTCTTGCTCCTCCTTGCCTCTTCTGGCGTAGAAGCAGGGGCCGGAACCGGATGGACTGTCTACCCCCCTCTTGCCGGTAACCTGGCCCACGCTGGAGCTTCTGTAGACCTGACAATCTTCTCACTACATTTAGCAGGGATTTCTTCAATTCTTGGGGCAATTAATTTTATCACAACCATCATCAATATAAAACCCCCCGCTATATCTCAGTACCAAACCCCCCTTTTCGTTTGATCTGTACTTATTACAGCCGTACTTCTCCTTCTCTCTCTCCCTGTTCTTGCTGCCGGCATTACCATACTCCTGACAGACCGAAATCTTAACACAACCTTCTTTGACCCTGCAGGAGGAGGAGACCCAATTCTTTACCAACATCTT

>KY275281.1 *Liparis tanakae*

GAGCTGGAATGGTCGGGACAGGCCTGAGCTTACTAATTCGGGCCGAACTAAGCCAACCCGGAGCCCTTTTAGGCGACGACCAAATCTACAACGTAATTGTTACAGCACATGCTTTCGTAATAATTTTCTTTATAGTGATGCCAATTATAATCGGAGGTTTCGGGAATTGACTTGTCCCCCTAATGATTGGGGCCCCCGATATAGCCTTCCCCCGAATAAATAACATAAGTTTTTGACTTCTCCCTCCCTCTTTCTTGCTCCTCCTTGCCTCTTCTGGCGTAAAAGCAGGGGCCGGAACCGGATGGACTGTCTACCCCCCTCTTGCCGGTAACCTGGCCCACGCTGGAGCTTCTGTAGACCTGACAATCTTCTCACTACATTTAGCAGGGATTTCTTCAATTCTTGGGGCAATTAATTTTATCACAACCATCATCAATATAAAACCCCCCGCTATATCTCAGTACCAAACCCCCCTTTTCGTTTGATCTGTACTTATTACAGCCGTACTTCTCCTTCTCTCTCTCCCTGTTCTTGCTGCCGGCATTACCATACTCCTGACAGACCGAAATCTTAACACAACCTTCTTTGACCCTGCAGGAGGAGGAGACCCAATTCTTTACCAACATCTT

>KY275282.1 *Liparis chefuensis*

TCGGGACGGGCCTGAGCTTACTAATTCGAGCTGAACTTAGTCAGCCCGGAGCCCTTCTAGGCGATGATCAAATTTATAACGTAATTGTTACAGCACATGCTTTCGTAATAATTTTCTTTATAGTAATACCAATTATAATTGGAGGCTTTGGGAATTGACTCGTTCCCCTAATAATCGGAGCCCCTGACATAGCTTTTCCACGGATAAATAATATAAGTTTTTGACTTCTACCTCCTTCTTTCCTGCTCCTCCTCGCCTCTTCTGGCGTAGAAGCAGGGGCCGGGACGGGCTGAACTGTTTATCCCCCTCTTGCTGGTAACTTGGCCCACGCCGGAGCTTCTGTAGACCTAACAATCTTTTCTCTCCATCTAGCAGGGATCTCTTCAATCCTAGGGGCAATTAACTTTATTACAACTATCATTAATATAAAACCTCCAGCGATATCCCAATATCAAACCCCTCTTTTCGTTTGATCTGTACTTATCACCGCTGTACTTCTCCTTCTTTCCCTCCCTGTGCTTGCTGCTGGCATCACTATACTTCTAACAGATCGAAACCTTAATACAACCTTCTTTGACCCTGCAGGAGGAGGAGACCCAATTCTTTATCAACATCTT

>KU236811.1 *Hemitripterus villosus*

TGAGCCGGAATAGTAGGCACAGCCCTGAGCCTCCTAATTCGAGCTGAGCTGAGCCAACCCGGAGCCCTTTTAGGTGACGACCAAATTTATAATGTAATTGTTACGGCTCATGCCTTCGTAATAATTTTCTTTATAGTGATACCAATTATAATCGGGGGCTTCGGAAACTGACTCATTCCTCTAATGATCGGCGCTCCCGATATGGCATTCCCTCGAATAAACAACATGAGCTTTTGACTCCTCCCTCCCTCCTTTCTGCTACTCCTCGCCTCTTCAGGCGTGGAAGCAGGGGCCGGAACCGGGTGAACAGTTTACCCGCCTCTTGCCGGCAACCTCGCACATGCAGGGGCCTCTGTTGATTTAACAATCTTTTCCTTACATTTGGCAGGGATCTCCTCAATTCTAGGGGCAATCAACTTCATTACAACCATCATCAATATAAAACCCCCGGCCATCTCTCAGTACCAAACCCCTCTTTTCGTATGGTCTGTTCTTATCACAGCCGTCCTATTACTTCTTTCCCTCCCGGTTCTTGCTGCTGGCATCACAATGCTTTTAACGGATCGTAATCTTAACACCACCTTCTTCGACCCAGCGGGGGGCGGAGATCCCATCCTTTACCAGCACCTC

>KU236812.1 *Hemitripterus villosus*

TGAGCCGGAATAGTAGGCACAGCCCTGAGCCTCCTAATTCGAGCTGAGCTGAGCCAACCCGGAGCCCTTTTAGGTGACGACCAAATTTATAATGTAATTGTTACGGCTCATGCCTTCGTAATAATTTTCTTTATAGTGATACCAATTATAATCGGGGGCTTCGGAAACTGACTCATTCCTCTAATGATCGGCGCTCCCGATATGGCATTCCCTCGAATAAACAACATGAGCTTTTGACTCCTCCCTCCCTCCTTTCTGCTACTCCTCGCCTCTTCAGGCGTGGAAGCAGGGGCCGGAACCGGGTGAACAGTTTACCCGCCTCTTGCCGGCAACCTCGCACATGCAGGGGCCTCTGTTGATTTAACAATCTTTTCCTTACATTTGGCAGGGATCTCCTCAATTCTAGGGGCAATCAACTTCATTACAACCATCATCAATATAAAACCCCCGGCCATCTCTCAGTACCAAACCCCTCTTTTCGTATGGTCTGTTCTTATCACAGCCGTCCTATTACTTCTTTCCCTCCCGGTTCTTGCTGCTGGCATCACAATGCTTTTAACGGATCGTAATCTTAACACCACCTTCTTCGACCCAGCGGGGGGCGGAGATCCCATCCTTTACCAGCACCTC

>KU236813.1 *Hemitripterus villosus*

TGAGCCGGAATAGTAGGCACAGCCCTGAGCCTCCTAATTCGAGCTGAGCTGAGCCAACCCGGAGCCCTTTTAGGTGACGACCAAATTTATAATGTAATTGTTACGGCTCATGCCTTCGTAATAATTTTCTTTATAGTGATACCAATTATAATCGGGGGCTTCGGAAACTGACTCATTCCTCTAATGATCGGCGCTCCCGATATGGCATTCCCTCGAATAAACAACATGAGCTTTTGACTCCTCCCTCCCTCCTTTCTGCTACTCCTCGCCTCTTCAGGCGTGGAAGCAGGGGCCGGAACCGGGTGAACAGTTTACCCGCCTCTTGCCGGCAACCTCGCACATGCAGGGGCCTCTGTTGATTTAACAATCTTTTCCTTACATTTGGCAGGGATCTCCTCAATTCTAGGGGCAATCAACTTCATTACAACCATCATCAATATAAAACCCCCGGCCATCTCTCAGTACCAAACCCCTCTTTTCGTATGGTCTGTTCTTATCACAGCCGTCCTATTACTTCTTTCCCTCCCGGTTCTTGCTGCTGGCATCACAATGCTTTTAACGGATCGTAATCTTAACACCACCTTCTTCGACCCAGCGGGGGGCGGAGATCCCATCCTTTACCAGCACCTC

>KU236814.1 *Platycephalus indicus*

CTCTATCTAGTATTCGGTGCCTGAGCCGGAATGGTGGGCACCGCCCTAAGCCTGCTCATCCGAGCGGAACTCTGCCAACCCGGCGCTTTACTAGGCGACGATCAAATCTATAATGTGATCGTCACAGCTCATGCCTTTGTAATAATCTTCTTTATAGTGATACCAATTATGATCGGCGGCTTCGGCAACTGGCTGATCCCCCTAATAATTGGCGCGCCAGACATGGCGTTTCCTCGAATAAATAACATAAGCTTCTGACTCCTACCTCCATCCTTCCTGCTCCTCCTAGCCTCGTCGGCTGTAGAAGCTGGGGCAGGTACCGGATGAACAGTCTACCCACCCCTGTCAAGTAATCTTGCCCACGCAGGAGCCTCTGTTGATTTAACAATTTTTTCACTACATTTAGCAGGAATCTCTTCAATTCTGGGGGCCATCAACTTCATTACAACCATCATTAACATGAAACCTATTGCTATTACTCAATACCAGACCCCCCTCTTCGTGTGGTCCGTTCTGATTACGGCTGTCCTCCTTCTCCTCTCCCTGCCTGTCCTAGCTGCTGGCATTACAATGCTACTAACAGACCGAAATCTAGACACCACCTTCTTTGACCCTGCAGGATGGGGGGACCCAATCCTGTACCAACACCTC

>KU236815.1 *Chelidonichthys kumu*

GGTGCCTGAGCTGGCATAGTAGGCACAGCCCTAAGCCTTCTCATCCGAGCAGAGCTAAGCCAGCCCGGAGCCCTTTTAGGGGACGACCAAATCTATAACGTCATTGTTACAGCCCATGCCTTCGTAATGATTTTCTTTATAGTAATGCCAATCATGATCGGAGGCTTCGGAAACTGACTTATCCCCCTAATGATCGGTGCCCCTGATATGGCTTTTCCTCGAATAAACAACATAAGTTTTTGACTTCTGCCCCCCTCCTTCCTACTCCTTCTCGCCTCCTCTGGGGTTGAAGCCGGTGCCGGAACAGGGTGAACTGTCTACCCTCCCTTGGCCGGCAACTTAGCCCATGCGGGGGCCTCTGTAGACCTGACTATCTTCTCCCTTCATCTGGCCGGGATCTCCTCAATCCTTGGTGCAATTAATTTCATCACAACCATTATTAATATGAAACCTCCCGCAATCTCCCAATACCAAACCCCGCTGTTCGTGTGGTCCGTCCTGATTACCGCCGTCCTCCTTCTTCTGTCCCTGCCAGTCCTTGCCGCGGGCATCACAATGCTTCTAACTGACCGCAACCTAAACACCACATTCTTCGACCCTGCCGGAGGAGGAGACCCCATTCTCTATCAACACCTT

>KU236816.1 *Chelidonichthys kumu*

GGTGCCTGAGCTGGCATAGTAGGCACAGCCCTAAGCCTTCTCATCCGAGCAGAGCTAAGCCAGCCCGGAGCCCTTTTAGGGGACGACCAAATCTATAACGTCATTGTTACAGCCCATGCCTTCGTAATGATTTTCTTTATAGTAATGCCAATCATGATCGGAGGCTTCGGAAACTGACTTATCCCCCTAATGATCGGTGCCCCTGATATGGCTTTTCCTCGAATAAACAACATAAGTTTTTGACTTCTGCCCCCCTCCTTCCTACTCCTTCTCGCCTCCTCTGGGGTTGAAGCCGGTGCCGGAACAGGGTGAACTGTCTACCCTCCCTTGGCCGGCAACTTAGCCCATGCGGGGGCCTCTGTAGACCTGACTATCTTCTCCCTTCATCTGGCCGGGATCTCCTCAATCCTTGGTGCAATTAATTTCATCACAACCATTATTAATATGAAACCTCCCGCAATCTCCCAATACCAAACCCCGCTGTTCGTGTGGTCCGTCCTGATTACCGCCGTCCTCCTTCTTCTGTCCCTGCCAGTCCTTGCCGCGGGCATCACAATGCTTCTAACTGACCGCAACCTAAACACCACATTCTTCGACCCTGCCGGAGGAGGAGACCCCATTCTCTATCAACACCTT

>KU236817.1 *Chelidonichthys kumu*

GGTGCCTGAGCTGGCATAGTAGGCACAGCCCTAAGCCTTCTCATCCGAGCAGAGCTAAGCCAGCCCGGAGCCCTTTTAGGGGACGACCAAATCTATAACGTCATTGTTACAGCCCATGCCTTCGTAATGATTTTCTTTATAGTAATGCCAATCATGATCGGAGGCTTCGGAAACTGACTTATCCCCCTAATGATCGGTGCCCCTGATATGGCTTTTCCTCGAATAAACAACATAAGTTTTTGACTTCTGCCCCCCTCCTTCCTACTCCTTCTCGCCTCCTCTGGGGTTGAAGCCGGTGCCGGAACAGGATGAACTGTCTACCCTCCCTTGGCCGGCAACTTAGCCCATGCGGGGGCCTCTGTAGACCTAACTATCTTCTCCCTTCATCTGGCCGGGATCTCCTCAATCCTTGGTGCAATTAATTTCATCACAACCATTATTAATATGAAACCTCCCGCAATCTCCCAATACCAAACCCCGCTGTTCGTGTGGTCCGTCCTGATTACCGCCGTCCTCCTTCTTCTGTCCCTGCCAGTCCTTGCCGCGGGCATCACAATGCTTCTAACTGACCGCAACCTAAACACCACATTCTTCGACCCTGCCGGAGGAGGAGACCCCATTCTCTATCAACACCTT

>KU236818.1 *Chelidonichthys kumu*

GGTGCCTGAGCTGGCATAGTAGGCACAGCCCTAAGCCTTCTCATCCGAGCAGAGCTAAGCCAGCCCGGAGCCCTTTTAGGGGACGACCAAGTCTATAACGTCATTGTTACAGCCCATGCCTTCGTAATGATTTTCTTTATAGTAATGCCAATCATGATCGGAGGCTTCGGAAACTGACTTATCCCCCTAATGATCGGTGCCCCTGATATGGCTTTTCCTCGAATAAACAACATAAGTTTTTGACTTCTGCCCCCCTCCTTCCTACTCCTTCTCGCCTCCTCTGGGGTTGAAGCCGGTGCCGGAACAGGGTGAACTGTCTACCCTCCCTTGGCCGGCAACTTAGCCCATGCGGGGGCCTCTGTAGACCTGACTATCTTCTCCCTTCATCTGGCCGGGATCTCCTCAATCCTTGGTGCAATTAATTTCATCACAACCATTATTAATATGAAACCTCCCGCAATCTCCCAATACCAAACCCCGCTGTTCGTGTGGTCCGTCCTGATTACCGCCGTCCTCCTTCTTCTGTCCCTGCCAGTCCTTGCGGCGGGCATCACAATGCTTCTAACTGACCGCAACCTAAACACCACATTCTTCGACCCTGCCGGAGGAGGAGACCCCATTCTCTATCAACACCTT

>KU236819.1 *Chelidonichthys kumu*

GGTGCCTGAGCTGGCATAGTAGGCACAGCCCTAAGCCTTCTCATCCGAGCAGAGCTAAGCCAGCCCGGAGCCCTTTTAGGGGACGACCAAATCTATAACGTCATTGTTACAGCCCATGCCTTCGTAATGATTTTCTTTATAGTAATGCCAATCATGATCGGAGGCTTCGGAAACTGACTTATCCCCCTAATGATCGGTGCCCCTGATATGGCTTTTCCTCGAATAAACAACATAAGTTTTTGACTTCTGCCCCCCTCCTTCCTACTCCTTCTCGCCTCCTCTGGGGTTGAAGCCGGTGCCGGAACAGGGTGAACTGTCTACCCTCCCTTGGCCGGCAACTTAGCCCATGCGGGGGCCTCTGTAGACCTGACTATCTTCTCCCTTCATCTGGCCGGGATCTCCTCAATCCTTGGTGCAATTAATTTCATCACAACCATTATTAATATGAAACCTCCCGCAATCTCCCAATACCAAACCCCGCTGTTCGTGTGGTCCGTCCTGATTACCGCCGTCCTCCTTCTTCTGTCCCTGCCAGTCCTTGCCGCGGGCATCACAATGCTTCTAACTGACCGCAACCTAAACACCACATTCTTCGACCCTGCCGGAGGAGGAGACCCCATTCTCTATCAACACCTT

>KU236820.1 *Hexagrammos otakii*

GAGCCGGAATAGTGGGCACAGCTCTGAGCCTCTTAATTCGAGCCGAGCTAAGCCAACCCGGAGCCCTCTTGGGGGACGACCAGATTTATAATGTAATTGTTACAGCGCATGCTTTCGTAATAATTTTCTTTATAGTAATGCCAATCATAATCGGGGGTTTCGGAAACTGACTCATCCCTCTAATGATCGGGGCCCCAGATATGGCATTTCCCCGAATGAATAATATGAGTTTTTGACTCCTGCCCCCCTCCTTCCTCCTTCTCCTTGCCTCTTCTGGGGTAGAAGCTGGGGCCGGAACCGGGTGAACCGTTTACCCCCCTCTGTCTGGTAACCTAGCACACGCCGGGGCCTCTGTTGACCTGACAATTTTCTCCCTACATCTTGCAGGGATTTCATCTATTCTAGGTGCAATTAATTTTATCACGACCATTATTAATATGAAACCCCCCGCCATTTCTCAGTACCAAACCCCCCTGTTTGTGTGATCTGTACTAATCACTGCTGTCCTTCTGCTCCTCTCACTACCAGTCCTTGCTGCGGGTATTACTATGCTTTTAACAGATCGGAATCTTAACACCACATTCTTCGACCCAGCAGGCGGTGGTGACCCCATTCTTTACCAACATCT

>KU236821.1 *Hexagrammos otakii*

GAGCCGGAATAGTGGGCACAGCTCTGAGCCTCTTAATTCGAGCCGAGCTAAGCCAACCCGGAGCCCTCTTGGGGGACGACCAGATTTATAATGTAATTGTTACAGCGCATGCTTTCGTAATAATTTTCTTTATAGTAATGCCAATCATAATCGGGGGTTTCGGAAACTGACTCATCCCTCTAATGATCGGGGCCCCAGATATGGCATTTCCCCGAATGAATAATATGAGTTTTTGACTCCTGCCCCCCTCCTTCCTCCTTCTCCTTGCCTCTTCTGGGGTAGAAGCTGGGGCCGGAACCGGGTGAACCGTTTACCCCCCTCTGTCTGGTAACCTAGCACACGCCGGGGCCTCTGTTGACCTGACAATTTTCTCCCTACATCTTGCAGGGATTTCATCTATTCTAGGTGCAATTAATTTTATCACGACCATTATTAATATGAAACCCCCCGCCATTTCTCAGTACCAAACCCCCCTGTTTGTGTGATCTGTACTAATCACTGCTGTCCTTCTGCTCCTCTCACTACCAGTCCTTGCTGCGGGTATTACTATGCTTTTAACAGATCGGAATCTTAACACCACATTCTTCGACCCAGCAGGCGGTGGTGACCCCATTCTTTACCAACATCT

>KU236822.1 *Hexagrammos otakii*

GAGCCGGAATAGTGGGCACAGCTCTGAGCCTCTTAATTCGAGCCGAGCTAAGCCAACCCGGAGCCCTCTTGGGGGACGACCAGATTTATAATGTAATTGTTACAGCGCATGCTTTCGTAATAATTTTCTTTATAGTAATGCCAATCATAATCGGGGGTTTCGGAAACTGACTCATCCCTCTAATGATCGGGGCCCCAGATATGGCATTTCCCCGAATGAATAATATGAGTTTTTGACTCCTGCCCCCCTCCTTCCTCCTTCTCCTTGCCTCTTCTGGGGTAGAAGCTGGGGCCGGAACCGGGTGAACCGTTTACCCCCCTCTGTCTGGTAACCTAGCACACGCCGGGGCCTCTGTTGACCTGACAATTTTCTCCCTACATCTTGCAGGGATTTCATCTATTCTAGGTGCAATTAATTTTATCACGACCATTATTAATATGAAACCCCCCGCCATTTCTCAGTACCAAACCCCCCTGTTTGTGTGATCTGTACTAATCACTGCTGTCCTTCTGCTCCTCTCACTACCAGTCCTTGCTGCGGGTATTACTATGCTTTTAACAGATCGGAATCTTAACACCACATTCTTCGACCCAGCAGGCGGTGGTGACCCCATTCTTTACCAACATCT

>KU236823.1 Hexagrammos otakii

GAGCCGGAATAGTGGGCACAGCTCTGAGCCTCTTAATTCGAGCCGAGCTAAGCCAACCCGGAGCCCTCTTGGGGGACGACCAGATTTATAATGTAATTGTTACAGCGCATGCTTTCGTAATAATTTTCTTTATAGTAATGCCAATCATAATCGGGGGTTTCGGAAACTGACTCATCCCTCTAATGATCGGGGCCCCAGATATGGCATTTCCCCGAATGAATAATATGAGTTTTTGACTCCTGCCCCCCTCCTTCCTCCTTCTCCTTGCCTCTTCTGGGGTAGAAGCTGGGGCCGGAACCGGGTGAACCGTTTACCCCCCTCTGTCTGGTAACCTAGCACACGCCGGGGCCTCTGTTGACCTGACAATTTTCTCCCTACATCTTGCAGGGATTTCATCTATTCTAGGTGCAATTAATTTTATCACGACCATTATTAATATGAAACCCCCCGCCATTTCTCAGTACCAAACCCCCCTGTTTGTGTGATCTGTACTAATCACTGCTGTCCTTCTGCTCCTCTCACTACCAGTCCTTGCTGCGGGTATTACTATGCTTTTAACAGATCGGAATCTTAACACCACATTCTTCGACCCAGCAGGCGGTGGTGACCCCATTCTTTACCAACATCT

>KY275283.1 *Hexagrammos otakii*

GAGCCGGAATAGTGGGCACAGCTCTGAGCCTCTTAATTCGAGCCGAGCTAAGCCAACCCGGAGCCCTCTTGGGGGACGACCAGATTTATAATGTAATTGTTACAGCGCATGCTTTCGTAATAATTTTCTTTATAGTAATGCCAATCATAATCGGGGGTTTCGGAAACTGACTCATCCCTCTAATGATCGGGGCCCCAGATATGGCATTTCCCCGAATGAATAATATGAGTTTTTGACTCCTGCCCCCCTCCTTCCTCCTTCTCCTTGCCTCTTCTGGGGTAGAAGCTGGGGCCGGAACCGGGTGAACCGTTTACCCCCCTCTGTCTGGTAACCTAGCACACGCCGGGGCCTCTGTTGACCTGACAATTTTCTCCCTACATCTTGCAGGGATTTCATCTATTTTAGGTGCAATTAATTTTATCACGACCATTATTAATATGAAACCCCCCGCCATTTCTCAGTACCAAACCCCCCTGTTTGTGTGATCTGTACTAATCACTGCTGTCCTTCTGCTCCTCTCACTACCAGTCCTTGCTGCGGGTATTACTATGCTTTTAACAGATCGGAATCTTAACACCACATTCTTCGACCCAGCAGGCGGTGGTGACCCCATTCTTTACCAACATCTCTTC

>KY275284.1 *Hexagrammos otakii*

GAGCCGGAATAGTGGGCACAGCTCTGAGCCTCTTAATTCGAGCCGAGCTAAGCCAACCCGGAGCCCTCTTGGGGGACGACCAGATTTATAATGTAATTGTTACAGCGCATGCTTTCGTAATAATTTTCTTTATAGTAATGCCAATCATAATCGGGGGTTTCGGAAACTGACTCATCCCTCTAATGATCGGGGCCCCAGATATGGCATTTCCCCGAATGAATAATATGAGTTTTTGACTCCTGCCCCCCTCCTTCCTCCTTCTCCTTGCCTCTTCTGGGGTAGAAGCTGGGGCCGGAACCGGGTGAACCGTTTACCCCCCTCTGTCTGGTAACCTAGCACACGCCGGGGCCTCTGTTGACCTGACAATTTTCTCCCTACATCTTGCAGGGATTTCATCTATTCTAGGTGCAATTAATTTTATCACGACCATTATTAATATGAAACCCCCCGCCATTTCTCAGTACCAAACCCCCCTGTTTGTGTGATCTGTACTAATCACTGCTGTCCTTCTGCTCCTCTCACTACCAGTCCTTGCTGCGGGTATTACTATGCTTTTAACAGATCGGAATCTTAACACCACATTCTTCGACCCAGCAGGCGGTGGTGACCCCATTCTTTACCAACATCTCTTC

>KY275285.1 *Hexagrammos otakii*

GAGCCGGAATAGTGGGCACAGCTCTGAGCCTCTTAATTCGAGCCGAGCTAAGCCAACCCGGAGCCCTCTTGGGGGACGACCAGATTTATAATGTAATTGTTACAGCGCATGCTTTCGTAATAATTTTCTTTATAGTAATGCCAATCATAATCGGGGGTTTCGGAAACTGACTCATCCCTCTAATGATCGGGGCCCCAGATATGGCATTTCCCCGAATGAATAATATGAGTTTTTGACTCCTGCCCCCCTCCTTCCTCCTTCTCCTTGCCTCTTCTGGGGTAGAAGCTGGGGCCGGAACCGGGTGAACCGTTTACCCCCCTCTGTCTGGTAACCTAGCACACGCCGGGGCCTCTGTTGACCTGACAATTTTCTCCCTACATCTTGCAGGGATTTCATCTATTCTAGGTGCAATTAATTTTATCACGACCATTATTAATATGAAACCCCCCGCCATTTCTCAGTACCAAACCCCCCTGTTTGTGTGATCTGTACTAATCACTGCTGTCCTTCTGCTCCTCTCACTACCAGTCCTTGCTGCGGGTATTACTATGCTTTTAACAGATCGGAATCTTAACACCACATTCTTCGACCCAGCAGGCGGTGGTGACCCCATTCTTTACCAACATCTCTTC

>KY275286.1 *Hexagrammos otakii*

GAGCCGGAATAGTGGGCACAGCTCTGAGCCTCTTAATTCGAGCCGAGCTAAGCCAACCCGGAGCCCTCTTGGGGGACGACCAGATTTATAATGTAATTGTTACAGCGCATGCTTTCGTAATAATTTTCTTTATAGTAATGCCAATCATAATCGGGGGTTTCGGAAACTGACTCATCCCTCTAATGATCGGGGCCCCAGATATGGCATTTCCCCGAATGAATAATATGAGTTTTTGACTCCTGCCCCCCTCCTTCCTCCTTCTCCTTGCCTCTTCTGGGGTAGAAGCTGGGGCCGGAACCGGGTGAACCGTTTACCCCCCTCTGTCTGGTAACCTAGCACACGCCGGGGCCTCTGTTGACCTGACAATTTTCTCCCTACATCTTGCAGGGATTTCATCTATTCTAGGTGCAATTAATTTTATCACGACCATTATTAATATGAAACCCCCCGCCATTTCTCAGTACCAAACCCCCCTGTTTGTGTGATCTGTACTAATCACTGCTGTCCTTCTGCTCCTCTCACTACCAGTCCTTGCTGCGGGTATTACTATGCTTTTAACAGATCGGAATCTTAACACCACATTCTTCGACCCAGCAGGCGGTGGTGACCCCATTCTTTACCAACATCTCTTC

>KY275287.1 *Hexagrammos otakii*

GAGCCGGAATAGTGGGCACAGCTCTGAGCCTCTTAATTCGAGCCGAGCTAAGCCAACCCGGAGCCCTCTTGGGGGACGACCAGATTTATAATGTAATTGTTACAGCGCATGCTTTCGTAATAATTTTCTTTATAGTAATACCAATCATAATCGGGGGTTTCGGAAACTGACTCATCCCCCTAATGATCGGGGCCCCAGATATGGCATTTCCCCGAATGAATAATATGAGTTTTTGACTCCTGCCCCCCTCCTTCCTCCTTCTCCTTGCCTCTTCTGGGGTAGAAGCTGGGGCCGGAACCGGGTGAACCGTTTACCCCCCTCTGTCTGGTAACCTAGCACACGCCGGGGCCTCTGTTGACCTAACAATTTTCTCCCTACATCTTGCAGGGATTTCATCTATTCTAGGTGCAATTAATTTTATCACGACCATTATTAATATGAAACCCCCCGCCATTTCTCAGTACCAAACCCCCCTGTTTGTGTGATCTGTACTAATCACTGCTGTCCTTCTGCTCCTCTCACTACCAGTCCTTGCTGCGGGTATTACTATGCTTTTAACAGATCGGAATCTTAACACCACATTCTTCGACCCAGCAGGCGGTGGTGACCCCATTCTTTACCAACATCTCTTC

>KY275288.1 *Hexagrammos otakii*

GAGCCGGAATAGTGGGCACAGCTCTGAGCCTCTTAATTCGAGCCGAGCTAAGCCAACCCGGAGCCCTCTTGGGGGACGACCAGATTTATAATGTAATTGTTACAGCGCATGCTTTCGTAATAATTTTCTTTATAGTAATGCCAATCATAATCGGGGGTTTCGGAAACTGACTCATCCCTCTAATGATCGGGGCCCCAGATATGGCATTTCCCCGAATGAATAATATGAGTTTTTGACTCCTGCCCCCCTCCTTCCTCCTTCTCCTTGCCTCTTCTGGGGTAGAAGCTGGGGCCGGAACCGGGTGAACCGTTTACCCCCCTCTGTCTGGTAACCTAGCACACGCCGGGGCCTCTGTTGACCTGACAATTTTCTCCCTACATCTTGCAGGGATTTCATCTATTCTAGGTGCAATTAATTTTATCACGACCATTATTAATATGAAACCCCCCGCCATTTCTCAGTACCAAACCCCCCTGTTTGTGTGATCTGTACTAATCACTGCTGTCCTTCTGCTCCTCTCACTACCAGTCCTTGCTGCGGGTATTACTATGCTTTTAACAGATCGAAATCTTAACACCACATTCTTCGACCCAGCAGGCGGTGGTGACCCCATTCTTTACCAACATCTCTTC

>KY275289.1 *Hexagrammos agrammus*

CCTTTATCTAGTATTTGGTGCCTGAGCCGGAATAGTGGGCACAGCTCTGAGCCTCCTAATTCGAGCCGAGCTAAGCCAACCCGGAGCCCTCTTGGGGGATGACCAGATTTATAATGTAATTGTTACAGCACATGCTTTCGTAATAATTTTCTTTATAGTAATGCCAATCATAATCGGGGGTTTCGGAAACTGACTCATCCCCCTAATGATCGGAGCCCCAGATATGGCATTTCCCCGAATGAATAATATGAGTTTTTGACTCCTACCCCCCTCTTTCCTCCTTCTCCTTGCCTCTTCTGGGGTAGAAGCTGGGGCCGGGACCGGGTGAACCGTTTACCCCCCTCTGTCTGGTAATCTGGCACACGCCGGAGCCTCTGTTGACTTAACAATCTTCTCCCTTCATCTTGCAGGGATTTCATCTATTCTAGGTGCAATCAATTTTATCACGACCATTATTAATATGAAACCCCCCGCCATTTCTCAGTACCAGACCCCCTTGTTTGTGTGATCTGTACTAATCACAGCTGTCCTTCTGCTCCTCTCACTACCAGTCCTCGCTGCGGGCATTACTATGCTTTTAACAGACCGAAATCTTAACACCACATTCTTCGACCCGGCTGGTGGTGGTGACCCCATTCTTTACCAACACCTCTTC

>KY275290.1 *Hexagrammos agrammus*

CCTTTATCTAGTATTTGGTGCCTGAGCCGGAATAGTGGGCACAGCTCTGAGCCTCCTAATTCGAGCCGAGCTAAGCCAACCCGGAGCCCTCTTGGGGGATGACCAGATTTATAATGTAATTGTTACAGCACATGCTTTCGTAATAATTTTCTTTATAGTAATGCCAATCATAATCGGAGGTTTCGGAAACTGACTCATCCCCCTAATGATCGGAGCCCCAGATATGGCATTTCCCCGAATGAATAATATGAGTTTTTGACTCCTACCCCCCTCTTTCCTCCTTCTCCTTGCCTCTTCTGGGGTAGAAGCTGGGGCCGGGACCGGGTGAACCGTTTACCCCCCTCTGTCTGGTAATCTGGCACACGCCGGAGCCTCTGTTGACTTAACAATCTTCTCCCTTCATCTTGCAGGGATTTCATCTATTCTAGGTGCAATCAATTTTATCACGACCATTATTAATATGAAACCCCCCGCCATTTCTCAGTACCAGACCCCCTTGTTTGTGTGATCTGTACTAATCACAGCTGTCCTTCTGCTCCTCTCACTACCAGTCCTCGCTGCGGGCATTACTATGCTTTTAACAGACCGAAATCTTAACACCACATTCTTCGACCCGGCTGGTGGTGGTGACCCCATTCTTTACCAACACCTCTTC

>KY275291.1 *Hexagrammos agrammus*

CCTTTATCTAGTATTTGGTGCCTGAGCCGGAATAGTGGGCACAGCTCTGAGCCTCCTAATTCGAGCCGAGCTAAGCCAACCCGGAGCCCTCTTGGGGGATGACCAGATTTATAATGTAATTGTTACAGCACATGCTTTCGTAATAATTTTCTTTATAGTAATGCCAATCATAATCGGGGGTTTCGGAAACTGACTCATCCCCCTAATGATCGGAGCCCCAGATATGGCATTTCCCCGAATGAATAATATGAGTTTTTGACTCCTACCCCCCTCTTTCCTCCTTCTCCTTGCCTCTTCTGGGGTAGAAGCTGGGGCCGGGACCGGGTGAACCGTTTACCCCCCTCTGTCTGGTAATCTGGCACACGCCGGAGCCTCTGTTGACTTAACAATCTTCTCCCTTCATCTTGCAGGGATTTCATCTATTCTAGGTGCAATCAATTTTATCACGACCATTATTAATATGAAACCCCCCGCCATTTCTCAGTACCAGACCCCCTTGTTTGTGTGATCTGTACTAATCACAGCTGTCCTTCTGCTCCTCTCACTACCAGTCCTCGCTGCGGGCATTACTATGCTTTTAACAGACCGAAATCTTAACACCACATTCTTCGACCCGGCTGGTGGTGGTGACCCCATTCTTTACCAACACCTCTTC

>KY275292.1 *Hexagrammos agrammus*

CCTTTATCTAGTATTTGGTGCCTGAGCCGGAATAGTGGGCACAGCTCTGAGCCTCCTAATTCGAGCCGAGCTAAGCCAACCCGGAGCCCTCTTGGGGGATGACCAGATTTATAATGTAATTGTTACAGCACATGCTTTCGTAATAATTTTCTTTATAGTAATGCCAATCATAATCGGGGGTTTCGGAAACTGACTCATCCCCCTAATGATCGGAGCCCCAGATATGGCATTTCCCCGAATGAATAATATGAGTTTTTGACTCCTACCCCCCTCTTTCCTCCTTCTCCTTGCCTCTTCTGGGGTAGAAGCTGGGGCCGGGACCGGGTGAACCGTTTACCCCCCTCTGTCTGGTAATCTGGCACACGCCGGAGCCTCTGTTGACTTAACAATCTTCTCCCTTCATCTTGCAGGGATTTCATCTATTCTAGGTGCAATCAATTTTATCACGACCATTATTAATATGAAACCCCCCGCCATTTCTCAGTACCAGACCCCCTTGTTTGTGTGATCTGTACTAATCACAGCTGTCCTTCTGCTCCTCTCACTACCAGTCCTCGCTGCGGGCATTACTATGCTTTTAACAGACCGAAATCTTAACACCACATTCTTCGACCCGGCTGGTGGTGGTGACCCCATTCTTTACCAACACCTCTTC

>KY275293.1 *Hexagrammos agrammus*

CCTTTATCTAGTATTTGGTGCCTGAGCCGGAATAGTGGGCACAGCTCTGAGCCTCCTAATTCGAGCCGAGCTAAGCCAACCCGGAGCCCTCTTGGGGGATGACCAGATTTATAATGTAATTGTTACAGCACATGCTTTCGTAATAATTTTCTTTATAGTAATGCCAATCATAATCGGGGGTTTCGGAAACTGACTCATCCCCCTAATGATCGGAGCCCCAGATATGGCATTTCCCCGAATGAATAATATGAGTTTTTGACTCCTACCCCCCTCTTTCCTCCTTCTCCTTGCCTCTTCTGGGGTAGAAGCTGGGGCCGGGACCGGGTGAACCGTTTACCCCCCTCTGTCTGGTAATCTGGCACACGCCGGAGCCTCTGTTGACTTAACAATCTTCTCCCTTCATCTTGCAGGGATTTCATCTATTCTAGGTGCAATCAATTTTATCACGACCATTATTAATATGAAACCCCCCGCCATTTCTCAGTACCAGACCCCCTTGTTTGTGTGATCTGTACTAATCACAGCTGTCCTTCTGCTCCTCTCACTACCAGTCCTCGCTGCGGGCATTACTATGCTTTTAACAGACCGAAATCTTAACACCACATTCTTCGACCCGGCTGGTGGTGGTGACCCCATTCTTTACCAACACCTCTTC

>KY275294.1 *Hexagrammos agrammus*

CCTTTATCTAGTATTTGGTGCCTGAGCCGGAATAGTGGGCACAGCTCTGAGCCTCCTAATTCGAGCCGAGCTAAGCCAACCCGGAGCCCTCTTGGGGGATGACCAGATTTATAATGTAATTGTTACAGCACATGCTTTCGTAATAATTTTCTTTATAGTAATGCCAATCATAATCGGGGGTTTCGGAAACTGACTCATCCCCCTAATGATCGGAGCCCCAGATATGGCATTTCCCCGAATGAATAATATGAGTTTTTGACTCCTACCCCCCTCTTTCCTCCTTCTCCTTGCCTCTTCTGGGGTAGAAGCTGGGGCCGGGACCGGGTGAACCGTTTACCCCCCTCTGTCTGGTAATCTGGCACACGCCGGAGCCTCTGTTGACTTAACAATCTTCTCCCTTCATCTTGCAGGGATTTCATCTATTCTAGGTGCAATCAATTTTATCACGACCATTATTAATATGAAACCCCCCGCCATTTCTCAGTACCAGACCCCCTTGTTTGTGTGATCTGTACTAATCACAGCTGTCCTTCTGCTCCTCTCACTACCAGTCCTCGCTGCGGGCATTACTATGCTTTTAACAGACCGAAATCTTAACACCACATTCTTCGACCCGGCTGGTGGTGGTGACCCCATTCTTTACCAACACCTCTTC

>KY275295.1 *Hexagrammos agrammus*

CCTTTATCTAGTATTTGGTGCCTGAGCCGGAATAGTGGGCACAGCTCTGAGCCTCCTAATTCGAGCCGAGCTAAGCCAACCCGGAGCCCTCTTGGGGGATGACCAGATTTATAATGTAATTGTTACAGCACATGCTTTCGTAATAATTTTCTTTATAGTAATGCCAATCATAATCGGGGGTTTCGGAAACTGACTCATCCCCCTAATGATCGGAGCCCCAGATATGGCATTTCCCCGAATGAATAATATGAGTTTTTGACTCCTACCCCCCTCTTTCCTCCTTCTCCTTGCCTCTTCTGGGGTAGAAGCTGGGGCCGGGACCGGGTGAACCGTTTACCCCCCTCTGTCTGGTAATCTGGCACACGCCGGAGCCTCTGTTGACTTAACAATCTTCTCCCTTCATCTTGCAGGGATTTCATCTATTCTAGGTGCAATCAATTTTATCACGACCATTATTAATATGAAACCCCCCGCCATTTCTCAGTACCAGACCCCCTTGTTTGTGTGATCTGTACTAATCACAGCTGTCCTTCTGCTCCTCTCACTACCAGTCCTCGCTGCGGGCATTACTATGCTTTTAACAGACCGAAATCTTAACACCACATTCTTCGACCCGGCTGGTGGTGGTGACCCCATTCTTTACCAACACCTCTTC

>KY275296.1 *Hexagrammos agrammus*

CCTTTATCTAGTATTTGGTGCCTGAGCCGGAATAGTGGGCACAGCTCTGAGCCTCCTAATTCGAGCCGAGCTAAGCCAACCCGGAGCCCTCTTGGGGGATGACCAGATTTATAATGTAATTGTTACAGCACATGCTTTCGTAATAATTTTCTTTATAGTAATGCCAATCATAATCGGGGGTTTCGGAAACTGACTCATCCCCCTAATGATCGGAGCCCCAGATATGGCATTTCCCCGAATGAATAATATGAGTTTTTGACTCCTACCCCCCTCTTTCCTCCTTCTCCTTGCCTCTTCTGGGGTAGAAGCTGGGGCCGGGACCGGGTGAACCGTTTACCCCCCTCTGTCTGGTAATCTGGCACACGCCGGAGCCTCTGTTGACTTAACAATCTTCTCCCTTCATCTTGCAGGGATTTCATCTATTCTAGGTGCAATCAATTTTATCACGACCATTATTAATATGAAACCCCCCGCCATTTCTCAGTACCAGACCCCCTTGTTTGTGTGATCTGTACTAATCACAGCTGTCCTTCTGCTCCTCTCACTACCAGTCCTCGCTGCGGGCATTACTATGCTTTTAACAGACCGAAATCTTAACACCACATTCTTCGACCCGGCTGGTGGTGGTGACCCCATTCTTTACCAACACCTCTTC

>KY275297.1 Hexagrammos agrammus

CCTTTATCTAGTATTTGGTGCCTGAGCCGGAATAGTGGGCACAGCTCTGAGCCTCCTAATTCGAGCCGAGCTAAGCCAACCCGGAGCCCTCTTGGGGGATGACCAGATTTATAATGTAATTGTTACAGCACATGCTTTCGTAATAATTTTCTTTATAGTAATGCCAATCATAATCGGGGGTTTCGGAAACTGACTCATCCCCCTAATGATCGGAGCCCCAGATATGGCATTTCCCCGAATGAATAATATGAGTTTTTGACTCCTACCCCCCTCTTTCCTCCTTCTCCTTGCCTCTTCTGGGGTAGAAGCTGGGGCCGGGACCGGGTGAACCGTTTACCCCCCTCTGTCTGGTAATCTGGCACACGCCGGAGCCTCTGTTGACTTAACAATCTTCTCCCTTCATCTTGCGGGGATTTCATCTATTCTAGGTGCAATCAATTTTATCACGACCATTATTAATATGAAACCCCCCGCCATTTCTCAGTACCAGACCCCCTTGTTTGTGTGATCTGTACTAATCACAGCTGTCCTTCTGCTCCTCTCACTACCAGTCCTCGCTGCGGGCATTACTATGCTTTTAACAGACCGAAATCTTAACACCACATTCTTCGACCCGGCTGGTGGTGGTGACCCCATTCTTTACCAACACCTCTTC

>KY275298.1 *Hexagrammos agrammus*

CCTTTATCTAGTATTTGGTGCCTGAGCCGGAATAGTGGGCACAGCTCTGAGCCTCCTAATTCGAGCCGAGCTAAGCCAACCCGGAGCCCTCTTGGGGGATGACCAGATTTATAATGTAATTGTTACAGCACATGCTTTCGTAATAATTTTCTTTATAGTAATGCCAATCATAATCGGGGGTTTCGGAAACTGACTCATCCCCCTAATGATCGGAGCCCCAGATATGGCATTTCCCCGAATGAATAATATGAGTTTTTGACTCCTACCCCCCTCTTTCCTCCTTCTCCTTGCCTCTTCTGGGGTAGAAGCTGGGGCCGGGACCGGGTGAACCGTTTACCCCCCTCTGTCTGGTAATCTGGCACACGCCGGAGCCTCTGTTGACTTAACAATCTTCTCCCTTCATCTTGCAGGGATTTCATCTATTCTAGGTGCAATCAATTTTATCACGACCATTATTAATATGAAACCCCCCGCCATTTCTCAGTACCAGACCCCCTTGTTTGTGTGATCTGTACTAATCACAGCTGTCCTTCTGCTCCTCTCACTACCAGTCCTCGCTGCGGGCATTACTATGCTTTTAACAGACCGAAATCTTAACACCACATTCTTCGACCCGGCTGGTGGTGGTGACCCCATTCTTTACCAACACCTCTTC

>KU236824.1 *Kareius bicoloratus*

GGTGCCTGAGCCGGAATAGTGGGGACAGGCCTAAGTCTACTCATTCGAGCAGAGCTAAGCCAACCTGGGGCTCTCCTGGGAGACGACCAAATTTATAACGTAATCGTCACCGCACACGCCTTTGTAATAATTTTCTTTATAGTAATACCAATTATGATTGGAGGGTTCGGAAACTGGCTTATCCCATTGATAATTGGGGCCCCCGATATGGCCTTCCCTCGAATAAATAACATGAGCTTCTGGCTTCTACCCCCATCCTTTCTGCTTCTCCTAGCCTCTTCAGGTGTTGAAGCCGGGGCGGGGACAGGGTGAACGGTGTATCCCCCACTAGCTGGAAACCTAGCACACGCCGGGGCATCCGTAGACCTCACAATCTTTTCTCTTCACCTTGCTGGGATTTCATCAATTCTAGGAGCAATCAACTTTATTACTACCATCATCAACATGAAACCAACGGCAGTCACTATGTACCAAATCCCGCTATTTGTTTGGGCCGTACTAATTACCGCCGTCCTTCTTCTCCTCTCCCTTCCGGTCCTAGCCGCTGGCATTACAATGCTACTAACAGACCGCAACTTAAACACAACCTTCTTTGACCCTGCTGGAGGGGGTGACCCCATCCTCTACCAACACCTA

>KU236825.1 *Kareius bicoloratus*

GGTGCCTGAGCCGGAATAGTGGGGACAGGCCTAAGTCTACTCATTCGAGCAGAGCTAAGCCAACCTGGGGCTCTCCTGGGAGACGACCAAATTTATAACGTAATCGTCACCGCACACGCCTTTGTAATAATTTTCTTTATAGTAATACCAATTATGATTGGAGGGTTCGGAAACTGGCTTATCCCATTGATAATTGGGGCCCCCGATATGGCCTTCCCTCGAATAAATAACATGAGCTTCTGGCTTCTACCCCCATCCTTTCTGCTTCTCCTAGCCTCTTCAGGTGTTGAAGCCGGGGCGGGAACAGGGTGAACGGTGTATCCCCCACTAGCTGGAAACCTAGCACACGCCGGGGCATCCGTAGACCTCACAATCTTTTCTCTTCACCTTGCTGGGATTTCATCAATTCTAGGAGCAATCAACTTTATTACTACCATCATCAACATGAAACCAACGGCAGTCACTATGTACCAAATCCCGCTATTTGTTTGGGCCGTACTAATTACCGCCGTCCTTCTTCTCCTCTCCCTTCCGGTCCTAGCCGCTGGCATTACAATGCTACTAACAGACCGCAACTTAAACACAACCTTCTTTGACCCTGCTGGAGGGGGTGACCCCATCCTCTACCAACACCTA

>KU236826.1 *Kareius bicoloratus*

GGTGCCTGAGCCGGAATAGTGGGGACAGGCCTAAGTCTACTCATTCGAGCAGAGCTAAGCCAACCTGGGGCTCTCCTGGGAGACGACCAAATTTATAACGTAATCGTCACCGCACACGCCTTTGTAATAATTTTCTTTATAGTAATACCAATTATGATTGGAGGGTTCGGAAACTGGCTTATCCCATTGATAATTGGGGCCCCCGATATGGCCTTCCCTCGAATAAATAACATGAGCTTCTGGCTTCTACCCCCATCCTTTCTGCTTCTCCTAGCCTCTTCAGGTGTTGAAGCCGGGGCGGGAACAGGGTGAACGGTGTATCCCCCACTAGCTGGAAACCTAGCACACGCCGGGGCATCCGTAGACCTCACAATCTTTTCTCTTCACCTTGCTGGGATTTCATCAATTCTAGGAGCAATCAACTTTATTACTACCATCATCAACATGAAACCAACGGCAGTCACTATGTACCAAATCCCGCTATTTGTTTGGGCCGTACTAATTACCGCCGTCCTTCTTCTCCTCTCCCTTCCGGTCCTAGCCGCTGGCATTACAATGCTACTAACAGACCGCAACTTAAACACAACCTTCTTTGACCCTGCTGGAGGGGGTGACCCCATCCTCTACCAACACCTA

>KY275299.1 *Pseudopleuronectes yokohamae*

CCTCTATCTTGTATTTGGTGCCTGAGCCGGAATAGTGGGAACAGGCCTAAGTCTGCTCATTCGAGCAGAGCTAAGCCAACCCGGAGCTCTCCTGGGAGACGACCAAATTTATAACGTAATCGTCACCGCACACGCCTTTGTAATAATCTTCTTTATAGTAATACCAATTATGATCGGAGGGTTCGGAAACTGACTTATTCCATTAATAATTGGGGCCCCCGATATGGCCTTCCCTCGAATAAATAACATGAGCTTCTGACTTCTACCCCCGTCCTTTCTCCTCCTTCTAGCCTCTTCAGGCGTTGAAGCTGGGGCAGGAACAGGATGAACCGTGTATCCCCCACTAGCTGGAAATCTAGCACACGCCGGAGCATCAGTAGACCTCACCATTTTCTCACTTCACCTTGCCGGAATTTCATCAATTCTAGGGGCAATCAACTTTATTACTACCATCATCAACATGAAACCAACAGCAGTCACTATGTACCAAATCCCACTATTTGTCTGGGCCGTACTAATCACCGCCGTTCTTCTTCTTCTTTCCCTACCCGTCCTGGCCGCTGGCATTACAATGCTGCTGACAGACCGCAACCTAAACACAACCTTCTTTGACCCTGCCGGAGGGGGTGACCCCATCCTCTACCAACACCTATTC

>KY275300.1 *Pseudopleuronectes yokohamae*

CCTCTATCTTGTATTTGGTGCCTGAGCCGGAATAGTGGGAACAGGCCTAAGTCTGCTCATTCGAGCAGAGCTAAGCCAACCCGGGGCTCTCCTGGGAGACGACCAAATTTATAACGTAATCGTCACCGCACACGCCTTTGTAATAATCTTCTTTATAGTAATACCAATTATGATCGGAGGGTTCGGAAACTGACTTATTCCATTAATAATTGGGGCCCCCGATATGGCCTTCCCTCGAATAAATAACATGAGCTTCTGACTTCTACCCCCGTCCTTTCTCCTCCTTCTAGCCTCTTCAGGCGTTGAAGCTGGGGCAGGAACAGGATGAACCGTGTATCCCCCACTAGCTGGAAATCTAGCACACGCCGGAGCATCAGTAGACCTCACCATTTTCTCACTTCACCTTGCCGGAATTTCATCAATTCTAGGGGCAATCAACTTTATTACTACCATCATCAACATGAAACCAACAGCAGTCACTATGTACCAAATCCCACTATTTGTCTGGGCCGTACTAATCACCGCCGTTCTTCTTCTTCTTTCCCTACCCGTCCTGGCCGCTGGCATTACAATGCTGCTGACAGACCGCAACCTAAACACAACCTTCTTTGACCCTGCCGGAGGGGGTGACCCCATCCTCTACCAACACCTATTC

>KY275301.1 *Pseudopleuronectes yokohamae*

CCTCTATCTTGTATTTGGTGCCTGAGCCGGAATAGTGGGAACAGGCCTAAGTCTGCTCATTCGAGCAGAGCTAAGCCAACCCGGAGCTCTCCTGGGAGACGACCAAATTTATAACGTAATCGTCACCGCACACGCCTTTGTAATAATCTTCTTTATAGTAATACCAATTATGATCGGAGGGTTCGGAAACTGACTTATTCCATTAATAATTGGGGCCCCCGATATGGCCTTCCCTCGAATAAATAACATGAGCTTCTGACTTCTACCCCCGTCCTTTCTCCTCCTTCTAGCCTCTTCAGGCGTTGAAGCTGGGGCAGGAACAGGATGAACCGTGTATCCCCCACTAGCTGGAAATCTAGCACACGCCGGAGCATCAGTAGACCTCACCATTTTCTCACTTCACCTTGCCGGAATTTCATCAATTCTAGGGGCAATCAACTTTATTACTACCATCATCAACATGAAACCAACAGCAGTCACTATGTACCAAATCCCACTATTTGTCTGGGCCGTACTAATCACCGCCGTTCTTCTTCTTCTTTCCCTACCCGTCCTGGCCGCTGGCATTACAATGCTGCTGACAGACCGCAACCTAAACACAACCTTCTTTGACCCTGCCGGAGGGGGTGACCCCATCCTCTACCAACACCTATTC

>KU236827.1 *Paralichthys olivaceus*

TAGTGGGGACAGCCCTAAGCCTCCTCATTCGGGCAGAACTCAGCCAACCTGGTGCTCTCCTAGGGGACGACCAGATTTATAACGTAATCGTTACCGCACACGCCTTTGTAATAATCTTTTTCATAGTTATACCAATTATGATTGGAGGCTTTGGCAACTGACTTATCCCCCTGATAATCGGTGCCCCAGACATAGCATTCCCTCGAATAAATAATATAAGCTTCTGACTTCTACCCCCTTCATTCCTTCTTCTCCTGGCTTCTTCAGGTGTCGAAGCTGGTGCCGGTACCGGGTGGACTGTCTACCCTCCCCTAGCTAGCAACCTCGCCCATGCTGGAGCCTCAGTAGATCTAACCATCTTTTCACTACACCTTGCAGGTATTTCATCAATTCTGGGAGCTATCAACTTCATTACTACCATTATTAACATGAAACCCACAACTGTCACAATATACCAAATTCCCCTGTTTGTCTGAGCCGTCCTAATTACGGCTGTCCTGCTGCTCCTCTCGCTGCCAGTTTTAGCCGCCGGTATTACAATACTGCTTACAGACCGAAACCTTAATACAACATTCTTTGACCCTGCAGGAGGAGGGGATCCAATCCTCTACCAACACCTG

>KU236828.1 *Paralichthys olivaceus*

TAGTGGGGACAGCCCTAAGCCTCCTCATTCGGGCAGAACTCAGCCAACCTGGTGCTCTCCTAGGGGACGACCAGATTTATAACGTAATCGTTACCGCACACGCCTTTGTAATAATCTTTTTCATAGTTATACCAATTATGATTGGAGGCTTTGGCAACTGACTTATCCCCCTGATAATCGGTGCCCCAGACATAGCATTCCCTCGAATAAATAATATAAGCTTCTGACTTCTACCCCCTTCATTCCTTCTTCTCCTGGCTTCTTCAGGTGTCGAAGCTGGTGCCGGTACCGGGTGGACTGTCTACCCTCCCCTAGCTAGCAACCTCGCCCATGCTGGAGCCTCAGTAGATCTAACCATCTTTTCACTACACCTTGCAGGTATTTCATCAATTCTGGGAGCTATCAACTTCATTACTACCATTATTAACATGAAACCCACAACTGTCACAATATACCAAATTCCCCTGTTTGTCTGAGCCGTCCTAATTACGGCTGTCCTGCTGCTCCTCTCGCTGCCAGTTTTAGCCGCCGGTATTACAATACTGCTTACAGACCGAAACCTTAATACAACATTCTTTGACCCTGCAGGAGGAGGGGATCCAATCCTCTACCAACACCTG

>KU236829.1 *Paralichthys olivaceus*

TAGTGGGGACAGCCCTAAGCCTCCTCATTCGGGCAGAACTCAGCCAACCTGGTGCTCTCCTAGGGGACGACCAGATTTATAACGTAATCGTTACCGCACACGCCTTTGTAATAATCTTTTTCATAGTTATACCAATTATGATTGGAGGCTTTGGCAACTGACTTATCCCCCTGATAATCGGTGCCCCAGACATAGCATTCCCTCGAATAAATAATATAAGCTTCTGACTTCTACCCCCTTCATTCCTTCTTCTCCTGGCTTCTTCAGGTGTCGAAGCTGGTGCCGGTACCGGGTGGACTGTCTACCCTCCCCTAGCTAGCAACCTCGCCCATGCTGGAGCCTCAGTAGATCTAACCATCTTTTCACTACACCTTGCAGGTATTTCATCAATTCTGGGAGCTATCAACTTCATTACTACCATTATTAACATGAAACCCACAACTGTCACAATATACCAAATTCCCCTGTTTGTCTGAGCCGTCCTAATTACGGCTGTCCTGCTGCTCCTCTCGCTGCCAGTTTTAGCCGCCGGTATTACAATACTGCTTACAGACCGAAACCTTAATACAACATTCTTTGACCCTGCAGGAGGAGGGGATCCAATCCTCTACCAACACCTG

>KU236830.1 *Paralichthys olivaceus*

TAGTGGGGACAGCCCTAAGCCTCCTCATTCGGGCAGAACTCAGCCAACCTGGTGCTCTCCTAGGGGACGACCAGATTTATAACGTAATCGTTACCGCACACGCCTTTGTAATAATCTTTTTCATAGTTATACCAATTATGATTGGAGGCTTTGGCAACTGACTTATCCCCCTGATAATCGGTGCCCCAGACATAGCATTCCCTCGAATAAATAATATAAGCTTCTGACTTCTACCCCCTTCATTCCTTCTTCTCCTGGCTTCTTCAGGTGTCGAAGCTGGTGCCGGTACCGGGTGGACTGTCTACCCTCCCCTAGCTAGCAACCTCGCCCATGCTGGAGCCTCAGTAGATCTAACCATCTTTTCACTACACCTTGCAGGTATTTCATCAATTCTGGGAGCTATCAACTTCATTACTACCATTATTAACATGAAACCCACAACTGTCACAATATACCAAATTCCCCTGTTTGTCTGAGCCGTCCTAATTACGGCTGTCCTGCTGCTCCTCTCGCTGCCAGTTTTAGCCGCCGGTATTACAATACTGCTTACAGACCGAAACCTTAATACAACATTCTTTGACCCTGCAGGAGGAGGGGATCCAATCCTCTACCAACACCTG

>KU236831.1 *Paralichthys olivaceus*

TAGTGGGGACAGCCCTAAGCCTCCTCATTCGGGCAGAACTCAGCCAACCTGGTGCTCTCCTAGGGGACGACCAGATTTATAACGTAATCGTTACCGCACACGCCTTTGTAATAATCTTTTTCATAGTTATACCAATTATGATTGGAGGCTTTGGCAACTGACTTATCCCCCTGATAATCGGTGCCCCAGACATAGCATTCCCTCGAATAAATAACATAAGCTTCTGACTTCTACCCCCTTCATTCCTTCTTCTCCTGGCTTCTTCAGGTGTCGAAGCTGGTGCCGGTACCGGGTGGACTGTCTACCCTCCCCTAGCTAGCAACCTCGCCCATGCTGGAGCCTCAGTAGATCTAACCATCTTTTCACTGCACCTTGCAGGTATTTCATCAATTCTGGGAGCTATCAACTTCATTACTACCATTATTAACATGAAACCCACAACTGTCACAATATACCAAATTCCCCTGTTTGTCTGAGCCGTCCTAATTACGGCTGTCCTGCTGCTCCTCTCGCTGCCAGTTTTAGCCGCCGGTATTACAATACTGCTTACAGACCGAAACCTTAATACAACATTCTTTGACCCTGCAGGAGGAGGGGATCCAATCCTCTACCAACACCTG

>KU236832.1 *Paralichthys olivaceus*

TAGTGGGGACAGCCCTAAGCCTCCTCATTCGGGCAGAACTCAGCCAACCTGGTGCTCTCCTAGGGGACGACCAGATTTATAACGTAATCGTTACCGCACACGCCTTTGTAATAATCTTTTTCATAGTTATACCAATTATGATTGGAGGCTTTGGCAACTGACTTATCCCCCTGATAATCGGTGCCCCAGACATAGCATTCCCTCGAATAAATAATATAAGCTTCTGACTTCTACCCCCTTCATTCCTTCTTCTCCTGGCTTCTTCAGGTGTCGAAGCTGGTGCCGGTACCGGGTGGACTGTCTACCCTCCCCTAGCTAGCAACCTCGCCCATGCTGGAGCCTCAGTAGATCTAACCATCTTTTCACTACACCTTGCAGGTATTTCATCAATTCTGGGAGCTATCAACTTCATTACTACCATTATTAACATGAAACCCACAACTGTCACAATATACCAAATTCCCCTGTTTGTCTGAGCCGTCCTAATTACGGCTGTCCTGCTGCTCCTCTCGCTGCCAGTTTTAGCCGCCGGTATTACAATACTGCTTACAGACCGAAACCTTAATACAACATTCTTTGACCCTGCAGGAGGAGGGGATCCAATCCTCTACCAACACCTG

>KU236833.1 *Paralichthys olivaceus*

TAGTGGGGACAGCCCTAAGCCTCCTCATTCGGGCAGAACTCAGCCAACCTGGTGCTCTCCTAGGGGACGACCAGATTTATAACGTAATCGTTACCGCACACGCCTTTGTAATAATCTTTTTCATAGTTATACCAATTATGATTGGAGGCTTTGGCAACTGACTTATCCCCCTGATAATCGGTGCCCCAGACATAGCATTCCCTCGAATAAATAATAAAAGCTTCTGACTTCTACCCCCTTCATTCCTTCTTCTCCTGGCTTCTTCAGGTGTCGAAGCTGGTGCCGGTACCGGGTGGACTGTCTACCCTCCCCTAGCTAGCAACCTCGCCCATGCTGGAGCCTCAGTAGATCTAACCATCTTTTCACTACACCTTGCAGGTATTTCATCAATTCTGGGAGCTATCAACTTCATTACTACCATTATTAACATGAAACCCACAACTGTCACAATATACCAAATTCCCCTGTTTGTCTGAGCCGTCCTAATTACGGCTGTCCTGCTGCTCCTCTCGCTGCCAGTTTTAGCCGCCGGTATTACAATACTGCTTACAGACCGAAACCTTAATACAACATTCTTTGACCCTGCAGGAGGAGGGGATCCAATCCTCTACCAACACCTG

>KU236834.1 *Cynoglossus joyneri*

TTTTTGGCTGAGCCGGAATAGTAGGAACTGCCCTAAGCCTACTCATTCGAGCAGAACTAAGCCAACCCGGCAGCCTACTTGGCGACGACCAAATCTATAATGTAATCGTTACCGCACATGCATTCGTAATGATTTTCTTTATAGTAATGCCTATTATGATTGGAGGCTTCGGAAATTGATTAATTCCACTAATAATCGGAGCCCCAGACATAGCATTTCCACGAATAAATAATATAAGCTTCTGACTTCTCCCTCCTTCTTTCCTCCTTCTTCTTGCTTCCTCTGCTGTAGAGGCCGGAGCTGGTACAGGTTGAACTGTTTACCCACCTCTTGCAGGCAACCTAGCCCATGCTGGTGCATCCGTAGATCTTACCATCTTCTCGCTCCATCTAGCAGGGGTGTCCTCAATTTTAGGGGCAATCAATTTTATTACCACAGTTCTTAATATAAAACCTGAAGGTATAACAATATACCAAGTACCTCTATTTGTATGAGCAGTACTTATTACAGCAGTTCTTTTACTTCTCTCCCTCCCTGTTTTAGCTGCTGGAATTACTATACTACTCACAGATCGAAATCTAAACACCACTTTCTTTGACCCCGCTGGAGGAGGGGATCCAATCCTCTACCAACACCTATTC

>KU236835.1 *Cynoglossus joyneri*

AGCCGGAATAGTAGGAACTGCCCTAAGCCTACTCATTCGAGCAGAACTAAGCCAACCCGGCAGCCTACTTGGCGACGACCAAATCTATAATGTAATCGTTACCGCACATGCATTCGTAATGATTTTCTTTATAGTAATGCCTATTATGATTGGAGGCTTCGGAAATTGATTAATTCCACTAATAATCGGAGCCCCAGACATAGCATTTCCACGAATAAATAATATAAGCTTCTGACTTCTCCCTCCTTCTTTCCTCCTTCTTCTTGCTTCCTCTGCTGTAGAGGCCGGAGCTGGTACAGGTTGAACTGTTTACCCACCTCTTGCAGGCAACCTAGCCCATGCTGGTGCATCCGTAGATCTTACCATCTTCTCGCTCCATCTAGCAGGGGTATCCTCAATTTTAGGGGCAATCAATTTTATTACCACAGTTCTTAATATAAAACCTGAAGGTATAACAATATACCAAGTACCTCTATTTGTATGAGCAGTACTTATTACAGCAGTTCTTTTACTTCTCTCCCTCCCTGTTTTAGCTGCTGGAATTACTATACTACTCACAGATCGAAATCTAAACACCACTTTCTTTGACCCCGCTGGAGGAGGGGATCCAATCCTCTACCAACACCTATTC

>KY275302.1 *Cynoglossus joyneri*

GTAGGAACTGCCCTAAGCCTACTCATTCGAGCAGAACTAAGCCAACCCGGCAGCCTACTTGGCGACGACCAAATCTATAATGTAATCGTTACCGCACATGCATTCGTAATGATTTTCTTTATAGTAATGCCTATTATGATTGGAGGCTTCGGAAATTGATTAATTCCACTAATAATCGGAGCCCCAGACATAGCATTTCCACGAATAAATAATATAAGCTTCTGACTTCTCCCTCCTTCTTTCCTCCTTCTTCTTGCTTCCTCTGCTGTAGAGGCCGGAGCTGGTACAGGTTGAACTGTTTACCCACCTCTTGCAGGCAACCTAGCCCATGCTGGTGCATCCGTAGATCTTACCATCTTCTCGCTCCATCTAGCAGGGGTGTCCTCAATTTTAGGGGCAATCAATTTTATTACCACAGTTCTTAATATAAAACCTGAAGGTATAACAATATACCAAGTACCTCTATTTGTATGAGCAGTACTTATTACAGCAGTTCTTTTACTTCTCTCCCTCCCTGTTTTAGCTGCTGGAATTACTATACTACTCACAGATCGAAATCTAAACACCACTTTCTTTGACCCCGCTGGAGGAGGGGATCCAATCCTCTACCAACACCTA

>KY275303.1 *Cynoglossus joyneri*

GTAGGAACTGCCCTAAGCCTACTCATTCGAGCAGAACTAAGCCAACCCGGCAGCCTACTTGGCGACGACCAAATCTATAATGTAATCGTTACCGCACATGCATTCGTAATGATTTTCTTTATAGTAATGCCTATTATGATTGGAGGCTTCGGAAATTGATTAATTCCACTAATAATCGGAGCCCCAGACATAGCATTTCCACGAATAAATAATATAAGCTTCTGACTTCTCCCTCCTTCTTTCCTCCTTCTTCTTGCTTCCTCTGCTGTAGAGGCCGGAGCTGGTACAGGTTGAACTGTTTACCCACCTCTTGCAGGCAACCTAGCCCATGCTGGTGCATCCGTAGATCTTACCATCTTCTCGCTCCATCTAGCAGGGGTATCCTCAATTTTAGGGGCAATCAATTTTATTACCACAGTTCTTAATATAAAACCTGAAGGTATAACAATATACCAAGTACCTCTATTTGTATGAGCAGTACTTATTACAGCAGTTCTTTTACTTCTCTCCCTCCCTGTTTTAGCTGCTGGAATTACTATACTACTCACAGATCGAAATCTAAACACCACTTTCTTTGACCCCGCTGGAGGAGGGGATCCAATCCTCTACCAACACCTA

>KY275304.1 *Cynoglossus joyneri*

GTAGGAACTGCCCTAAGCCTACTCATTCGAGCAGAACTAAGCCAACCCGGCAGCCTACTTGGCGACGACCAAATCTATAATGTAATCGTTACCGCACATGCATTCGTAATGATTTTCTTTATAGTAATGCCTATTATGATTGGAGGCTTCGGAAATTGACTAATTCCACTAATAATCGGAGCCCCAGACATAGCATTTCCACGAATAAATAATATAAGCTTCTGACTTCTCCCTCCTTCTTTCCTCCTTCTTCTTGCTTCCTCTGCTGTAGAGGCCGGAGCTGGTACAGGTTGAACTGTTTACCCACCTCTTGCAGGCAACCTAGCCCATGCTGGTGCATCCGTAGATCTTACCATCTTCTCGCTCCATCTAGCAGGGGTGTCCTCAATTTTAGGGGCAATCAATTTTATTACCACAGTTCTTAATATAAAACCTGAAGGTATAACAATATATCAAGTACCTCTATTTGTATGAGCAGTACTTATTACAGCAGTTCTTTTACTTCTCTCCCTCCCTGTCTTAGCTGCTGGAATTACTATACTACTCACAGATCGAAATCTAAACACCACTTTCTTTGACCCCGCTGGAGGAGGGGATCCAATCCTCTACCAACACCTA

>KU236836.1 *Cynoglossus lighti*

GAGCCGGAATAGTAGGAACTGCCCTAAGCCTACTCATTCGAGCAGAACTAAGCCAACCCGGCAGCCTACTTGGCGACGACCAAATCTATAATGTAATCGTTACCGCACATGCATTCGTAATGATTTTCTTTATAGTAATGCCTATTATGATTGGAGGCTTCGGAAATTGACTAATTCCACTAATAATCGGAGCCCCAGACATAGCATTTCCACGAATAAATAATATAAGCTTCTGACTTCTCCCTCCTTCTTTCCTCCTTCTTCTTGCTTCCTCTGCTGTAGAGGCCGGAGCTGGTACAGGTTGAACTGTTTACCCACCTCTTGCAGGCAACCTAGCCCATGCTGGTGCATCCGTAGATCTTACCATCTTCTCGCTCCATCTAGCAGGGGTGTCCTCAATTTTAGGGGCAATCAATTTTATTACCACAGTTCTTAATATAAAACCTGAAGGTATAACAATATATCAAGTACCTCTATTTGTATGAGCAGTACTTATTACAGCAGTTCTTTTACTTCTCTCCCTCCCTGTCTTAGCTGCTGGAATTACTATACTACTCACAGATCGAAATCTAAACACCACTTTCTTTGACCCCGCTGGAGGAGGGGATCCAATCCTCTACCAACACCTA

>KU236837.1 *Cynoglossus lighti*

GAGCCGGAATAGTAGGAACTGCCCTAAGCCTACTCATTCGAGCAGAACTAAGCCAACCCGGCAGCCTACTTGGCGACGACCAAATCTATAATGTAATCGTTACCGCACATGCATTCGTAATGATTTTCTTTATAGTAATGCCTATTATGATTGGAGGCTTCGGAAATTGATTAATTCCACTAATAATCGGAGCCCCAGACATAGCATTTCCACGAATAAATAATATAAGCTTCTGACTTCTCCCTCCTTCTTTCCTCCTTCTTCTTGCTTCCTCTGCTGTAGAGGCCGGAGCTGGTACAGGTTGAACTGTTTACCCACCTCTTGCAGGCAACCTAGCCCATGCTGGTGCATCCGTAGATCTTACCATCTTCTCGCTCCATCTAGCAGGGGTGTCCTCAATTTTAGGGGCAATCAATTTTATTACCACAGTTCTTAATATAAAACCTGAAGGTATAACAATATACCAAGTACCTCTATTTGTATGAGCAGTACTCATTACAGCAGTTCTTTTACTTCTCTCCCTCCCTGTTTTAGCTGCTGGAATTACTATACTACTCACAGATCGAAATCTAAACACCACTTTCTTTGACCCCGCTGGAGGAGGGGATCCAATCCTCTACCAACACCTA

>KU236838.1 *Cynoglossus lighti*

GAGCCGGAATAGTAGGAACTGCCCTAAGCCTACTCATTCGAGCAGAACTAAGCCAACCCGGCAGCCTACTTGGCGACGACCAAATCTATAATGTAATCGTTACCGCACATGCATTCGTAATGATTTTCTTTATAGTAATGCCTATTATGATTGGAGGCTTCGGAAATTGATTAATTCCACTAATAATCGGAGCCCCAGACATAGCATTTCCACGAATAAATAATATAAGCTTCTGACTTCTCCCTCCTTCTTTCCTCCTTCTTCTTGCTTCCTCTGCTGTAGAGGCCGGAGCTGGTACAGGTTGAACTGTTTACCCACCTCTTGCAGGCAACCTAGCCCATGCTGGTGCATCCGTAGATCTTACCATCTTCTCGCTCCATCTAGCAGGGGTGTCCTCAATTTTAGGGGCAATCAATTTTATTACCACAGTTCTTAATATAAAACCTGAAGGTATAACAATATACCAAGTACCTCTATTTGTATGAGCAGTACTTATTACAGCAGTTCTTTTACTTCTCTCCCTCCCTGTTCTAGCTGCTGGAATTACTATACTACTCACAGATCGAAATCTAAACACCACTTTCTTTGACCCCGCTGGAGGAGGGGATCCAATCCTCTACCAGCACCTA

>KU236839.1 *Ammodytes personatus*

GGTGCTTGAGCCGCTATGGTGGGGACGGCCCTAAGCCTGCTCATTCGAGCGGAACTTAGCCAACCCGGCGCCCTCCTCGGAGATGACCAAATCTATAACGTAATTGTTACCGCTCATGCATTCGTAATGATTTTCTTTATAGTAATACCAATTATGATTGGTGGTTTTGGAAACTGACTAATCCCCCTAATGATTGGCGCCCCTGATATAGCATTTCCCCGAATAAATAACATAAGCTTTTGACTTCTTCCACCCTCCCTTCTTCTTCTCTTAGCCTCTTCAGGCGTAGAGGCTGGAGCCGGTACCGGTTGAACTGTATACCCCCCTTTGGCCGGAAATCTGGCCCACGCGGGTGCATCTGTTGATTTAACAATCTTCTCCCTGCATTTAGCCGGAATTTCTTCAATTCTTGGAGCAATCAACTTCATCACCACAATTATTAACATGAAGCCTCCCGCTATCTCCCAGTATCAAACACCTTTATTTGTGTGAGCCGTACTGATTACAGCCGTCCTTCTTCTCCTCTCCCTCCCTGTTCTTGCTGCCGGCATTACAATGCTTCTCACAGACCGTAACTTAAATACTACCTTCTTTGATCCTGCAGGAGGGGGAGACCCCATTCTGTACCAACATCTGTTC

>KU236840.1 *Pholis fangi*

CCGGAATAGTGGGCACAGCTCTAAGTCTCCTCATTCGAGCAGAGTTAAGCCAGCCCGGCGCCCTACTAGGCGACGACCAAATTTATAATGTAATTGTTACAGCACATGCATTCGTAATAATTTTCTTTATAGTAATGCCAATCATGATCGGGGGTTTCGGAAACTGGCTTATTCCGCTAATGATCGGGGCCCCAGACATGGCATTTCCCCGTATAAATAACATGAGTTTTTGGCTTCTTCCTCCTTCTTTCCTTCTTCTCCTTGCCTCTTCTGGGGTTGAGGCAGGAGCTGGTACAGGGTGAACGGTGTACCCGCCCCTTTCTGGTAATTTAGCGCACGCCGGGGCCTCTGTTGACTTAACAATCTTTTCTCTTCACCTGGCGGGGATTTCTTCAATTCTTGGGGCAATTAATTTTATCACAACCATTATTAACATGAAACCCCCTGCCATTTCTCAGTATCAAACACCGCTCTTCGTTTGATCCGTACTCATTACCGCCGTTCTTCTGCTCCTCTCACTCCCCGTGCTTGCAGCCGGAATCACCATACTCCTAACAGACCGTAATCTGAACACCACCTTTTTCGACCCCGCAGGAGGGGGCGACCCGATTCTTTACCAACACTTG

>KU236841.1 *Pholis fangi*

CCGGAATAGTGGGCACAGCTCTAAGTCTCCTCATTCGAGCAGAGTTAAGCCAGCCCGGCGCCCTACTAGGCGACGACCAAATTTATAATGTAATTGTTACAGCACATGCATTCGTAATAATTTTCTTTATAGTAATGCCAATCATGATCGGGGGTTTCGGAAACTGGCTTATTCCCCTAATGATCGGGGCCCCAGACATGGCATTTCCCCGTATAAATAACATGAGTTTTTGGCTTCTTCCTCCTTCTTTCCTTCTTCTCCTTGCCTCTTCTGGGGTTGAGGCAGGAGCTGGTACAGGGTGAACGGTGTACCCGCCCCTTTCTGGTAATTTAGCGCACGCCGGGGCCTCTGTTGACTTAACAATCTTTTCTCTTCACCTGGCGGGGATTTCTTCAATTCTTGGGGCAATTAATTTTATCACAACCATTATTAACATGAAACCCCCTGCCATTTCTCAGTATCAAACACCGCTCTTCGTTTGATCCGTACTCATTACCGCCGTTCTTCTGCTCCTCTCACTCCCCGTGCTTGCAGCCGGAATCACCATACTCCTAACAGACCGTAATCTGAACACCACCTTTTTCGACCCCGCAGGAGGGGGCGACCCGATTCTTTACCAACACTTG

>KU236842.1 *Pholis fangi*

CCGGAATAGTGGGCACAGCTCTAAGTCTCCTCATTCGAGCAGAGTTAAGCCAGCCCGGCGCCCTACTAGGCGACGACCAAATTTATAATGTAATTGTTACAGCACATGCATTCGTAATAATTTTCTTTATAGTAATGCCAATCATGATCGGGGGTTTCGGAAACTGGCTTATTCCCCTAATGATCGGGGCCCCAGACATGGCATTTCCCCGTATAAATAACATGAGTTTTTGGCTTCTTCCTCCTTCTTTCCTTCTTCTCCTTGCCTCTTCTGGGGTTGAGGCAGGAGCTGGTACAGGGTGAACGGTGTACCCGCCCCTTTCTGGTAATTTAGCGCACGCCGGGGCCTCTGTTGACTTAACAATCTTTTCTCTTCACCTGGCGGGGATTTCTTCAATTCTTGGGGCAATTAATTTTATCACAACCATTATTAACATGAAACCCCCTGCCATTTCTCAGTATCAAACACCGCTCTTCGTTTGATCCGTACTCATTACCGCCGTTCTTCTGCTCCTCTCACTCCCCGTGCTTGCAGCCGGAATCACCATACTCCTAACAGACCGTAATCTGAACACCACCTTTTTCGACCCCGCAGGAGGGGGCGACCCGATTCTTTACCAACACTTG

>KU236843.1 *Pholis fangi*

CCGGAATAGTGGGCACAGCTCTAAGTCTCCTCATTCGAGCAGAGTTAAGCCAGCCCGGCGCCCTATTAGGCGACGACCAAATTTATAATGTAATTGTTACAGCACATGCATTCGTAATAATTTTCTTTATAGTAATGCCAATCATGATCGGGGGTTTCGGAAACTGGCTTATTCCCCTAATGATCGGGGCCCCAGACATGGCATTTCCCCGTATAAATAACATGAGTTTTTGGCTTCTTCCTCCTTCTTTCCTTCTTCTCCTTGCCTCTTCTGGGGTTGAGGCAGGAGCTGGTACAGGGTGAACGGTGTACCCGCCCCTTTCTGGTAATTTAGCGCACGCCGGGGCCTCTGTTGACTTAACAATCTTTTCTCTTCACCTGGCGGGGATTTCTTCAATTCTTGGGGCAATTAATTTTATCACAACCATTATTAACATGAAACCCCCTGCCATTTCTCAGTATCAAACACCGCTCTTCGTTTGATCCGTACTCATTACCGCCGTTCTTCTGCTCCTCTCACTCCCCGTGCTTGCAGCCGGAATCACCATACTCCTAACAGACCGTAACCTGAACACCACCTTTTTCGACCCCGCAGGAGGGGGCGACCCGATTCTTTACCAACACTTG

>KU236844.1 *Pholis fangi*

CCGGAATAGTGGGCACAGCTCTAAGTCTCCTCATTCGAGCAGAGTTAAGCCAGCCCGGCGCCCTACTAGGCGACGACCAAATTTATAATGTAATTGTTACAGCACATGCATTCGTAATAATTTTCTTTATAGTAATGCCAATCATGATCGGGGGTTTCGGAAACTGGCTTATTCCCCTAATGATCGGGGCCCCAGACATGGCATTTCCCCGTATAAATAACATGAGTTTTTGGCTTCTTCCTCCTTCTTTCCTTCTTCTCCTTGCCTCTTCTGGGGTTGAGGCAGGAGCTGGTACAGGGTGAACGGTGTACCCGCCCCTTTCTGGTAATTTAGCGCACGCCGGGGCCTCTGTTGACTTAACAATCTTTTCTCTTCACCTGGCGGGGATTTCTTCAATTCTTGGGGCAATTAATTTTATCACAACCATTATTAACATGAAACCCCCTGCCATTTCTCAGTATCAAACACCGCTCTTCGTTTGATCCGTACTCATTACCGCCGTTCTTCTGCTCCTCTCACTCCCCGTGCTTGCAGCCGGAATCACCATACTCCTAACAGATCGTAATCTGAACACCACCTTTTTCGACCCCGCAGGAGGGGGCGACCCGATTCTTTACCAACACTTG

>KU236845.1 *Pholis fangi*

CCGGAATAGTCGGCACAGCTCTAAGTCTCCTCATTCGAGCAGAGTTAAGCCAGCCCGGCGCCCTACTAGGCGACGACCAAATTTATAATGTAATTGTTACAGCACATGCATTCGTAATAATTTTCTTTATAGTAATGCCAATCATGATCGGGGGTTTCGGAAACTGGCTTATTCCCCTAATGATCGGGGCCCCAGACATGGCATTTCCCCGTATAAATAACATGAGTTTTTGGCTTCTTCCTCCTTCTTTCCTTCTTCTCCTTGCCTCTTCTGGGGTTGAGGCAGGAGCTGGTACAGGGTGAACGGTGTACCCGCCCCTTTCTGGTAATTTGGCGCACGCCGGGGCCTCTGTTGACTTAACAATCTTTTCTCTTCACCTGGCGGGGATTTCTTCAATTCTTGGGGCAATTAATTTTATCACAACCATTATTAACATGAAACCCCCTGCCATTTCTCAGTATCAAACACCGCTCTTCGTTTGATCCGTACTCATTACCGCCGTTCTTCTGCTCCTCTCACTCCCCGTGCTTGCAGCCGGAATCACCATACTCCTAACAGACCGTAATCTGAACACCACCTTTTTCGACCCCGCAGGAGGGGGCGACCCGATTCTTTACCAACACTTG

>KY275305.1 *Pholis crassispina*

CCTTTATCTAGTATTTGGTGCATGAGCCGGAATAGTGGGCACAGCTCTAAGCCTCCTCATTCGAGCAGAGTTAAGCCAGCCCGGCGCCCTACTAGGCGACGACCAAATTTATAATGTAATTGTTACAGCACATGCATTCGTAATAATTTTCTTTATAGTAATACCAATCATGATCGGGGGCTTCGGAAACTGACTTATTCCCCTAATGATCGGGGCCCCAGACATGGCGTTTCCCCGTATAAATAACATGAGTTTTTGGCTTCTCCCCCCTTCTTTCCTCCTCCTCCTTGCCTCTTCTGGGGTTGAGGCGGGAGCTGGTACAGGGTGGACGGTATACCCGCCCCTTTCTGGTAATTTAGCGCACGCCGGGGCCTCTGTTGATTTAACAATCTTTTCTCTTCACCTGGCAGGGATTTCTTCAATTCTTGGAGCAATTAATTTTATCACAACGATTATTAACATAAAACCCCCTGCCATTTCTCAATACCAGACACCGCTCTTCGTTTGATCCGTACTCATTACCGCCGTTCTTCTGCTCCTTTCACTTCCCGTGCTTGCAGCCGGGATTACTATGCTCCTAACAGATCGTAATCTAAACACTACCTTTTTTGACCCGGCAGGAGGGGGTGACCCAATTCTTTACCAACACTTGTTC

>KY275306.1 *Pholis crassispina*

CCTTTATCTAGTATTTGGTGCATGAGCCGGAATAGTGGGCACAGCTCTAAGCCTCCTCATTCGAGCAGAGTTAAGCCAGCCCGGCGCCCTACTAGGCGACGACCAAATTTATAATGTAATTGTTACAGCACATGCATTCGTAATAATTTTCTTTATAGTAATACCAATCATGATCGGGGGCTTCGGAAACTGACTTATTCCCCTAATGATCGGGGCCCCAGACATGGCGTTTCCCCGTATAAATAACATGAGTTTTTGGCTTCTCCCCCCTTCTTTCCTCCTCCTCCTTGCCTCTTCTGGGGTTGAGGCGGGAGCTGGTACAGGGTGGACGGTATACCCGCCCCTTTCTGGTAATTTAGCGCACGCCGGGGCCTCTGTTGATTTAACAATCTTTTCTCTTCACCTGGCAGGGATTTCTTCAATTCTTGGAGCAATTAATTTTATCACAACGATTATTAACATAAAACCCCCTGCCATTTCTCAATACCAGACACCGCTCTTCGTTTGATCCGTACTCATTACCGCCGTTCTTCTGCTCCTTTCACTTCCCGTGCTTGCAGCCGGGATTACTATGCTCCTAACAGATCGTAATCTAAACACTACCTTTTTTGACCCGGCAGGAGGGGGTGACCCAATTCTTTACCAACACTTGTTC

>KY275307.1 *Pholis crassispina*

CCTTTATCTAGTATTTGGTGCATGAGCCGGAATAGTGGGCACAGCTCTAAGCCTCCTCATTCGAGCAGAGTTAAGCCAGCCCGGCGCCCTACTAGGCGACGACCAAATTTATAATGTAATTGTTACAGCACATGCATTCGTAATAATTTTCTTTATAGTAATACCAATCATGATCGGGGGCTTCGGAAACTGACTTATTCCCCTAATGATCGGGGCCCCAGACATGGCGTTTCCCCGTATAAATAACATGAGTTTTTGGCTTCTCCCCCCTTCTTTCCTCCTCCTCCTTGCCTCTTCTGGGGTTGAGGCGGGAGCTGGTACAGGGTGGACGGTATACCCGCCCCTTTCTGGTAATTTAGCGCACGCCGGGGCCTCTGTTGATTTAACAATCTTTTCTCTTCACCTGGCAGGGATTTCTTCAATTCTTGGAGCAATTAATTTTATCACAACGATTATTAACATAAAACCCCCTGCCATTTCTCAATACCAGACACCGCTCTTCGTTTGATCCGTACTCATTACCGCCGTTCTTCTGCTCCTTTCACTTCCCGTGCTTGCAGCCGGGATTACTATGCTCCTAACAGATCGTAATCTAAACACTACCTTTTTTGACCCGGCAGGAGGGGGTGACCCAATTCTTTACCAACACTTGTTC

>KY275308.1 *Pholis crassispina*

CCTTTATCTAGTATTTGGTGCATGAGCCGGAATAGTGGGCACAGCTCTAAGCCTCCTCATTCGAGCAGAGTTAAGCCAGCCCGGCGCCCTACTAGGCGACGACCAAATTTATAATGTAATTGTTACAGCACATGCATTCGTAATAATTTTCTTTATAGTAATACCAATCATGATCGGGGGCTTCGGAAACTGACTTATTCCCCTAATGATCGGGGCCCCAGACATGGCGTTTCCCCGTATAAATAACATGAGTTTTTGGCTTCTCCCCCCTTCTTTCCTCCTCCTCCTTGCCTCTTCTGGGGTTGAGGCGGGAGCTGGTACAGGGTGGACGGTATACCCGCCCCTTTCTGGTAATTTAGCGCACGCCGGGGCCTCTGTTGATTTAACAATCTTTTCTCTTCACCTGGCGGGGATTTCTTCAATTCTTGGAGCAATTAATTTTATCACAACGATTATTAACATAAAACCTCCTGCCATTTCTCAATACCAGACACCGCTCTTCGTTTGATCCGTACTCATTACCGCCGTTCTTCTGCTCCTTTCACTTCCCGTGCTTGCAGCCGGGATTACTATGCTCCTAACAGATCGTAATCTAAACACTACCTTTTTTGACCCGGCAGGAGGGGGTGACCCAATTCTTTACCAACACTTGTTC

>KY275309.1 *Pholis crassispina*

CCTTTATCTAGTATTTGGTGCATGAGCCGGAATAGTGGGCACAGCTCTAAGCCTCCTCATTCGAGCAGAGTTAAGCCAGCCCGGCGCCCTACTAGGCGACGACCAAATTTATAATGTAATTGTTACAGCACATGCATTCGTAATAATTTTCTTTATAGTAATACCAATCATGATCGGGGGCTTCGGAAACTGACTTATTCCCCTAATGATCGGGGCCCCAGACATGGCGTTTCCCCGTATAAATAACATGAGTTTTTGGCTTCTCCCCCCTTCTTTCCTCCTCCTCCTTGCCTCTTCTGGGGTTGAGGCGGGAGCTGGTACAGGGTGGACGGTATACCCGCCCCTTTCTGGTAATTTAGCGCACGCCGGGGCCTCTGTTGATTTAACAATCTTTTCTCTTCACCTGGCAGGGATTTCTTCAATTCTTGGAGCAATTAATTTTATCACAACGATTATTAACATAAAACCCCCTGCCATTTCTCAATACCAGACACCGCTCTTCGTTTGATCCGTACTCATTACCGCCGTTCTTCTGCTCCTTTCACTTCCCGTGCTTGCAGCCGGGATTACTATGCTCCTAACAGATCGTAATCTAAACACTACCTTTTTTGACCCGGCAGGAGGGGGTGACCCAATTCTTTACCAACACTTGTTC

>KY275310.1 *Pholis crassispina*

CCTTTATCTAGTATTTGGTGCATGAGCCGGAATAGTGGGCACAGCTCTAAGCCTCCTCATTCGAGCAGAGTTAAGCCAGCCCGGCGCCCTACTAGGCGACGACCAAATTTATAATGGACTTGTTACAGCACATGCATTCGTAATAATTTTCTTTATAGTAATACCAATCATGATCGGGGGCTTCGGAAACTGACTTATTCCCCTAATGATCGGGGCCCCAGACATGGCGTTTCCCCGTATAAATAACATGAGTTTTTGGCTTCTCCCCCCTTCTTTCCTCCTCCTCCTTGCCTCTTCTGGGGTTGAGGCGGGAGCTGGTACAGGGTGGACGGTATACCCGCCCCTTTCTGGTAATTTAGCGCACGCCGGGGCCTCTGTTGATTTAACAATCTTTTCTCTTCACCTGGCAGGGATTTCTTCAATTCTTGGAGCAATTAATTTTATCACAACGATTATTAACATAAAACCCCCTGCCATTTCTCAATACCAGACACCGCTCTTCGTTTGATCCGTACTCATTACCGCCGTTCTTCTGCTCCTTTCACTTCCCGTGCTTGCAGCCGGGATTACTATGCTCCTAACAGATCGTAATCTAAACACTACCTTTTTTGACCCGGCAGGAGGGGGTGACCCAATTCTTTACCAACACTTGTTC

>KY275311.1 *Pholis crassispina*

CCTTTATCTAGTATTTGGTGCATGAGCCGGAATAGTGGGCACAGCTCTAAGCCTCCTCATTCGAGCAGAGTTAAGCCAGCCCGGCGCCCTACTAGGCGACGACCAAATTTATAATGTAATTGTTACAGCACATGCATTCGTAATAATTTTCTTTATAGTAATACCAATCATGATTGGGGGCTTCGGAAACTGACTTATTCCCCTAATGATCGGGGCCCCAGACATGGCGTTTCCCCGTATAAATAACATGAGTTTTTGGCTTCTCCCCCCTTCTTTCCTCCTCCTCCTTGCCTCTTCTGGGGTTGAGGCGGGAGCTGGTACAGGGTGGACGGTATACCCGCCCCTTTCTGGTAATTTAGCGCACGCCGGGGCCTCTGTTGATTTAACAATCTTTTCTCTTCACCTGGCGGGGATTTCTTCAATTCTTGGAGCAATTAATTTTATCACAACGATTATTAACATAAAACCTCCTGCCATTTCTCAATACCAGACACCGCTCTTCGTTTGATCCGTACTCATTACCGCCGTTCTTCTGCTCCTTTCACTTCCCGTGCTTGCAGCCGGGATTACTATGCTCCTAACAGATCGTAATCTAAACACTACCTTTTTTGACCCGGCAGGAGGGGGTGACCCAATTCTTTACCAACACTTGTTC

>KY275312.1 *Pholis crassispina*

CCTTTATCTAGTATTTGGTGCATGAGCCGGAATAGTGGGCACAGCTCTAAGCCTCCTCATTCGAGCAGAGTTAAGCCAGCCCGGCGCCCTACTAGGCGACGACCAAATTTATAATGTAATTGTTACAGCACATGCATTCGTAATAATTTTCTTTATAGTAATACCAATCATGATCGGGGGCTTCGGAAACTGACTTATTCCCCTAATGATCGGGGCCCCAGACATGGCGTTTCCCCGTATAAATAACATGAGTTTTTGGCTTCTCCCCCCTTCTTTCCTCCTCCTCCTTGCCTCTTCTGGGGTTGAGGCGGGAGCTGGTACAGGGTGGACGGTATACCCGCCCCTTTCTGGTAATTTAGCGCACGCCGGGGCCTCTGTTGATTTAACAATCTTTTCTCTTCACCTGGCAGGGATTTCTTCAATTCTTGGAGCAATTAATTTTATCACAACGATTATTAACATAAAACCCCCTGCCATTTCTCAATACCAGACACCGCTCTTCGTTTGATCCGTACTCATTACCGCCGTTCTTCTGCTCCTTTCACTTCCCGTGCTTGCAGCCGGGATTACTATGCTCCTAACAGATCGTAATCTAAACACTACCTTTTTTGACCCGGCAGGAGGGGGTGACCCAATTCTTTACCAACACTTGTTC

>KY275313.1 *Pholis crassispina*

CCTTTATCTAGTATTTGGTGCATGAGCCGGAATAGTGGGCACAGCTCTAAGCCTCCTCATTCGAGCAGAGTTAAGCCAGCCCGGCGCCCTACTAGGCGACGACCAAATTTATAATGGAATTGTTACAGCACATGCATTCGGAATAATTTTCTTTATAGTAATACCAATCATGATCGGGGGCTTCGGAAACTGACTTATTCCCCTAATGATCGGGGCCCCAGACATGGCGTTTCCCCGTATAAATAACATGAGTTTTTGGCTTCTCCCCCCTTCTTTCCTCCTCCTCCTTGCCTCTTCTGGGGTTGAGGCGGGAGCTGGTACAGGGTGGACGGTATACCCGCCCCTTTCTGGTAATTTAACGCACGCCGGGGCCTCTGTTGATTTAACAATCTTTTCTCTTCACCTGGCGGGGATTTCTTCAATTCTTGGAGCAATTAATTTTATCACGACGATTATTAACATAAAACCTCCTGCCATTTCTCAATACCAGACACCGCTCTTCGTTTGATCCGTACTCATTACCGCCGTTCTTCTGCTCCTTTCACTTCCCGTGCTTGCAGCCGGGATTACTATGCTCCTAACAGATCGTAATCTAAACACTACCTTTTTTGACCCGGCAGGAGGGGGTGACCCAATTCTTTACCAACACTTGTTC

>KY275314.1 *Pholis crassispina*

CCTTTATCTAGTATTTGGTGCATGAGCCGGAATAGTGGGCACAGCTCTAAGCCTCCTCATTCGAGCAGAGTTAAGCCAGCCCGGCGCCCTACTAGGCGACGACCAAATTTATAATGTAATTGTTACAGCACATGCATTCGTAATAATTTTCTTTATAGTAATACCAATCATGATCGGGGGCTTCGGAAACTGACTTATTCCCCTAATGATCGGGGCCCCAGACATGGCGTTTCCCCGTATAAATAACATGAGTTTTTGGCTTCTCCCCCCTTCTTTCCTCCTCCTCCTTGCCTCTTCTGGGGTTGAGGCGGGAGCTGGTACAGGGTGGACGGTATACCCGCCCCTTTCTGGTAATTTAGCGCACGCCGGGGCCTCTGTTGATTTAACAATCTTTTCTCTTCACCTGGCAGGGATTTCTTCAATTCTTGGAGCAATTAATTTTATCACAACGATTATTAACATAAAACCCCCTGCCATTTCTCAATACCAGACACCGCTCTTCGTTTGATCCGTACTCATTACCGCCGTTCTTCTGCTCCTTTCACTTCCCGTGCTTGCAGCCGGGATTACTATGCTCCTAACAGATCGTAATCTAAACACTACCTTTTTTGACCCGGCAGGAGGGGGTGACCCAATTCTTTACCAACACTTGTTC

>KU236846.1 *Chirolophis japonicus*

TGGTGCATGAGCTGGAATAGTGGGCACAGCTTTAAGCCTCCTCATTCGAGCGGAGCTAAGCCAGCCCGGCGCCCTCTTAGGGGACGACCAGATTTATAACGTAATCGTTACGGCCCATGCGTTCGTAATAATTTTCTTTATAGTAATACCAATCATGATTGGAGGGTTTGGAAACTGACTTATTCCCTTAATGATCGGAGCCCCAGACATAGCATTTCCCCGAATAAACAACATGAGTTTTTGGCTTCTGCCCCCGGCTTTTCTTCTCCTTCTTGCCTCTTCTGGTGTAGAGGCGGGAGCCGGAACTGGGTGAACTGTATACCCCCCTCTTTCGGGTAATTTAGCTCATGCAGGGGCCTCTGTTGACTTAACAATCTTTTCCCTTCACTTAGCAGGGATTTCTTCGATTCTCGGGGCAATCAACTTCATCACAACTATTATTAACATAAAACCTCCTGCCATTTCCCAGTACCAAACACCCCTCTTTGTTTGATCAGTGCTTATCACAGCCGTTCTACTACTTCTTTCACTTCCCGTGCTCGCAGCTGGTATCACAATACTTCTAACAGACCGTAATCTTAACACCACTTTCTTTGACCCCGCCGGAGGAGGGGACCCCATCCTTTACCAACACTTATTC

>KU236847.1 *Chirolophis japonicus*

TGGTGCATGAGCTGGAATAGTAGGCACAGCTTTAAGCCTCCTCATTCGAGCGGAGCTAAGCCAGCCCGGCGCCCTCTTAGGGGACGACCAGATTTATAACGTAATCGTTACGGCCCATGCGTTCGTAATAATTTTCTTTATAGTAATACCAATCATGATTGGAGGGTTTGGAAACTGACTTATTCCCTTAATGATCGGAGCCCCAGACATAGCATTTCCCCGAATAAACAACATGAGTTTTTGGCTTCTGCCCCCGGCTTTTCTTCTCCTTCTTGCCTCTTCTGGTGTAGAGGCGGGAGCCGGAACTGGGTGAACTGTATACCCCCCTCTTTCGGGCAATTTAGCTCATGCAGGGGCCTCTGTTGACTTAACAATCTTTTCCCTTCACTTAGCAGGGATTTCTTCGATTCTCGGGGCAATCAACTTCATCACAACTATTATTAACATAAAACCTCCTGCCATTTCCCAGTACCAAACACCCCTCTTTGTTTGATCAGTGCTTATCACAGCCGTTCTACTACTTCTTTCACTTCCCGTGCTCGCAGCTGGTATCACAATACTTCTAACAGACCGTAATCTTAACACCACTTTCTTTGACCCCGCCGGAGGAGGGGACCCCATCCTTTACCAACACTTATTT

>KU236848.1 *Chirolophis japonicus*

TGGTGCATGAGCTGGAATGGTGGGCACAGCTTTAAGCCTCCTCATTCGAGCGGAGCTAAGCCAGCCCGGCGCCCTCTTAGGGGACGACCAGATTTATAACGTAATCGTTACGGCCCATGCGTTCGTAATAATTTTCTTTATAGTAATACCAATCATGATTGGAGGGTTTGGAAACTGACTTATTCCCTTAATGATCGGAGCCCCAGACATAGCATTTCCCCGAATAAACAACATGAGTTTTTGGCTTCTGCCCCCGGCTTTTCTTCTCCTTCTTGCCTCTTCTGGTGTAGAGGCGGGAGCCGGAACTGGGTGAACTGTATACCCCCCTCTTTCGGGTAATTTAGCTCATGCAGGGGCCTCTGTTGACTTAACAATCTTTTCCCTTCACTTAGCAGGGATTTCTTCGATTCTCGGGGCAATCAACTTCATCACAACTATTATTAACATAAAACCTCCTGCCATTTCCCAGTACCAAACACCCCTCTTTGTTTGATCAGTGCTTATCACAGCCGTTCTACTACTTCTTTCACTTCCCGTGCTCGCAGCTGGTATCACAATACTTCTAACAGACCGTAATCTTAACACCACTTTCTTTGACCCCGCCGGAGGAGGGGACCCCATCCTTTACCAACACTTATTT

>KY275315.1 *Chirolophis japonicus*

TGGTGCATGAGCTGGAATAGTAGGCACAGCTTTAAGCCTCCTCATTCGAGCGGAGCTAAGCCAGCCCGGCGCCCTCTTAGGGGACGACCAGATTTATAACGTAATCGTTACGGCCCATGCGTTCGTAATAATTTTCTTTATAGTAATACCAATCATGATTGGAGGGTTTGGAAACTGACTTATTCCCTTAATGATCGGAGCCCCAGACATAGCATTTCCCCGAATAAACAACATGAGTTTTTGGCTTCTGCCCCCGGCTTTTCTTCTCCTTCTTGCCTCTTCTGGTGTAGAGGCGGGAGCCGGAACTGGGTGAACTGTATACCCCCCTCTTTCGGGCAATTTAGCTCATGCAGGGGCCTCTGTTGACTTAACAATCTTTTCCCTTCACTTAGCAGGGATTTCTTCGATTCTCGGGGCAATCAACTTCATCACAACTATTATTAACATAAAACCTCCTGCCATTTCCCAGTACCAAACACCCCTCTTTGTTTGATCAGTGCTTATCACAGCCGTTCTACTACTTCTTTCACTTCCCGTGCTCGCAGCTGGTATCACAATACTTCTAACAGACCGTAATCTTAACACCACTTTCTTTGACCCCGCCGGAGGAGGGGACCCCATCCTTTACCAACACTTATT

>KY275316.1 *Chirolophis japonicas*

TGGTGCATGAGCTGGAATAGTAGGCACAGCTTTAAGCCTCCTCATTCGAGCGGAGCTAAGCCAGCCCGGCGCCCTCTTAGGGGACGACCAGATTTATAACGTAATCGTTACGGCCCATGCGTTCGTAATAATTTTCTTTATAGTAATACCAATCATGATTGGAGGGTTTGGAAACTGACTTATTCCCTTAATGATCGGAGCCCCAGACATAGCATTTCCCCGAATAAACAACATGAGTTTTTGGCTTCTGCCCCCGGCTTTTCTTCTCCTTCTTGCCTCTTCTGGTGTAGAGGCGGGAGCCGGAACTGGGTGAACTGTATACCCCCCTCTTTCGGGTAATTTAGCTCATGCAGGGGCCTCTGTTGACTTAACAATCTTTTCCCTTCACTTAGCAGGGATTTCTTCGATTCTCGGGGCAATCAACTTCATCACAACTATTATTAACATAAAACCTCCTGCCATTTCCCAGTACCAAACACCCCTCTTTGTTTGATCAGTGCTTATCACAGCCGTTCTACTACTTCTTTCACTTCCCGTGCTCGCAGCTGGTATCACAATACTTCTAACAGACCGTAATCTTAACACCACTTTCTTTGACCCCGCCGGAGGAGGGGACCCCATCCTTTACCAGCACTTATT

>KY275317.1 *Chirolophis japonicus*

TGGTGCATGAGCTGGAATAGTAGGCACAGCTTTAAGCCTCCTCATTCGAGCGGAGCTAAGCCAGCCCGGCGCCCTCTTAGGGGACGACCAGATTTATAACGTAATCGTTACGGCCCATGCGTTCGTAATAATTTTCTTTATAGTAATACCAATCATGATTGGAGGGTTTGGAAACTGACTTATTCCCTTAATGATCGGAGCCCCAGACATAGCATTTCCCCGAATAAACAACATGAGTTTTTGGCTTCTGCCCCCGGCTTTTCTTCTCCTTCTTGCCTCTTCTGGTGTAGAGGCGGGAGCCGGAACTGGGTGAACTGTATACCCCCCTCTTTCGGGCAATTTAGCTCATGCAGGGGCCTCTGTTGACTTAACAATCTTTTCCCTTCACTTAGCAGGGATTTCTTCGATTCTCGGGGCAATCAACTTCATCACAACTATTATTAACATAAAACCTCCTGCCATTTCCCAGTACCAAACACCCCTCTTTGTTTGATCAGTGCTTATCACAGCCGTTCTACTACTTCTTTCACTTCCCGTGCTCGCAGCTGGTATCACAATACTTCTAACAGACCGTAATCTTAACACCACTTTCTTTGACCCCGCCGGAGGAGGGGACCCCATCCTTTACCAACACTTATT

>KY275318.1 *Chirolophis japonicus*

TGGTGCATGAGCTGGAATAGTGGGCACAGCTTTAAGCCTCCTCATTCGAGCGGAGCTAAGCCAGCCCGGCGCCCTCTTAGGGGACGACCAGATTTATAACGTAATCGTTACGGCCCATGCGTTCGTAATAATTTTCTTTATAGTAATACCAATCATGATTGGAGGGTTTGGAAACTGACTTATTCCCTTAATGATCGGAGCCCCAGACATAGCATTTCCCCGAATAAACAACATGAGTTTTTGGCTTCTGCCCCCGGCTTTTCTTCTCCTTCTTGCCTCTTCTGGTGTAGAGGCGGGAGCCGGAACTGGGTGAACTGTATACCCCCCTCTTTCGGGTAATTTAGCTCATGCAGGGGCCTCTGTTGACTTAACAATCTTTTCCCTTCACTTAGCAGGGATTTCTTCGATTCTCGGGGCAATCAACTTCATCACAACTATTATTAACATAAAACCTCCTGCCATTTCCCAGTACCAAACACCCCTCTTTGTTTGATCAGTGCTTATCACAGCCGTTCTACTACTTCTTTCACTTCCCGTGCTCGCAGCTGGTATCACAATACTTCTAACAGACCGTAATCTTAACACCACTTTCTTTGACCCCGCCGGAGGAGGGGACCCCATCCTTTACCAACACTTATT

>KY275319.1 *Chirolophis japonicus*

TGGTGCATGAGCTGGAATAGTGGGCACAGCTTTAAGCCTCCTCATTCGAGCGGAGCTAAGCCAGCCCGGCGCCCTCTTAGGGGACGACCAGATTTATAACGTAATCGTTACGGCCCATGCGTTCGTAATAATTTTCTTTATAGTAATACCAATCATGATTGGAGGGTTTGGAAACTGACTTATTCCCTTAATGATCGGAGCCCCAGACATAGCATTTCCCCGAATAAACAACATGAGTTTTTGGCTTCTGCCCCCGGCTTTTCTTCTCCTTCTTGCCTCTTCTGGTGTAGAGGCGGGAGCCGGAACTGGGTGAACTGTATACCCCCCTCTTTCGGGCAATTTAGCTCATGCAGGGGCCTCTGTTGACTTAACAATCTTTTCCCTTCACTTAGCAGGGATTTCTTCGATTCTCGGGGCAATCAACTTCATCACAACTATTATTAACATAAAACCTCCTGCCATTTCCCAGTACCAAACACCCCTCTTTGTTTGATCAGTGCTTATCACAGCCGTTCTACTACTTCTTTCACTTCCCGTGCTCGCAGCTGGTATCACAATACTTCTAACAGACCGTAATCTTAACACCACTTTCTTTGACCCCGCCGGAGGAGGGGACCCCATCCTTTACCAACACTTATT

>KY275320.1 *Chirolophis japonicus*

TGGTGCATGAGCTGGAATAGTAGGCACAGCTTTAAGCCTCCTCATTCGAGCGGAGCTAAGCCAGCCCGGCGCCCTCTTAGGGGACGACCAGATTTATAACGTAATCGTTACGGCCCATGCGTTCGTAATAATTTTCTTTATAGTAATACCAATCATGATTGGAGGGTTTGGAAACTGACTTATTCCCTTAATGATCGGAGCCCCAGACATAGCATTTCCCCGAATAAACAACATGAGTTTTTGGCTTCTGCCCCCGGCTTTTCTTCTCCTTCTTGCCTCTTCTGGTGTAGAGGCGGGAGCCGGAACTGGGTGAACTGTATACCCCCCTCTTTCGGGTAATTTAGCTCATGCAGGGGCCTCTGTTGACTTAACAATCTTTTCCCTTCACTTAGCAGGGATTTCTTCGATTCTCGGGGCAATCAACTTCATCACAACTATTATTAACATAAAACCTCCTGCCATTTCCCAGTACCAAACACCCCTCTTTGTTTGATCAGTGCTTATCACAGCCGTTCTACTACTTCTTTCACTTCCCGTGCTCGCAGCTGGTATCACAATACTTCTAACAGACCGTAATCTTAACACCACTTTCTTTGACCCCGCCGGAGGAGGGGACCCCATCCTTTACCAACACTTATT

>KY275321.1 *Chirolophis japonicus*

TGGTGCATGAGCTGGAATAGTAGGCACAGCTTTAAGCCTCCTCATTCGAGCGGAGCTAAGCCAGCCCGGCGCCCTCTTAGGGGACGACCAGATTTATAACGTAATCGTTACGGCCCATGCGTTCGTAATAATTTTCTTTATAGTAATACCAATCATGATTGGAGGGTTTGGAAACTGACTTATTCCCTTAATGATCGGAGCCCCAGACATAGCATTTCCCCGAATAAACAACATGAGTTTTTGGCTTCTGCCCCCGGCTTTTCTTCTCCTTCTTGCCTCTTCTGGTGTAGAGGCGGGAGCCGGAACTGGGTGAACTGTATACCCCCCTCTTTCGGGTAATTTAGCTCATGCAGGGGCCTCTGTTGACTTAACAATCTTTTCCCTTCACTTAGCAGGGATTTCTTCGATTCTCGGGGCAATCAACTTCATCACAACTATTATTAACATAAAACCTCCTGCCATTTCCCAGTACCAAACACCCCTCTTTGTTTGATCAGTGCTTATCACAGCCGTTCTACTACTTCTTTCACTTCCCGTGCTCGCAGCTGGTATCACAATACTTCTAACAGACCGTAATCTTAACACCACTTTCTTTGACCCCGCCGGAGGAGGGGACCCCATCCTTTACCAACACTTATT

>KY275322.1 *Ernogrammus hexagrammus*

TGCATGAGCCGGAATAGTAGGCACAGCTCTAAGCCTCCTCATTCGAGCAGAGCTAAGCCAACCCGGAGCCCTCTTAGGAGACGACCAAATCTATAACGTAATTGTTACGGCGCATGCGTTTGTAATAATTTTCTTTATAGTAATGCCAATCATGATCGGAGGGTTTGGAAACTGACTTATCCCACTAATGATTGGAGCCCCAGACATGGCATTTCCACGAATAAACAACATGAGCTTCTGACTTCTCCCTCCCGCTTTCCTTCTTCTCCTTGCCTCTTCTGGTGTAGAAGCTGGAGCCGGAACCGGATGAACAGTTTATCCGCCCCTTTCGGGCAATTTAGCCCACGCTGGGGCCTCTGTTGATTTAACAATTTTTTCCCTTCATTTAGCAGGGATTTCTTCAATTCTGGGGGCAATTAACTTCATTACAACCATTATTAACATGAAACCCCCTGCCATTTCTCAGTATCAAACACCCCTCTTCGTTTGATCAGTACTCATTACGGCCGTTCTTCTTCTTCTCTCTCTCCCTGTACTCGCAGCTGGTATTACAATACTCCTAACAGACCGAAACCTTAACACTACCTTCTTCGACCCCGCCGGAGGAGGTGATCCAATCCTTTACCAACACTTGTTT

>KU236849.1 *Zoarces elongatus*

AGTTGGCACAGCCCTGAGCCTCCTCATCCGAGCGGAGCTAAGCCAACCCGGCGCCCTCCTGGGGGACGACCAGATTTACAATGTTATTGTCACAGCACATGCGTTTGTAATAATCTTCTTTATAGTAATACCAATTATGATCGGAGGGTTCGGAAACTGGCTTGTCCCCTTAATGATCGGGGCCCCGGACATAGCATTTCCCCGCATAAACAACATAAGCTTTTGACTCCTCCCCCCATCCTTTCTCCTCCTCCTTGCCTCTTCTGGGGTAGAGGCGGGAGCTGGCACAGGGTGAACAGTTTATCCCCCTCTTTCTGGAAACTTGGCCCATGCGGGGGCCTCTGTTGATTTGACAATCTTCTCCCTGCACTTAGCGGGAATTTCTTCAATCCTCGGAGCAATTAATTTCATCACAACCATCATCAACATGAACCCCCCTGCGATTTCTCAGTACCAAACACCCCTCTTCGTCTGATCTGTCCTTATCACGGCTGTCCTACTACTCCTCTCCCTCCCAGTGCTTGCAGCCGGCATCACGATGCTCCTGACAGACCGCAACCTCAACACCACCTTCTTCGACCCCGCCGGGGGAGGAGACCCCATCCTCTACCAACACTTA

>KU236850.1 *Zoarces elongatus*

AGTTGGCACAGCCCTGAGCCTCCTCATCCGAGCGGAGCTAAGCCAACCCGGCGCCCTCCTGGGGGACGACCAGATTTACAATGTTATTGTCACAGCACATGCGTTTGTAATAATCTTCTTTATAGTAATACCAATTATGATCGGAGGGTTCGGAAACTGGCTTGTCCCCTTAATGATCGGGGCCCCGGACATAGCATTTCCCCGCATAAACAACATAAGCTTTTGACTCCTTCCCCCATCCTTTCTCCTCCTCCTTGCCTCTTCTGGGGTAGAGGCGGGAGCTGGCACAGGGTGAACAGTTTATCCTCCTCTTTCTGGAAACTTGGCCCATGCCGGGGCCTCTGTTGATTTGACAATCTTCTCCCTGCACTTAGCGGGAATTTCTTCAATCCTCGGAGCAATTAATTTCATCACAACCATCATCAACATGAAACCCCCTGCGATTTCTCAGTACCAAACACCCCTCTTCGTCTGATCTGTCCTTATCACGGCTGTCCTACTACTCCTCTCCCTCCCAGTGCTTGCAGCCGGCATCACGATGCTCCTGACAGACCGCAACCTTAACACCACCTTCTTCGACCCCGCCGGAGGAGGAGACCCCATCCTCTACCAACACTTA

>KU236851.1 *Zoarces elongatus*

AGTTGGCACAGCCCTGAGCCTCCTCATCCGAGCGGAGCTAAGCCAACCCGGCGCCCTCCTGGGGGACGACCAGATTTACAATGTTATTGTCACAGCACATGCGTTTGTAATAATCTTCTTTATAGTAATACCAATTATGATCGGAGGGTTCGGAAACTGGCTTGTCCCCTTAATGATCGGGGCCCCGGACATAGCATTTCCCCGCATAAACAACATAAGCTTTTGACTCCTCCCCCCATCCTTTCTCCTCCTCCTTGCCTCTTCTGGGGTAAAGGCGGGAGCTGGCACAGGGTGAACAGTTTATCCCCCTCTTTCTGGAAACTTGGCCCATGCGGGGGCCTCTGTTGATTTGACAATCTTCTCCCTGCACTTAGCGGGAATTTCTTCAATCCTCGGAGCAATTAATTTCATCACAACCATCATCAACATGAAACCCCCTGCGATTTCTCAGTACCAAACACCCCTCTTCGTCTGATCTGTCCTTATCACGGCTGTCCTACTACTCCTCTCCCTCCCAGTGCTTGCAGCCGGCATCACGATGCTCCTGACAAACCGCAACCTCAACACCACCTTCTTCAACCCCGCCGGGGGAGGAGACCCCATCCTCTACCAACACTTA

>KU236852.1 *Zoarces elongatus*

AGTTGGCACAGCCCTGAGCCTCCTCATCCGAGCGGAGCTAAGCCAACCCGGCGCCCTCCTGGGGGACGACCAGATTTACAATGTTATTGTCACAGCACATGCGTTTGTAATAATCTTCTTTATAGTAATACCAATTATGATCGGAGGGTTCGGAAACTGGCTTGTCCCCTTAATGATCGGGGCCCCGGACATAGCATTTCCCCGCATAAACAACATAAGCTTTTGACTCCTTCCCCCATCCTTTCTCCTCCTCCTTGCCTCTTCTGGGGTAGAGGCGGGAGCTGGCACAGGGTGAACAGTTTATCCTCCTCTTTCTGGAAACTTGGCCCATGCCGGGGCCTCTGTTGATTTGACAATCTTCTCCCTGCACTTAGCGGGAATTTCTTCAATCCTCGGAGCAATTAATTTCATCACAACCATCATCAACATGAAACCCCCTGCGATTTCTCAGTACCAAACACCCCTCTTCGTCTGATCTGTCCTTATCACGGCTGTCCTACTACTCCTCTCCCTCCCAGTGCTTGCAGCCGGCATCACGATGCTCCTGACAGACCGCAACCTCAACACCACCTTCTTCGACCCCGCCGGAGGAGGGGACCCCATCCTCTACCAACACTTA

>KU236853.1 *Zoarces elongatus*

AGTTGGCACAGCCCTGAGCCTCCTCATCCGAGCGGAGCTAAGCCAACCCGGCGCCCTCCTGGGGGACGACCAGATTTACAATGTTATTGTCACAGCACATGCGTTTGTAATAATCTTCTTTATAGTAATACCAATTATGATCGGAGGGTTCGGAAACTGGCTTGTCCCCTTAATGATCGGGGCCCCGGACATAGCATTTCCCCGCATAAACAACATAAGCTTTTGACTCCTTCCCCCATCCTTTCTCCTCCTCCTTGCCTCTTCTGGGGTAGAGGCGGGAGCTGGCACAGGGTGAACAGTTTATCCTCCTCTTTCTGGAAACTTGGCCCATGCCGGGGCCTCTGTTGATTTGACAATCTTCTCCCTGCACTTAGCGGGAATTTCTTCAATCCTCGGAGCAATTAATTTCATCACAACCATCATCAACATGAAACCCCCTGCGATTTCTCAGTACCAAACACCCCTCTTCGTCTGATCTGTCCTTATCACGGCTGTCCTACTACTCCTCTCCCTCCCAGTGCTTGCAGCCGGCATCACGATGCTCCTGACAGACCGCAACCTTAACACCACCTTCTTCGACCCCGCCGGAGGAGGGGACCCCATCCTCTACCAACACTTA

>KU236854.1 *Zoarces elongatus*

AGTTGGCACAGCCCTGAGCCTCCTCATCCGAGCGGAGCTAAGCCAACCCGGCGCCCTCCTGGGGGACGACCAGATTTACAATGTTATTGTCACAGCACATGCGTTTGTAATAATCTTCTTTATAGTAATACCAATTATGATCGGAGGGTTCGGAAACTGGCTTGTCCCCTTAATGATCGGGGCCCCGGACATAGCATTTCCCCGCATAAACAACATAAGCTTTTGACTCCTTCCCCCATCCTTTCTCCTCCTCCTTGCCTCTTCTGGGGTAGAGGCGGGAGCTGGCACAGGGTGAACAGTTTATCCTCCTCTTTCTGGAAACTTGGCCCATGCCGGGGCCTCTGTTGATTTGACAATCTTCTCCCTGCACTTAGCGGGAATTTCTTCAATCCTCGGAGCAATTAATTTCATCACAACCATCATCAACATGAAACCCCCTGCGATTTCTCAGTACCAAACACCCCTCTTCGTCTGATCTGTCCTTATCACGGCTGTCCTACTACTCCTCTCCCTCCCAGTGCTTGCAGCCGGCATCACGATGCTCCTGACAGACCGCAACCTCAACACCACCTTCTTCGACCCCGCCGGAGGAGGGGACCCCATCCTCTACCAACACTTA

>KY275323.1 *Zoarces elongatus*

AGTTGGCACAGCCCTAAGCCTCCTCATCCGAGCGGAGCTAAGCCAACCCGGCGCCCTCCTGGGCGACGACCAGATTTACAATGTTATTGTCACAGCACATGCGTTTGTAATAATCTTCTTTATAGTAATACCAATTATGATCGGAGGGTTCGGAAACTGGCTTGTCCCCTTAATGATCGGGGCCCCGGACATAGCATTTCCCCGCATAAACAACATAAGCTTTTGACTCCTTCCCCCATCCTTTCTCCTCCTCCTTGCCTCTTCTGGGGTAGAGGCTGGAGCTGGCACAGGGTGAACAGTTTATCCTCCTCTTTCTGGAAACTTGGCCCATGCCGGGGCCTCTGTTGATTTGACAATCTTCTCCCTGCACTTAGCGGGAATTTCTTCAATCCTCGGAGCAATTAATTTCATCACAACCATCATCAACATGAAACCCCCTGCGATTTCTCAGTACCAAACACCCCTCTTCGTCTGATCTGTCCTTATCACGGCTGTCCTACTACTCCTCTCCCTCCCAGTGCTTGCAGCCGGCATCACGATGCTCCTGACAGACCGCAACCTCAACACCACCTTCTTCGACCCCGCCGGAGGAGGGGACCCCATCCTCTACCAACACTTA

>KY275324.1 *Zoarces elongatus*

AGTTGGCACAGCCCTGAGCCTCCTCATCCGAGCGGAGCTAAGCCAACCCGGCGCCCTCCTGGGGGACGACCAGATTTACAATGTTATTGTCACAGCACATGCGTTTGTAATAATCTTCTTTATAGTAATACCAATTATGATCGGAGGGTTCGGAAACTGGCTTGTCCCCTTAATGATCGGGGCCCCGGACATAGCATTTCCCCGCATAAACAACATAAGCTTTTGACTCCTTCCCCCATCCTTTCTCCTCCTCCTTGCCTCTTCTGGGGTAGAGGCGGGAGCTGGCACAGGGTGAACAGTTTATCCTCCTCTTTCTGGAAACTTGGCCCATGCCGGGGCCTCTGTTGATTTGACAATCTTCTCCCTGCACTTAGCGGGAATTTCTTCAATCCTCGGAGCAATTAATTTCATCACAACCATCATCAACATGAAACCCCCTGCGATTTCTCAGTACCAAACACCCCTCTTCGTCTGATCTGTCCTTATCACGGCTGTCCTACTACTCCTCTCCCTCCCAGTGCTTGCAGCCGGCATCACGATGCTCCTGACAGACCGCAACCTCAACACCACCTTCTTCGACCCCGCCGGAGGAGGGGACCCCATCCTCTACCAACACTTA

>KY275325.1 *Zoarces elongatus*

AGTTGGCACAGCCCTGAGCCTCCTCATCCGAGCGGAGCTAAGCCAACCCGGCGCCCTCCTGGGGGACGACCAGATTTACAATGTTATTGTCACAGCACATGCGTTTGTAATAATCTTCTTTATAGTAATACCAATTATGATCGGAGGGTTCGGAAACTGGCTTGTCCCCTTAATGATCGGGGCCCCGGACATAGCATTTCCCCGCATAAACAACATAAGCTTTTGACTCCTTCCCCCATCCTTTCTCCTCCTCCTTGCCTCTTCTGGGGTAGAGGCGGGAGCTGGCACAGGGTGAACAGTTTATCCTCCTCTTTCTGGAAACTTGGCCCATGCCGGGGCCTCTGTTGATTTGACAATCTTCTCCCTGCACTTAGCGGGAATTTCTTCAATCCTCGGAGCAATTAATTTCATCACAACCATCATCAACATGAAACCCCCTGCGATTTCTCAGTACCAAACACCCCTCTTCGTCTGATCTGTCCTTATCACGGCTGTCCTACTACTCCTCTCCCTCCCAGTGCTTGCAGCCGGCATCACGATGCTCCTGACAGACCGCAACCTCAACACCACCTTCTTCGACCCCGCCGGAGGAGGGGACCCCATCCTCTACCAACACTTA

>KY275326.1 *Zoarces elongatus*

AGTTGGCACAGCCCTGAGCCTCCTCATCCGAGCGGAGCTAAGCCAACCCGGCGCCCTCCTGGGGGACGACCAGATTTACAATGTTATTGTCACAGCACATGCGTTTGTAATAATCTTCTTTATAGTAATACCAATTATGATCGGAGGGTTCGGAAACTGGCTTGTCCCCTTAATGATCGGGGCCCCGGACATAGCATTTCCCCGCATAAACAACATAAGCTTTTGACTCCTTCCCCCATCCTTTCTCCTCCTCCTTGCCTCTTCTGGGGTAGAGGCGGGAGCTGGCACAGGGTGAACAGTTTATCCTCCTCTTTCTGGAAACTTGGCCCATGCCGGGGCCTCTGTTGATTTGACAATCTTCTCCCTGCACTTAGCGGGAATTTCTTCAATCCTCGGAGCAATTAATTTCATCACAACCATCATCAACATGAAACCCCCTGCGATTTCTCAGTACCAAACACCCCTCTTCGTCTGATCTGTCCTTATCACGGCTGTCCTACTACTCCTCTCCCTCCCAGTGCTTGCAGCCGGCATCACGATGCTCCTGACAGACCGCAACCTTAACACCACCTTCTTCGACCCCGCCGGAGGAGGGGACCCCATCCTCTACCAACACTTA

>KU236855.1 *Chaeturichthys stigmatias*

GCCGGTATAGTAGGCACAGCTTTAAGCCTTCTTATCCGAGCTGAACTTAGCCAACCCGGCGCCCTTTTGGGGGACGACCAGATTTATAATGTTATCGTGACAGCCCATGCCTTTGTAATAATCTTTTTTATAGTCATGCCTATCATAATTGGAGGCTTCGGAAACTGACTGGTTCCTTTAATAATTGGCGCCCCAGATATAGCCTTCCCACGAATAAATAACATGAGTTTTTGACTCCTTCCCCCTTCTTTTCTTCTTTTACTCTCTTCCTCAGGCGTCGAAGCCGGGGCCGGAACCGGGTGAACTGTTTACCCACCCCTAGCAGGAAACCTCGCCCATGCCGGAGCTTCTGTTGACTTAACCATCTTTTCTCTCCACCTTGCAGGTATTTCTTCTATCCTAGGAGCTATCAATTTTATTACAACCATTTTAAACATAAAACCCCCAGCAATAACACAATATCAAACGCCCCTTTTTGTGTGATCTGTGCTAATTACAGCGGTTCTTCTACTCTTATCCCTTCCAGTACTTGCTGCTGGCATTACTATGCTTCTTACAGACCGAAATCTAAATACAACCTTTTTTGATCCTGCAGGAGGGGGAGACCCCATCTTGTACCAACACCTT

>KU236856.1 *Chaeturichthys stigmatias*

GCCGGTATAGTAGGCACAGCTTTAAGCCTTCTTATCCGAGCTGAACTTAGCCAACCCGGCGCCCTTTTGGGGGACGACCAGATTTATAATGTTATCGTGACAGCCCATGCCTTTGTAATAATCTTTTTTATAGTCATGCCTATCATAATTGGAGGCTTCGGAAACTGACTGGTTCCTTTAATAATTGGCGCCCCAGATATAGCCTTCCCACGAATAAATAACATGAGTTTTTGACTCCTTCCCCCCTCTTTTCTTCTTTTACTCTCTTCCTCAGGCGTCGAAGCCGGGGCCGGAACCGGGTGAACTGTTTACCCACCCCTAGCAGGAAACCTCGCCCATGCCGGAGCTTCTGTTGACTTAACCATCTTTTCTCTCCACCTTGCAGGTATTTCCTCTATCCTAGGAGCTATCAATTTTATTACAACCATTTTAAACATAAAACCCCCAGCAATAACACAATATCAAACGCCCCTTTTTGTGTGATCTGTGCTAATTACAGCGGTTCTTCTACTCTTATCCCTTCCAGTACTTGCTGCTGGCATTACTATGCTTCTTACAGACCGAAATCTAAATACAACCTTTTTTGATCCTGCAGGAGGGGGAGACCCCATCTTGTACCAACACCTT

>KY275327.1 *Chaeturichthys stigmatias*

GCCGGTATAGTAGGCACAGCTTTAAGCCTTCTTATCCGAGCTGAACTTAGCCAACCCGGCGCCCTTTTGGGGGACGACCAGATTTATAATGTTATCGTGACAGCCCATGCCTTTGTAATAATCTTTTTTATAGTCATGCCTATCATAATTGGAGGCTTCGGAAACTGACTGGTTCCTTTAATAATTGGCGCCCCAGATATAGCCTTCCCACGAATAAATAACATGAGTTTTTGACTCCTTCCCCCTTCTTTTCTTCTTTTACTCTCTTCCTCAGGCGTCGAAGCCGGGGCCGGAACCGGGTGAACTGTTTACCCACCCCTAGCAGGAAACCTCGCCCATGCCGGAGCTTCTGTTGACTTAACCATCTTTTCTCTCCACCTTGCAGGTATTTCTTCTATCCTAGGAGCTATCAATTTTATTACAACCATTTTAAACATAAAACCCCCAGCAATAACACAATATCAAACGCCCCTTTTTGTGTGATCTGTGCTAATTACAGCGGTTCTTCTACTCTTATCCCTTCCAGTACTTGCTGCTGGCATTACTATGCTTCTTACAGACCGAAATCTAAATACAACCTTTTTTGATCCTGCAGGAGGGGGAGACCCCATCTTGTACCAACACCTT

>KY275328.1 *Chaeturichthys stigmatias*

GCCGGTATAGTAGGCACAGCTTTAAGCCTTCTTATCCGAGCTGAACTTAGCCAACCCGGCGCCCTTTTGGGGGACGACCAGATTTATAATGTTATCGTGACAGCCCATGCCTTTGTAATAATCTTTTTTATAGTCATGCCTATCATAATTGGAGGCTTCGGAAACTGACTGGTTCCTTTAATAATTGGCGCCCCAGATATAGCCTTCCCACGAATAAATAACATGAGTTTTTGACTCCTTCCCCCTTCTTTTCTTCTTTTACTCTCTTCCTCAGGCGTCGAAGCCGGGGCCGGAACCGGGTGAACTGTTTACCCACCCCTAGCAGGAAACCTCGCCCATGCCGGAGCTTCTGTTGACTTAACCATCTTTTCTCTCCACCTTGCAGGTATTTCTTCTATCCTAGGGGCTATCAATTTTATTACAACCATTTTAAACATAAAACCCCCAGCAATAACACAATATCAAACGCCCCTTTTTGTGTGATCTGTGCTAATTACAGCGGTTCTTCTACTCTTATCCCTTCCAGTACTTGCTGCTGGCATTACTATGCTTCTTACAGACCGAAATCTAAATACAACCTTTTTTGATCCTGCAGGAGGGGGAGACCCTATCTTGTACCAACACCTT

>KY275329.1 *Chaeturichthys stigmatias*

GCCGGTATAGTAGGCACAGCTTTAAGCCTTCTTATCCGAGCTGAACTTAGCCAACCCGGCGCCCTTTTGGGGGACGACCAGATTTATAATGTTATCGTGACAGCCCATGCCTTTGTAATAATCTTTTTTATAGTCATGCCTATCATAATTGGAGGCTTCGGAAACTGACTGGTTCCTTTAATAATTGGCGCCCCAGATATAGCCTTCCCACGAATAAATAACATGAGTTTTTGACTCCTTCCCCCTTCTTTTCTTCTTTTACTCTCTTCCTCAGGCGTCGAAGCCGGGGCCGGAACCGGGTGAACTGTTTACCCACCCCTAGCAGGAAACCTCGCCCATGCCGGAGCTTCTGTTGACTTAACCATCTTTTCTCTCCACCTTGCAGGTATTTCTTCTATCCTAGGAGCTATCAATTTTATTACAACCATTTTAAACATAAAACCCCCAGCAATAACACAATATCAAACGCCCCTTTTTGTGTGATCTGTGCTAATTACAGCGGTTCTTCTACTCTTATCCCTTCCAGTACTTGCTGCTGGCATTACTATGCTTCTTACAGACCGAAATCTAAATACAACCTTTTTTGATCCTGCAGGAGGGGGAGACCCCATCTTGTACCAACACCTT

>KY275330.1 *Chaeturichthys stigmatias*

GCCGGTATAGTAGGCACAGCTTTAAGCCTTCTTATCCGAGCTGAACTTAGCCAACCCGGCGCCCTTTTGGGGGACGACCAGATTTATAATGTTATCGTGACAGCCCATGCCTTTGTAATAATCTTTTTTATAGTCATGCCTATCATAATTGGAGGCTTCGGAAACTGACTGGTTCCTTTAATAATTGGCGCCCCAGATATAGCCTTCCCACGAATAAATAACATGAGTTTTTGGCTCCTTCCCCCTTCTTTTCTTCTTTTACTCTCTTCCTCAGGCGTCGAAGCCGGGGCCGGAACCGGGTGAACTGTTTACCCACCCCTAGCAGGAAACCTCGCCCATGCCGGAGCTTCTGTTGACTTAACCATCTTTTCTCTCCACCTTGCAGGTATTTCTTCTATCCTAGGAGCTATCAATTTTATTACAACCATTTTAAACATAAAACCCCCAGCAATAACACAATATCAAACGCCCCTTTTTGTGTGATCTGTGCTAATTACAGCGGTTCTTCTACTCTTATCCCTTCCAGTACTTGCTGCTGGCATTACTATGCTTCTTACAGACCGAAATCTAAATACAACCTTTTTTGATCCTGCAGGAGGGGGAGACCCCATCTTGTACCAACACCTT

>KY275331.1 *Chaeturichthys stigmatias*

GCCGGTATAGTAGGCACAGCTTTAAGCCTTCTTATCCGAGCTGAACTTAGCCAACCCGGCGCCCTTTTGGGGGACGACCAGATTTATAATGTTATCGTGACAGCCCATGCCTTTGTAATAATCTTTTTTATAGTCATGCCTATCATAATTGGAGGCTTCGGAAACTGACTGGTTCCTTTAATAATTGGCGCCCCAGATATAGCCTTCCCACGAATAAATAACATGAGTTTTTGACTCCTTCCCCCTTCTTTTCTTCTTTTACTCTCTTCCTCAGGCGTCGAAGCCGGGGCCGGAACCGGGTGAACTGTTTACCCACCCCTAGCAGGAAACCTCGCCCATGCCGGAGCTTCTGTTGACTTAACCATCTTTTCTCTCCACCTTGCAGGTATTTCTTCTATCCTAGGAGCTATCAATTTTATTACAACCATTTTAAACATAAAACCCCCAGCAATAACACAATATCAAACGCCCCTTTTTGTGTGATCTGTGCTAATTACAGCGGTTCTTCTACTCTTATCCCTTCCAGTACTTGCTGCTGGCATTACTATGCTTCTTACAGACCGAAATCTAAATACAACCTTTTTTGATCCTGCAGGAGGGGGAGACCCCATCTTGTACCAACACCTT

>KY275332.1 *Chaeturichthys stigmatias*

GCCGGTATAGTAGGCACAGCTTTAAGCCTTCTTATCCGAGCTGAACTTAGCCAACCCGGCGCCCTTTTGGGGGACGACCAGATTTATAATGTTATCGTGACAGCCCATGCCTTTGTAATAATCTTTTTTATAGTCATGCCTATCATAATTGGAGGCTTCGGAAACTGACTGGTTCCTTTAATAATTGGCGCCCCAGATATAGCCTTCCCACGAATAAATAACATGAGTTTTTGACTCCTTCCCCCCTCTTTTCTTCTTTTACTCTCTTCCTCAGGCGTCGAAGCCGGGGCCGGAACCGGGTGAACTGTTTACCCACCCCTAGCAGGAAACCTCGCCCATGCCGGAGCTTCTGTTGACTTAACCATCTTTTCTCTCCACCTTGCAGGTATTTCCTCTATCCTAGGAGCTATCAATTTTATTACAACCATTTTAAACATAAAACCCCCAGCAATAACACAATATCAAACGCCCCTTTTTGTGTGATCTGTGCTAATTACAGCGGTTCTTCTACTCTTATCCCTTCCAGTACTTGCTGCTGGCATTACTATGCTTCTTACAGACCGAAATCTAAATACAACCTTTTTTGATCCTGCAGGAGGGGGAGACCCCATCTTGTACCAACACCTT

>KY275333.1 *Synechogobius ommaturus*

TTGGTGCCTGAGCCGGAATAGTAGGCACAGCTCTAAGCCTTTTAATTCGGGCTGAACTGAGCCAGCCTGGGGCTCTTTTAGGGGATGACCAGATTTACAATGTCATCGTGACAGCACATGCATTTGTAATAATTTTCTTTATAGTAATACCAATCATAATTGGAGGGTTTGGGAACTGACTAATCCCCCTAATGATTGGTGCCCCTGATATAGCCTTTCCCCGAATAAACAACATAAGCTTTTGACTCTTACCACCATCTTTCCTACTTCTACTTGCATCCTCTGGCGTTGAAGCTGGAGCTGGTACCGGGTGGACTGTTTACCCACCGCTTGCTAGCAACCTAGCACACGCTGGGGCATCTGTAGACCTAACTATTTTCTCCCTCCACCTAGCAGGTATTTCGTCCATCCTAGGTGCTATCAATTTTATTACCACTATTCTAAATATGAAGCCCCCAGCAATTTCTCAATACCAGACGCCCCTATTTGTATGAGCAGTACTAATTACAGCCGTGCTTCTTCTTCTATCACTCCCTGTGCTTGCCGCTGGCATCACTATGCTTCTAACAGATCGCAACCTAAACACATCCTTCTTTGACCCTGCAGGGGGTGGTGACCCGATTCTTTACCAACACCTTTTC

>KY275334.1 *Synechogobius ommaturus*

TTGGTGCCTGAGCCGGAATAGTAGGCACAGCTCTAAGCCTTTTAATTCGGGCTGAACTGAGCCAGCCTGGGGCTCTTTTAGGGGATGACCAGATTTACAATGTCATCGTGACAGCACATGCATTTGTAATAATTTTCTTTATAGTAATACCAATCATAATTGGGGGGTTTGGGAACTGACTAATCCCCCTAATGATTGGTGCCCCTGATATAGCCTTTCCCCGAATAAACAACATAAGCTTTTGACTCTTACCACCATCTTTCCTACTTCTACTTGCATCCTCTGGCGTTGAAGCTGGAGCTGGTACCGGGTGGACTGTTTACCCACCGCTTGCTAGCAACCTAGCACACGCTGGGGCATCTGTAGACCTAACTATTTTCTCCCTCCACCTAGCAGGTATTTCGTCCATCCTAGGTGCTATCAATTTTATTACCACTATTCTAAATATGAAGCCCCCAGCAATTTCTCAATACCAGACGCCCCTATTTGTATGAGCAGTACTAATTACAGCCGTGCTTCTTCTTCTATCACTCCCTGTGCTTGCCGCTGGCATCACTATGCTTCTAACAGATCGCAACCTAAACACATCCTTCTTTGACCCTGCAGGGGGTGGTGACCCGATTCTTTACCAACACCTT

>KY275335.1 *Synechogobius ommaturus*

TTGGTGCCTGAGCCGGAATAGTAGGCACAGCTCTAAGCCTTTTAATTCGGGCTGAACTGAGCCAGCCTGGGGCTCTTTTAGGGGATGACCAGATTTACAATGTCATCGTGACAGCACATGCATTTGTAATAATTTTCTTTATAGTAATACCAATCATAATTGGAGGGTTTGGGAACTGACTAATCCCCCTAATGATTGGTGCCCCTGATATAGCCTTTCCCCGAATAAACAACATAAGCTTTTGACTCTTACCACCATCTTTCCTACTTCTACTTGCATCCTCTGGCGTTGAAGCTGGAGCTGGTACCGGGTGGACTGTTTACCCACCGCTTGCTAGCAACCTAGCACACGCTGGGGCATCTGTAGACCTAACTATTTTCTCCCTCCACCTAGCAGGTATTTCGTCCATCCTAGGTGCTATCAATTTTATTACCACTATTCTAAATATGAAGCCCCCAGCAATTTCTCAATACCAGACGCCCCTATTTGTATGAGCAGTACTAATTACAGCCGTGCTTCTTCTTCTATCACTCCCTGTGCTTGCCGCTGGCATCACTATGCTTCTAACAGATCGCAACCTAAACACATCCTTCTTTGACCCTGCAGGGGGTGGTGACCCGATTCTTTACCAACACCTT

>KY275336.1 *Synechogobius ommaturus*

TTGGTGCCTGAGCCGGAATAGTAGGCACAGCTCTAAGCCTTTTAATTCGGGCTGAACTGAGCCAGCCTGGGGCTCTTTTAGGGGATGACCAGATTTACAATGTCATCGTGACAGCACATGCATTTGTAATAATTTTCTTTATAGTAATACCAATCATAATTGGAGGGTTTGGGAACTGACTAATCCCCCTAATGATTGGTGCCCCTGATATAGCCTTTCCCCGAATAAACAACATAAGCTTTTGACTCTTACCACCATCTTTCCTACTTCTACTTGCATCCTCTGGCGTTGAAGCTGGAGCTGGTACCGGGTGGACTGTTTACCCACCGCTTGCTAGCAACCTAGCACACGCTGGGGCATCTGTAGACCTAACTATTTTCTCCCTCCACCTAGCAGGTATTTCGTCCATCCTAGGTGCTATCAATTTTATTACCACTATTCTAAATATGAAACCCCCAGCAATTTCTCAATACCAGACGCCCCTATTTGTATGAGCAGTACTAATTACAGCCGTGCTTCTTCTTCTATCACTCCCTGTGCTTGCCGCTGGCATCACTATGCTTCTAACAGATCGCAACCTAAACACATCCTTCTTTGACCCTGCAGGGGGTGGTGACCCGATTCTTTACCAACACCTTTTC

>KY275337.1 *Synechogobius ommaturus*

TTGGTGCCTGAGCCGGAATAGTAGGCACAGCTCTAAGCCTTTTAATTCGGGCTGAACTGAGCCAGCCTGGGGCTCTTTTAGGGGATGACCAGATTTACAATGTCATCGTGACAGCACATGCATTTGTAATAATTTTCTTTATAGTAATACCAATCATAATTGGAGGGTTTGGGAACTGACTAATCCCCCTAATGATTGGTGCCCCTGATATAGCCTTTCCCCGAATAAACAACATAAGCTTTTGACTCTTACCACCATCTTTCCTACTTCTACTTGCATCCTCTGGCGTTGAAGCTGGAGCTGGTACCGGGTGGACTGTTTACCCACCGCTTGCTAGCAACCTAGCACACGCTGGGGCATCTGTAGACCTAACTATTTTCTCCCTCCACCTAGCAGGTATTTCGTCCATCCTAGGTGCTATCAATTTTATTACCACTATTCTAAATATGAAGCCCCCAGCAATTTCTCAATACCAGACGCCCCTATTTGTATGAGCAGTACTAATTACAGCCGTGCTTCTTCTTCTATCACTCCCTGTGCTTGCCGCTGGCATCACTATGCTTCTAACAGATCGCAACCTAAACACATCCTTCTTTGACCCTGCAGGGGGTGGTGACCCGATTCTTTACCAACACCTTTTC

>KY275338.1 *Synechogobius ommaturus*

TTGGTGCCTGAGCCGGAATAGTAGGCACAGCTCTAAGCCTTTTAATTCGGGCTGAACTGAGCCAGCCTGGGGCTCTTTTAGGGGATGACCAGATTTACAATGTCATCGTGACAGCACATGCATTTGTAATAATTTTCTTTATAGTAATACCAATCATAATTGGAGGGTTTGGGAACTGACTAATCCCCCTAATGATTGGTGCCCCTGATATAGCCTTTCCCCGAATAAACAACATAAGCTTTTGACTCTTACCACCATCTTTCCTACTTTTACTTGCATCCTCTGGCGTTGAAGCTGGAGCTGGTACCGGGTGGACTGTTTACCCACCGCTTGCTAGCAACCTAGCACACGCTGGGGCATCTGTAGACCTAACTATTTTCTCCCTCCACCTAGCAGGTATTTCGTCCATCCTAGGTGCTATCAATTTTATTACCACTATTCTAAATATGAAGCCCCCAGCAATTTCTCAATACCAGACGCCCCTATTTGTATGAGCAGTACTAATTACAGCCGTGCTTCTTCTTCTATCACTCCCTGTGCTTGCCGCTGGCATCACTATGCTTCTAACAGATCGCAACCTAAACACATCCTTCTTTGACCCTGCAGGGGGTGGTGACCCGATTCTTTACCAACACCTTTTC

>KY275339.1 *Synechogobius ommaturus*

TTGGTGCCTGAGCCGGAATAGTAGGCACAGCTCTAAGCCTTTTAATTCGGGCTGAACTGAGCCAGCCTGGGGCTCTTTTAGGGGATGACCAGATTTACAATGTCATCGTGACAGCACATGCATTTGTAATAATTTTCTTTATAGTAATACCAATCATAATTGGAGGGTTTGGGAACTGACTAATCCCCCTAATGATTGGTGCCCCTGATATAGCCTTTCCCCGAATAAACAACATAAGCTTTTGACTCTTACCACCATCTTTCCTACTTCTACTTGCATCCTCTGGCGTTGAAGCTGGAGCTGGTACCGGGTGGACTGTTTACCCACCGCTTGCTAGCAACCTAGCACACGCTGGGGCATCTGTAGACCTAACTATTTTCTCCCTCCACCTAGCAGGTATTTCGTCCATCCTAGGTGCTATCAATTTTATTACCACTATTCTAAATATGAAGCCCCCAGCAATTTCTCAATACCAGACGCCCCTATTTGTATGAGCAGTACTAATTACAGCCGTGCTTCTTCTTCTATCACTCCCTGTGCTTGCCGCTGGCATCACTATGCTTCTAACAGATCGCAACCTAAACACATCCTTCTTTGACCCTGCAGGGGGTGGTGACCCGATTCTTTACCAACACCTTTTC

>KY275340.1 *Synechogobius ommaturus*

TTGGTGCCTGAGCCGGAATAGTAGGCACAGCTCTAAGCCTTTTAATTCGGGCTGAACTGAGCCAGCCTGGGGCTCTTTTAGGGGATGACCAGATTTACAATGTCATCGTGACAGCACATGCATTTGTAATAATTTTCTTTATAGTAATACCAATCATAATTGGAGGGTTTGGGAACTGACTAATCCCCCTAATGATTGGTGCCCCTGATATAGCCTTTCCCCGAATAAACAACATAAGCTTTTGACTCTTACCACCATCTTTCCTACTTCTACTTGCATCCTCTGGCGTTGAAGCTGGAGCTGGTACCGGGTGGACTGTTTACCCACCGCTTGCTAGCAACCTAGCACACGCTGGGGCATCTGTAGACCTAACTATTTTCTCCCTCCACCTAGCAGGTATTTCGTCCATCCTAGGTGCTATCAATTTTATTACCACTATTCTAAATATGAAGCCCCCAGCAATTTCTCAATACCAGACGCCCCTATTTGTATGAGCAGTACTAATTACAGCCGTGCTTCTTCTTCTATCACTCCCTGTGCTTGCCGCTGGCATCACTATGCTTCTAACAGATCGCAACCTAAACACATCCTTCTTTGACCCTGCAGGGGGTGGTGACCCGATTCTTTACCAACACCTTTTC

>KY275341.1 *Synechogobius ommaturus*

TTGGTGCCTGAGCCGGAATAGTAGGCACAGCTCTAAGCCTTTTAATTCGGGCTGAACTGAGCCAGCCTGGGGCTCTTTTAGGGGATGACCAGATTTACAATGTCATCGTGACAGCACATGCATTTGTAATAATTTTCTTTATAGTAATACCAATCATAATTGGAGGGTTTGGGAACTGACTAATCCCCCTAATGATTGGTGCCCCTGATATAGCCTTTCCCCGAATAAACAACATAAGCTTTTGACTCTTACCACCATCTTTCCTACTTCTACTTGCATCCTCTGGCGTTGAAGCTGGAGCTGGTACCGGGTGGACTGTTTACCCACCGCTTGCTAGCAACCTAGCACACGCTGGGGCATCTGTAGACCTAACTATTTTCTCCCTCCACCTAGCAGGTATTTCGTCCATCCTAGGTGCTATCAATTTTATTACCACTATTCTAAATATGAAGCCCCCAGCAATTTCTCAATACCAGACGCCCCTATTTGTATGAGCAGTACTAATTACAGCCGTGCTTCTTCTTCTATCACTCCCTGTGCTTGCCGCTGGCATCACTATGCTTCTAACAGATCGCAACCTAAACACATCCTTCTTTGACCCTGCAGGGGGTGGTGACCCGATTCTTTACCAACACCTTTTC

>KY275342.1 *Synechogobius ommaturus*

TTGGTGCCTGAGCCGGAATAGTAGGCACAGCTCTAAGCCTTTTAATTCGGGCTGAACTGAGCCAGCCTGGGGCTCTTTTAGGGGATGACCAGATTTACAATGTCATCGTGACAGCACATGCATTTGTAATAATTTTCTTTATAGTAATACCAATCATAATTGGAGGGTTTGGGAACTGACTAATCCCCCTAATGATTGGTGCCCCTGATATAGCCTTTCCCCGAATAAACAACATAAGCTTTTGACTCTTACCACCATCTTTCCTACTTCTACTTGCATCCTCTGGCGTTGAAGCTGGAGCTGGTACCGGGTGGACTGTTTACCCACCGCTTGCTAGCAACCTAGCACACGCTGGGGCATCTGTAGACCTAACTATTTTCTCCCTCCACCTAGCAGGTATTTCGTCCATCCTAGGTGCTATCAATTTTATTACCACTATTCTAAATATGAAGCCCCCAGCAATTTCTCAATACCAGACGCCCCTATTTGTATGAGCAGTACTAATTACAGCCGTGCTTCTTCTTCTATCACTCCCTGTGCTTGCCGCTGGCATCACTATGCTTCTAACAGATCGCAACCTAAACACATCCTTCTTTGACCCTGCAGGGGGTGGTGACCCGATTCTTTACCAACACCTTTTC

>KY275343.1 *Acanthogobius flavimanus*

CTGAGCCGGAATAGTAGGCACAGCTCTGAGCCTCTTAATTCGGGCTGAGCTAAGCCAGCCTGGGGCTCTTTTGGGAGATGACCAGATTTACAATGTTATCGTAACCGCACATGCATTTGTAATAATCTTCTTTATAGTAATACCAATTATAATTGGGGGATTTGGGAACTGACTTATTCCGCTAATGATCGGCGCCCCCGACATGGCTTTCCCCCGAATAAACAACATGAGCTTTTGACTTCTACCACCGTCATTCCTCCTTCTCCTGGCATCCTCTGGTGTCGAAGCCGGGGCAGGAACGGGATGGACTGTGTACCCCCCGCTCGCCAGCAACCTCGCCCATGCTGGGGCATCCGTAGACCTAACAATCTTCTCTCTTCACCTGGCAGGAATTTCATCCATTCTAGGTGCCATTAATTTTATTACTACAATCCTAAATATGAAACCCCCAGCAATTTCTCAGTACCAAACACCTCTATTTGTATGGGCAGTTTTAATTACAGCCGTACTACTTCTCCTATCCCTCCCAGTACTTGCCGCCGGCATTACAATGCTTCTAACAGACCGCAACTTAAACACATCATTCTTTGACCCTGCAGGGGGTGGTGACCCAATTCTTTACCAACACCTCTTC

>KY275344.1 *Acanthogobius flavimanus*

CTGAGCCGGAATAGTAGGCACAGCTCTGAGCCTCTTAATTCGGGCTGAGCTAAGCCAGCCTGGGGCTCTTTTGGGAGATGACCAGATTTACAATGTTATCGTAACCGCACATGCATTTGTAATAATCTTCTTTATAGTAATACCAATTATAATTGGGGGATTTGGGAACTGACTTATTCCGCTAATGATCGGCGCCCCCGACATGGCTTTCCCCCGAATAAACAACATGAGCTTTTGACTTCTACCACCGTCATTCCTCCTTCTCCTGGCATCCTCTGGTGTCGAAGCCGGGGCAGGAACGGGATGGACTGTGTACCCCCCGCTCGCCAGCAACCTCGCCCATGCTGGGGCATCCGTAGACCTAACAATCTTCTCTCTTCACCTGGCAGGAATTTCATCCATTCTAGGTGCCATTAATTTTATTACTACAATCCTAAATATGAAACCCCCAGCAATTTCTCAGTACCAAACACCTCTATTTGTATGGGCAGTTTTAATTACAGCCGTACTACTTCTCCTATCCCTCCCAGTACTTGCCGCCGGCATTACAATGCTTCTAACAGACCGCAACTTAAACACATCATTCTTTGACCCTGCAGGGGGTGGTGACCCAATTCTTTACCAACACCTCTTC

>KY275345.1 *Acanthogobius flavimanus*

CTGAGCCGGAATAGTAGGCACAGCTCTGAGCCTCTTAATTCGGGCTGAGCTAAGCCAGCCTGGGGCTCTTTTGGGAGATGACCAGATTTACAATGTTATCGTAACCGCACATGCATTTGTAATAATCTTCTTTATAGTAATACCAATTATAATTGGGGGATTTGGGAACTGACTTATTCCGCTAATGATCGGCGCCCCCGACATGGCTTTCCCCCGAATAAACAACATGAGCTTTTGACTTCTACCACCGTCATTCCTCCTTCTCCTGGCATCCTCTGGTGTCGAAGCCGGGGCAGGAACGGGATGGACTGTGTACCCCCCGCTCGCCAGCAACCTCGCCCATGCTGGGGCATCCGTAGACCTAACAATCTTCTCTCTTCACCTGGCAGGAATTTCATCCATTCTAGGTGCCATTAATTTTATTACTACAATCCTAAATATGAAACCCCCAGCAATTTCTCAGTACCAAACACCTCTATTTGTATGGGCAGTTTTAATTACAGCCGTACTACTTCTCCTATCCCTCCCAGTACTTGCCGCCGGCATTACAATGCTTCTAACAGACCGCAACTTAAACACATCATTCTTTGACCCTGCAGGGGGTGGTGACCCAATTCTTTACCAACACCTCTTC

>KY275346.1 *Acanthogobius flavimanus*

CTGAGCCGGAATAGTAGGCACAGCTCTGAGCCTCTTAATTCGGGCTGAGCTAAGCCAGCCTGGGGCTCTTTTGGGAGATGACCAGATTTACAATGTTATCGTAACCGCACATGCATTTGTAATAATCTTCTTTATAGTAATACCAATTATAATTGGGGGATTTGGGAACTGACTTATTCCGCTAATGATCGGCGCCCCCGACATGGCTTTCCCCCGAATAAACAACATGAGCTTTTGACTTCTACCACCGTCATTCCTCCTTCTCCTGGCATCCTCTGGTGTCGAAGCCGGGGCAGGAACGGGATGGACTGTGTACCCCCCGCTCGCCAGCAACCTCGCCCATGCTGGGGCATCCGTAGACCTAACAATCTTCTCTCTTCACCTGGCAGGAATTTCATCCATTCTAGGTGCCATTAATTTTATTACTACAATCCTAAATATGAAACCCCCAGCAATTTCTCAGTACCAAACACCTCTATTTGTATGGGCAGTTTTAATTACAGCCGTACTACTTCTCCTATCCCTCCCAGTACTTGCCGCCGGCATTACAATGCTTCTAACAGACCGCAACTTAAACACATCATTCTTTGACCCTGCAGGGGGTGGTGACCCAATTCTTTACCAACACCTCTTC

>KY275347.1 *Acanthogobius flavimanus*

CTGAGCCGGAATAGTAGGCACAGCTCTGAGCCTCTTAATTCGGGCTGAGCTAAGCCAGCCTGGGGCTCTTTTGGGAGATGACCAGATTTACAATGTTATCGTAACCGCACATGCATTTGTAATAATCTTCTTTATAGTAATACCAATTATAATTGGGGGATTTGGGAACTGACTTATTCCGCTAATGATCGGCGCCCCCGACATGGCTTTCCCCCGAATAAACAACATGAGCTTTTGACTTCTACCACCGTCATTCCTCCTTCTCCTGGCATCCTCTGGTGTCGAAGCCGGGGCAGGAACGGGATGGACTGTGTACCCCCCGCTCGCCAGCAACCTCGCCCATGCTGGGGCATCCGTAGACCTAACAATCTTCTCTCTTCACCTGGCAGGAATTTCATCCATTCTAGGTGCCATTAATTTTATTACTACAATCCTAAATATGAAACCCCCAGCAATTTCTCAGTACCAAACACCTCTATTTGTATGGGCAGTTTTAATTACAGCCGTACTACTTCTCCTATCCCTCCCAGTACTTGCCGCCGGCATTACAATGCTTCTAACAGACCGCAACTTAAACACATCATTCTTTGACCCTGCAGGGGGTGGTGACCCAATTCTTTACCAACACCTCTTC

>KY275348.1 *Acanthogobius flavimanus*

CTGAGCCGGAATAGTAGGCACAGCTCTGAGCCTCTTAATTCGGGCTGAGCTAAGCCAGCCTGGGGCTCTTTTGGGAGATGACCAGATTTACAATGTTATCGTAACCGCACATGCATTTGTAATAATCTTCTTTATAGTAATACCAATTATAATTGGGGGATTTGGGAACTGACTTATTCCGCTAATGATCGGCGCCCCCGACATGGCTTTCCCCCGAATAAACAACATGAGCTTTTGACTTCTACCACCGTCATTCCTCCTTCTCCTGGCATCCTCTGGTGTCGAAGCCGGGGCAGGAACGGGATGGACTGTGTACCCCCCGCTCGCCAGCAACCTCGCCCATGCTGGGGCATCCGTAGACCTAACAATCTTCTCTCTTCACCTGGCAGGAATTTCATCCATTCTAGGTGCCATTAATTTTATTACTACAATCCTAAATATGAAACCCCCAGCAATTTCTCAGTACCAAACACCTCTATTTGTATGGGCAGTTTTAATTACAGCCGTACTACTTCTCCTATCCCTCCCAGTACTTGCCGCCGGCATTACAATGCTTCTAACAGACCGCAACTTAAACACATCATTCTTTGACCCTGCAGGGGGTGGTGACCCAATTCTTTACCAACACCTCTTC

>KY275349.1 *Pterogobius zacalles*

GTAGGCACAGCTTTAAGCCTATTGATCCGAGCAGAACTAAGTCAACCTGGAGCCCTTCTAGGAGACGACCAAATTTATAATGTTATTGTGACAGCCCATGCCTTTGTTATAATTTTCTTTATAGTAATACCCATCATAATTGGAGGCTTTGGAAATTGACTTATTCCCCTAATAATTGGAGCCCCCGACATAGCTTTTCCTCGAATGAATAACATAAGCTTCTGGCTCCTTCCTCCTTCCTTTCTTCTTCTTCTTGCTTCCTCTGGTGTTGAAGCTGGGGCCGGAACAGGATGAACTGTTTACCCTCCCTTAGCTGGTAATCTTGCTCATGCTGGAGCCTCTGTTGATCTTACAATTTTCTCCCTTCATTTAGCAGGGATTTCCTCTATTTTAGGCGCTATTAACTTTATTACAACAATTATGAATATGAAGCCCCCAGCTATTTCACAATATCAAACCCCACTATTTGTCTGAGCTGTCCTAATTACAGCTGTTCTTCTACTTCTTTCCCTCCCCGTACTTGCCGCTGGAATTACAATACTTCTTACAGATCGAAATCTTAATACAACTTTCTTTGACCCCGCAGGAGGAGGAGATCCTATTCTTTATCAACATCTT

>KY275350.1 *Pterogobius zacalles*

GTAGGCACAGCTTTAAGCCTATTGATCCGAGCAGAACTAAGTCAACCTGGAGCCCTTCTAGGAGACGACCAAATTTATAATGTTATTGTGACAGCCCATGCCTTTGTTATAATTTTCTTTATAGTAATACCCATCATAATTGGAGGCTTTGGAAATTGACTTATTCCCCTAATAATTGGAGCCCCCGACATAGCTTTTCCTCGAATGAATAACATAAGCTTCTGGCTCCTTCCTCCTTCCTTTCTTCTTCTTCTTGCTTCCTCTGGTGTTGAAGCTGGGGCCGGAACAGGATGAACTGTTTACCCTCCCTTAGCTGGTAATCTTGCTCATGCTGGAGCCTCTGTTGATCTTACAATTTTCTCCCTTCATTTAGCAGGGATTTCCTCTATTTTAGGCGCTATTAACTTTATTACAACAATTATGAATATGAAGCCCCCAGCTATTTCACAATATCAAACCCCACTATTTGTCTGAGCTGTCCTAATTACAGCTGTTCTTCTACTTCTTTCCCTCCCCGTACTTGCCGCTGGAATTACAATACTTCTTACAGATCGAAATCTTAATACAACTTTCTTTGACCCCGCAGGAGGAGGAGATCCTATTCTTTATCAACATCTT

>KY275351.1 *Pterogobius zacalles*

GTAGGCACAGCTTTAAGCCTATTGATCCGAGCAGAACTAAGTCAACCTGGAGCCCTTCTAGGAGACGACCAAATTTATAATGTTATTGTGACAGCCCATGCCTTTGTTATAATTTTCTTTATAGTAATACCCATCATAATTGGAGGCTTTGGAAATTGACTTATTCCCCTAATAATTGGAGCCCCCGACATAGCTTTTCCTCGAATGAATAACATAAGCTTCTGGCTCCTTCCTCCTTCCTTTCTTCTTCTTCTTGCTTCCTCTGGTGTTGAAGCTGGGGCCGGAACAGGATGAACTGTTTACCCTCCCTTAGCTGGTAATCTTGCTCATGCTGGAGCCTCTGTTGATCTTACAATTTTCTCCCTTCATTTAGCAGGGATTTCCTCTATTTTAGGCGCTATTAACTTTATTACAACAATTATGAATATGAAGCCCCCAGCTATTTCACAATATCAAACCCCACTATTTGTCTGAGCTGTCCTAATTACAGCTGTTCTTCTACTTCTTTCCCTCCCCGTACTTGCCGCTGGAATTACAATACTTCTTACAGATCGAAATCTTAATACAACTTTCTTTGACCCCGCAGGAGGAGGAGATCCTATTCTTTATCAACATCTT

>KY275352.1 *Pterogobius zacalles*

GTAGGCACAGCTTTAAGCCTATTGATCCGAGCAGAACTAAGTCAACCTGGAGCCCTTCTAGGAGACGACCAAATTTATAATGTTATTGTGACAGCCCATGCCTTTGTTATAATTTTCTTTATAGTAATACCCATCATAATTGGAGGCTTTGGAAATTGACTTATTCCCCTAATAATTGGAGCCCCCGACATAGCTTTTCCTCGAATGAATAACATAAGCTTCTGGCTCCTTCCTCCTTCCTTTCTTCTTCTTCTTGCTTCCTCTGGTGTTGAAGCTGGGGCCGGAACAGGATGAACTGTTTACCCTCCCTTAGCTGGTAATCTTGCTCATGCTGGAGCCTCTGTTGATCTTACAATTTTCTCCCTTCATTTAGCAGGGATTTCCTCTATTTTAGGCGCTATTAACTTTATTACAACAATTATGAATATGAAGCCCCCAGCTATTTCACAATATCAAACCCCACTATTTGTCTGAGCTGTCCTAATTACAGCTGTTCTTCTACTTCTTTCCCTCCCCGTACTTGCCGCTGGAATTACAATACTTCTTACAGATCGAAATCTTAATACAACTTTCTTTGACCCCGCAGGAGGAGGAGATCCTATTCTTTATCAACATCTT

>KY275353.1 *Pterogobius zacalles*

GTAGGCACAGCTTTAAGCCTATTGATCCGAGCAGAACTAAGTCAACCTGGAGCCCTTCTAGGAGACGACCAAATTTATAATGTTATTGTGACAGCCCATGCCTTTGTTATAATTTTCTTTATAGTAATACCCATCATAATTGGAGGCTTTGGAAATTGACTTATTCCCCTAATAATTGGAGCCCCCGACATAGCTTTTCCTCGAATGAATAACATAAGCTTCTGGCTCCTTCCTCCTTCCTTTCTTCTTCTTCTTGCTTCCTCTGGTGTTGAAGCTGGGGCCGGAACAGGATGAACTGTTTACCCTCCCTTAGCTGGTAATCTTGCTCATGCTGGAGCCTCTGTTGATCTTACAATTTTCTCCCTTCATTTAGCAGGGATTTCCTCTATTTTAGGCGCTATTAACTTTATTACAACAATTATGAATATGAAGCCCCCAGCTATTTCACAATATCAAACCCCACTATTTGTCTGAGCTGTCCTAATTACAGCTGTTCTTCTACTTCTTTCCCTCCCCGTACTTGCCGCTGGAATTACAATACTTCTTACAGATCGAAATCTTAATACAACTTTCTTTGACCCCGCAGGAGGAGGAGATCCTATTCTTTATCAACATCTT

>KU236857.1 *Larimichthys polyactis*

GAGCCGGAATAGTGGGCACCGGCCTAAGTCTCATTATTCGAGCAGAGCTAAGCCAGCCCGGCTCGCTTCTCGGAGACGACCAGATTTTTAACGTAGTTGTTACGGCACATGCCTTCGTTATAATCTTCTTTATAGTAATACCCGTAATAATCGGAGGGTTCGGAAACTGACTCGTGCCTTTAATAATTGGCGCCCCCGACATAGCATTTCCCCGAATAAATAACATAAGCTTCTGACTTATCCCCCCTGCTTTCATTATGCTCGCAGCCTCATCAGCGGTTGAAGCAGGGGCCGGAACAGGGTGAACAGTCTACCCCCCACTTGCTGGAAATCTCGCACACGCAGGAGCTTCAGTCGACTTATCCATTTTCGCTCTGCACCTTGCGGGTGTCTCTTCAATCCTGGGGGTCATCAACTTCATCACAACGATTCTTAACATAAAACCCCCCGGCATAACCCACTACCAAACACCCCTGTTTGTGTGATCCGTTCTGATTACAGCATACCTCCTCCTACTATCACTGACCGTCCTAGCTGCCGGCATCACAATGCTTTTAACAGACCGCAACCTCAACACAACCTTTTTTGACCCCTCAAGTGGAGGCGATCCCATCCTTTATCAACAACTATTC

>KU236858.1 *Pennahia argentata*

AGCCGGAATAGTAGGCACAGCCCTGAGTCTTCTAATCCGGGCAGAACTAAGCCAACCCGGTTCCCTTCTCGGGGACGATCAAATTTATAACGTCATCGTCACAGCCCATGCCTTTGTCATGATTTTCTTTATAGTAATGCCCGTTATGATCGGAGGTTTTGGGAACTGACTTATCCCCTTAATAATCGGTGCCCCCGACATAGCATTCCCCCGAATAAACAATATGAGTTTCTGACTTCTTCCCCCTTCCTTCCTTCTTCTCCTAACTTCTTCAGGTGTTGAAGCGGGAGCTGGAACAGGATGAACAGTCTACCCCCCACTCGCTGGAAACCTCGCACATGCAGGAGCCTCCGTCGACTTGGCCATCTTCTCCCTACACCTCGCAGGTGTCTCTTCTATTCTGGGGGCTATCAACTTTATTACAACAATTATCAACATAAAACCCCCTGCCATTTCTCAGTATCAGACACCCTTATTTGTGTGGGCCGTCCTGATTACAGCAGTTCTACTACTACTATCACTACCCGTGCTAGCTGCTGGCATTACAATACTTTTAACTGATCGTAACCTAAACACAACCTTCTTCGACCCGGCAGGCGGGGGAGATCCAATTCTTTACCAGCACTTATTC

>KU236859.1 *Pennahia argentata*

AGCCGGAATAGTAGGCACAGCCCTGAGTCTTCTAATCCGGGCAGAACTAAGCCAACCCGGCTCCCTTCTCGGGGACGATCAAATTTATAACGTCATCGTCACAGCCCATGCCTTTGTCATGATTTTCTTTATAGTAATGCCCGTTATGATCGGAGGTTTTGGGAACTGACTTATCCCCTTAATAATCGGTGCCCCCGACATAGCATTCCCCCGAATAAACAATATGAGTTTCTGACTTCTTCCCCCTTCCTTCCTTCTTCTCCTAACTTCTTCAGGTGTTGAAGCGGGAGCTGGAACAGGATGAACAGTCTACCCCCCACTCGCTGGAAACCTCGCACATGCAGGAGCCTCCGTCGACTTGGCCATCTTCTCCCTACACCTCGCAGGTGTCTCTTCTATCCTGGGGGCTATCAACTTTATTACAACAATTATCAACATAAAACCCCCTGCCATTTCTCAGTATCAGACACCCTTATTTGTGTGGGCCGTCCTGATTACAGCAGTTCTACTACTACTATCCCTACCCGTGCTAGCTGCTGGCATTACAATACTTTTAACTGATCGTAACCTAAACACAACCTTCTTCGACCCGGCAGGCGGGGGAGATCCAATTCTTTACCAACACTTATTC

>KU236860.1 *Pennahia argentata*

AGCCGGAATAGTAGGCACAGCCCTGAGTCTTCTAATCCGGGCAGAACTAAGCCAACCCGGTTCCCTTCTCGGGGACGATCAAATTTATAACGTCATCGTCACAGCCCATGCCTTTGTCATGATTTTCTTTATAGTAATGCCCGTTATGATCGGAGGTTTTGGGAACTGACTTATCCCCTTAATAATCGGTGCCCCCGACATAGCATTCCCCCGAATAAACAATATGAGTTTCTGACTTCTTCCCCCTTCCTTCCTTCTTCTCCTAACTTCTTCAGGTGTTGAAGCGGGAGCTGGAACAGGATGAACAGTCTACCCCCCACTCGCTGGAAACCTCGCACATGCAGGAGCCTCCGTCGACTTGGCCATCTTCTCCCTACACCTCGCAGGTGTCTCTTCTATTCTGGGGGCTATCAACTTTATTACAACAATTATCAACATAAAACCCCCTGCCATTTCTCAGTATCAGACACCCTTATTTGTGTGGGCCGTCCTGATTACAGCAGTTCTACTACTACTATCACTACCCGTGCTAGCTGCTGGCATTACAATACTTTTAACTGATCGTAACCTAAACACAACCTTCTTCGACCCGGCAGGCGGGGGAGATCCAATTCTTTACCAGCACTTATTC

>KU236861.1 *Pennahia argentata*

AGCCGGAATAGTAGGCACAGCCCTGAGTCTTCTAATCCGGGCAGAACTAAGCCAACCCGGTTCCCTTCTCGGGGACGATCAAATTTATAACGTCATCGTCACAGCCCATGCCTTTGTCATGATTTTCTTTATAGTAATGCCCGTTATGATTGGAGGTTTTGGGAACTGACTTATCCCCTTAATAATCGGTGCCCCCGACATAGCATTCCCCCGAATAAACAATATGAGTTTCTGACTTCTTCCCCCTTCCTTCCTTCTTCTCCTAACTTCTTCAGGTGTTGAAGCGGGAGCTGGAACAGGATGAACAGTCTACCCCCCACTCGCTGGAAACCTCGCACATGCAGGAGCCTCCGTCGACTTGGCCATCTTCTCCCTACACCTCGCAGGTGTCTCTTCTATTCTGGGGGCTATCAACTTTATTACAACAATTATCAACATAAAACCCCCTGCCATTTCTCAGTATCAGACACCCTTATTTGTGTGGGCCGTCCTGATTACAGCAGTTCTACTACTACTATCACTACCAGTGCTAGCTGCTGGCATTACAATACTTTTAACTGATCGTAACCTAAACACAACCTTCTTCGACCCGGCAGGCGGGGGAGATCCAATTCTTTACCAGCACTTATTC

>KU236862.1 *Pennahia argentata*

AGCCGGAATAGTAGGCACAGCCCTGAGTCTTCTAATCCGGGCAGAACTAAGCCAACCCGGTTCCCTTCTCGGGGACGATCAAATTTATAACGTCATCGTCACAGCCCATGCCTTTGTCATGATTTTCTTTATAGTAATGCCCGTTATGATCGGAGGTTTTGGGAACTGACTTATCCCCTTAATAATCGGTGCCCCCGACATAGCATTCCCCCGAATAAACAATATGAGTTTCTGACTTCTTCCCCCTTCCTTCCTTCTTCTCCTAACTTCTTCAGGTGTTGAAGCGGGAGCTGGAACAGGATGAACAGTCTACCCCCCACTCGCTGGAAACCTCGCACATGCAGGAGCCTCCGTCGACTTGGCCATCTTCTCCCTACACCTCGCAGGTGTCTCTTCTATTCTGGGGGCTATCAACTTTATTACAACAATTATCAACATAAAACCCCCTGCCATTTCTCAGTATCAGACACCCTTATTTGTGTGGGCCGTCCTGATTACAGCAGTTCTACTACTACTATCACTACCCGTGCTAGCTGCTGGCATTACAATACTTTTAACTGATCGTAACCTAAACACAACCTTCTTCGACCCGGCAGGCGGGGGAGATCCAATTCTTTACCAGCACTTATTC

>KU236863.1 *Sillago japonica*

TTTCGGTGCCTGAGCAGGCATGGTCGGTACGGCCTTAAGCCTGCTGATCCGAGCGGAACTCAGCCAACCTGGCGCCCTACTCGGAGATGACCAAATCTACAACGTAATTGTTACGGCACATGCCTTTGTAATGATTTTCTTTATAGTTATACCCATCCTAATTGGAGGCTTTGGAAACTGGCTAGTCCCTTTAATAATTGGAGCCCCTGACATGGCCTTCCCGCGAATGAATAATATGAGCTTCTGGCTTCTACCGCCTTCCTTCCTCCTTCTATTAGCCTCCTCTGGGGTTGAAGCTGGGGCCGGAACCGGTTGAACAGTTTACCCTCCTTTGGCAGGGAATTTAGCCCACGCAGGGGCTTCTGTTGATTTAACTATCTTTTCTCTTCACTTGGCAGGGATTTCATCGATTTTAGGGGCAATTAACTTCATTACAACTATCATCAACATAAAACCTCCAGCAACTTCACAATATCAAACCCCCCTATTCGTATGATCTGTTCTAATTACAGCCGTTCTTCTACTCCTCTCGCTCCCAGTACTTGCCGCTGGAATTACTATGCTTCTAACGGATCGAAACCTAAACACCACGTTCTTTGACCCTGCTGGGGGTGGTGACCCAATTCTTTACCAACACCTCTTC

>KU236864.1 *Apogon lineatus*

AGCTGGAATAGTTGGAACAGCTCTTAGCTTACTCATCCGGGCTGAACTAAGCCAACCCGGGGCCCTTCTTGGCGACGACCAAATTTATAACGTTATCGTTACGGCGCATGCATTTGTAATAATCTTCTTTATAGTAATACCAATCATGATTGGAGGCTTCGGAAACTGACTTATCCCCCTAATGATTGGGGCCCCTGATATAGCATTTCCTCGAATGAATAACATAAGCTTCTGACTCCTTCCCCCCTCTTTCCTACTGCTACTTGCCTCGTCGGGCGTTGAAGCCGGGGCAGGAACAGGATGAACGGTTTACCCACCTCTTGCAGGCAACCTTGCCCACGCAGGGGCCTCTGTAGATTTAACAATTTTTTCTCTACATCTTGCAGGAATTTCCTCAATTCTAGGGGCTATTAACTTCATTACAACAATTGTTAATATAAAACCTCCCGCTATTACTCAGTACCAAACTCCCCTATTTGTTTGAGCTGTCCTAATCACTGCCGTCCTTCTTCTCCTCTCTCTTCCTGTTCTAGCCGCAGGCATTACAATGCTACTCACTGATCGGAACTTAAATACAACCTTCTTTGACCCGGCAGGAGGAGGTGACCCAATTCTTTACCAACACCTA

>KU236865.1 *Apogon lineatus*

AGCTGGAATAGTTGGAACAGCTCTTAGCTTACTCATCCGGGCTGAACTAAGCCAACCCGGGGCCCTTCTTGGCGACGACCAAATTTATAACGTTATCGTTACGGCGCATGCATTTGTAATAATCTTCTTTATAGTAATACCAATCATGATTGGAGGCTTCGGAAACTGACTTATCCCCCTAATGATTGGGGCCCCTGATATAGCATTTCCTCGAATGAATAACATAAGCTTCTGACTCCTTCCCCCCTCTTTCCTACTGCTACTTGCCTCGTCGGGCGTTGAAGCCGGGGCAGGAACAGGATGAACGGTTTACCCACCTCTTGCAGGCAACCTTGCCCACGCAGGGGCCTCTGTAGATTTAACAATTTTTTCTCTACATCTTGCAGGAATTTCCTCAATTCTAGGGGCTATTAACTTCATTACAACAATTGTTAATATAAAACCTCCCGCTATTACTCAGTACCAAACTCCCCTATTTGTTTGAGCTGTCCTAATCACTGCCGTCCTTCTTCTCCTCTCTCTTCCTGTTCTAGCCGCAGGCATTACAATGCTACTCACTGATCGGAACTTAAATACAACCTTCTTTGACCCGGCAGGAGGAGGTGACCCAATTCTTTACCAACACCTA

>KU236866.1 *Apogon lineatus*

AGCTGGAATAGTTGGAACAGCTCTTAGCTTACTCATCCGGGCTGAACTAAGCCAACCCGGGGCCCTTCTTGGCGACGACCAAATTTATAACGTTATCGTTACGGCGCATGCATTTGTAATAATCTTCTTTATAGTAATACCAATCATGATTGGAGGCTTCGGAAACTGACTTATCCCCCTAATGATTGGGGCCCCTGATATAGCATTTCCTCGAATGAATAACATAAGCTTCTGACTCCTTCCCCCCTCTTTCCTACTGCTACTTGCCTCGTCGGGCGTTGAAGCCGGGGCAGGAACAGGATGAACGGTTTACCCACCTCTTGCAGGCAACCTTGCCCACGCAGGGGCCTCTGTAGATTTAACAATTTTTTCTCTACATCTTGCAGGAATTTCCTCAATTCTAGGGGCTATTAACTTCATTACAACAATTGTTAATATAAAACCTCCCGCTATTACTCAGTACCAAACTCCCCTATTTGTTTGAGCTGTCCTAATCACTGCCGTCCTTCTTCTCCTCTCTCTTCCTGTTCTAGCCGCAGGCATTACAATGCTACTCACTGATCGGAACTTAAATACAACCTTCTTTGACCCGGCAGGAGGAGGTGACCCAATTCTTTACCAACACCTA

>KU236867.1 *Acanthopagrus schlegelii*

TGGTGCTTGAGCTGGAATAGTAGGAACCGCCTTAAGTCTGCTCATTCGAGCCGAATTAAGCCAACCTGGCGCTCTCCTAGGAGATGATCAAATTTATAATGTAATTGTTACAGCACATGCGTTTGTAATAATTTTCTTTATAGTAATACCAATTATGATTGGGGGCTTTGGAAATTGATTAGTACCACTTATGATTGGTGCCCCTGACATAGCATTCCCCCGTATAAACAACATAAGCTTCTGACTTCTTCCTCCATCATTCCTTCTGCTGCTAGCTTCTTCTGGTGTCGAAGCTGGGGCCGGTACCGGGTGGACAGTTTACCCCCCACTGGCAGGAAACCTCGCCCACGCAGGTGCATCAGTTGACTTAACCATCTTTTCTCTTCACCTAGCCGGAATTTCATCTATTCTTGGGGCCATCAATTTTATTACCACTATTATTAATATGAAACCGCCAGCTATCTCACAATATCAAACACCCCTATTTGTGTGGGCCGTTTTAATTACTGCTGTCCTACTCCTCTTGTCCCTCCCAGTTCTTGCTGCCGGAATTACAATACTCCTTACAGACCGAAATCTAAATACCACCTTCTTTGACCCAGCTGGAGGAGGAGACCCTATTCTCTATCAACACCTATTC

>KY275354.1 *Acanthopagrus schlegelii*

GTAGGAACCGCCTTAAGTCTGCTCATTCGAGCCGAATTAAGCCAACCTGGCGCTCTCCTAGGAGATGATCAAATTTATAATGTAATTGTTACAGCACATGCGTTTGTAATAATTTTCTTTATAGTAATACCAATTATGATTGGGGGCTTTGGAAATTGATTAGTACCACTTATGATTGGTGCCCCTGACATAGCATTCCCCCGTATAAACAACATAAGCTTCTGACTTCTTCCTCCATCATTCCTTCTGCTGCTAGCTTCTTCTGGTGTCGAAGCTGGGGCCGGTACCGGGTGGACAGTTTACCCCCCACTGGCAGGAAACCTCGCCCACGCAGGTGCATCAGTTGACTTAACCATCTTTTCTCTTCACCTAGCCGGAATTTCATCTATTCTTGGGGCCATCAATTTTATTACCACTATTATTAATATGAAACCGCCAGCTATCTCACAATATCAAACACCCCTATTTGTGTGGGCCGTTTTAATTACTGCTGTCCTACTCCTCTTGTCCCTCCCAGTTCTTGCTGCCGGAATTACAATACTCCTTACAGACCGAAATCTAAATACCACCTTCTTTGACCCAGCTGGAGGAGGAGACCCTATTCTCTATCAACACCT

>KU236868.1 *Scomber japonicus*

CCTCTACCTAGTATTCGGTGCATGAGCTGGAATAGTTGGCACGGCCTTAAGCTTGCTTATCCGAGCTGAACTAAGTCAACCAGGGTCCCTTCTCGGCGACGACCAAATCTACAACGTAATTGTTACGGCCCACGCCTTCGTTATAATCTTCTTTTTAGTAATGCCAGTTATGATTGGAGGGTTCGGAAACTGACTGATTCCCCTAATGATCGGAGCCCCCGACATGGCATTTCCCCGAATAAATAACATAAGCTTCTGACTTCTGCCCCCCTCTCTCCTGCTGCTCCTGTCTTCTTCGGCAGTTGAAGCCGGTGCCGGAACTGGCTGAACAGTTTATCCTCCCCTCGCTGGGAACCTGGCACACGCCGGGGCATCAGTTGATTTGACCATCTTCTCACTCCACCTAGCAGGTGTTTCCTCAATCCTTGGGGCCATTAACTTCATCACAACAATCATTAACATAAAACCTGCAGGTGTGTCCCAATACCAAACCCCTCTGTTCGTCTGAGCAGTCCTAATTACAGCTGTCCTTCTCCTTCTATCCCTACCAGTTCTTGCTGCCGGCATTACAATGCTCCTAACAGACCGAAATCTAAATACTACCTTCTTCGACCCTGGAGGAGGGGGAGACCCCAT

>KU236869.1 *Saurida elongata*

TTTGGTGCATGGGCCGGCATGGTAGGTACTGCCCTTAGCCTTCTAATCCGTGCTGAACTGAGCCAACCAGGCGCCCTTCTGGGGGACGACCAGATCTATAACGTAATTGTTACCGCACACGCCTTTGTAATAATTTTCTTTATAGTAATACCAATCATGATTGGCGGATTTGGAAACTGGCTTATTCCCCTCATAATTGGTGCCCCCGACATGGCGTTTCCCCGTATGAACAACATGAGCTTTTGACTCCTTCCCCCTTCCTTCCTACTACTTCTTGCCTCCTCCGGTGTTGAGGCCGGGGCTGGGACCGGATGAACGGTCTACCCACCCCTAGCAGGCAATCTCGCCCATGCCGGGGCATCCGTTGATCTAACTATCTTTTCGCTTCACTTGGCAGGGATCTCCTCTATCCTGGGGGCCATTAACTTTATTACCACAATTGTTAACATGAAACCCCCCGCTATTTCGCAGTACCAAACCCCACTGTTTGTCTGAGCAGTCCTAATTACCGCCGTTCTCCTTCTCCTCTCCCTCCCTGTTCTCGCAGCTGGAATTACAATACTTCTTACAGACCGGAACCTCAACACTACCTTCTTCGACCCTGCAGGAGGGGGAGATCCAATTCTTTATCAACACTTGTTT

>KY275355.1 *Lateolabrax japonicus*

ATAGTGGGGACGGCCTTAAGCCTACTCATTCGAGCAGAACTAAGTCAACCAGGTGCCTTGTTAGGAAGCGACCAGATCTACAACGTCATCGTTACAGCACACGCGTTCGTGATAATCTTCTTTATAGTAATACCAATTATGATTGGAGGGTTTGGAAACTGATTAATTCCCCTAATGATCGGCGCCCCAGATATAGCGTTCCCTCGGATGAACAACATAAGCTTTTGACTCCTTCCCCCCTCCCTCCTTCTCCTCCTCTCCTCTTCTGCAGTAGAAGCCGGGGCCGGAACTGGGTGAACCGTTTACCCTCCCTTAGCCAGCAACTTAGCTCACGCAGGGGCCTCCGTCGATCTAACAATCTTCTCCTTACACCTAGCAGGGGTTTCTTCAATCCTGGGGGCTATTAACTTTATCACAACCATCATTAACATGAAACCGCCCGCCATTTCCCAGTATCAAACCCCCCTATTTGTGTGAGCCGTTTTAATCACGGCCGTCCTCCTTCTCCTCTCCCTCCCCGTTCTCGCTGCAGGCATTACAATGCTTCTCACAGATCGAAACCTTAACACCACTTTCTTCGATCCCGCCGGAGGCGGGGACCCGATCCTCTATCAACACCTATTC

>KY275356.1 *Lateolabrax japonicus*

ATAGTGGGGACGGCCTTAAGCCTACTCATTCGAGCAGAACTAAGTCAACCGGGTGCCTTGTTAGGAAGCGACCAGATCTACAACGTCATCGTTACAGCACACGCGTTCGTGATAATCTTCTTTATAGTAATACCAATTATGATTGGGGGGTTTGGAAACTGATTAATTCCCCTAATGATCGGCGCCCCAGATATAGCGTTCCCTCGGATGAACAACATAAGCTTTTGACTCCTTCCCCCCTCCCTCCTTCTCCTCCTCTCCTCTTCTGCAGTAGAAGCCGGGGCCGGAACTGGGTGAACCGTTTACCCTCCCTTAGCCAGCAACTTAGCTCACGCAGGGGCCTCCGTCGATCTAACAATCTTCTCCTTACACCTAGCAGGGGTTTCTTCAATCCTGGGGGCTATTAACTTTATCACAACCATCATTAACATGAAACCGCCCGCCATTTCCCAGTATCAAACCCCCCTATTTGTGTGAGCCGTTTTAATCACGGCCGTCCTCCTTCTCCTCTCCCTCCCCGTTCTCGCTGCAGGCATTACAGTGCTTCTCACAGATCGAAACCTTAACACCACTTTCTTCGACCCCGCCGGAGGCGGGGACCCGATCCTCTATCAACACCTATTC

>KY275357.1 *Lateolabrax japonicus*

ATAGTGGGGACGGCCTTAAGCCTACTCATTCGAGCAGAACTAAGTCAACCAGGTGCCTTGTTAGGAAGCGACCAGATCTACAACGTCATCGTTACAGCACACGCGTTCGTGATAATCTTCTTTATAGTAATACCAATTATGATTGGAGGGTTTGGAAACTGATTAATTCCCCTAATGATCGGCGCCCCAGATATAGCGTTCCCTCGGATGAACAACATAAGCTTTTGACTCCTTCCCCCCTCCCTCCTTCTCCTCCTCTCCTCTTCTGCAGTAGAAGCCGGGGCCGGAACTGGGTGAACCGTTTACCCTCCCTTAGCCAGCAACTTAGCTCACGCAGGGGCCTCCGTCGATCTAACAATCTTCTCCTTACACCTAGCAGGGGTTTCTTCAATCCTGGGGGCTATTAACTTTATCACAACCATCATTAACATGAAACCGCCCGCCATTTCCCAGTATCAAACCCCCCTATTTGTGTGAGCCGTTTTAATCACGGCCGTCCTCCTTCTCCTCTCCCTCCCCGTTCTCGCTGCAGGCATTACAATGCTTCTCACAGATCGAAACCTTAACACCACTTTCTTCGATCCCGCCGGAGGCGGGGACCCGATCCTCTATCAACACCTATTC

>KU236870.1 *Conger myriaster*

CCTTTATTTAGTATTTGGTGCTTGAGCCGGCATAGTAGGAACCGCTTTAAGTCTGCTAATTCGAGCTGAATTAAGTCAACCTGGAGCTCTCCTTGGAGATGACCAGATCTATAATGTTATCGTAACAGCACATGCCTTTGTAATAATTTTCTTTATAGTAATACCAGTTATAATTGGTGGATTCGGCAATTGACTTGTGCCACTAATAATTGGGGCCCCAGACATGGCATTCCCTCGAATAAACAACATAAGCTTCTGATTATTACCACCATCATTTCTTTTATTATTAACCTCATCTGGAGTTGAAGCAGGGGCCGGAACAGGATGAACTGTTTATCCCCCACTATCAGGGAACCTGGCCCACGCTGGGGCATCAGTGGACCTAACAATCTTTTCTCTACACCTAGCAGGTGTCTCATCCATCCTGGGGGCCATTAACTTTATTACTACTATTATTAATATAAAACCACCAGCCACTACACAATATCAAACCCCCCTATTTGTATGGTCTGTTTTAATCACTGCCGTTCTACTACTTTTATCACTCCCTGTTCTTGCTGCGGGTATTACAATGCTTTTAACAGATCGAAATCTTAATACCACCTTCTTTGACCCAGCTGGGGGAGGAGACCCAATTCTTTACCAACACCTAT

>KU236871.1 *Conger myriaster*

CCTTTATTTAGTATTTGGTGCTTGAGCCGGCATAGTAGGAACCGCTTTAAGTCTGCTAATTCGAGCTGAATTAAGTCAACCTGGAGCTCTCCTTGGAGATGACCAGATCTATAATGTTATCGTAACAGCACATGCCTTTGTAATAATTTTCTTTATAGTAATACCAGTTATAATTGGTGGATTCGGCAATTGACTTGTGCCACTAATAATTGGGGCCCCAGACATGGCATTCCCTCGAATAAACAACATAAGCTTCTGATTATTACCACCATCATTTCTTTTATTATTAACCTCATCTGGAGTTGAAGCAGGGGCCGGAACAGGATGAACTGTTTATCCCCCACTATCAGGGAACCTGGCCCACGCTGGGGCATCAGTGGACCTAACAATCTTTTCTCTACACCTAGCAGGTGTCTCATCCATCCTGGGGGCCATTAACTTTATTACTACTATTATTAATATAAAACCACCAGCCACTACACAATATCAAACCCCCCTATTTGTATGGTCTGTTTTAATCACTGCCGTTCTACTACTTTTATCACTCCCTGTTCTTGCTGCGGGTATTACAATGCTTTTAACAGATCGAAATCTTAATACCACCTTCTTTGACCCAGCTGGGGGAGGAGACCCAATTCTTTACCAACACCTAT

>KU236872.1 *Conger myriaster*

CCTTTATTTAGTATTTGGTGCTTGAGCCGGCATAGTAGGAACCGCTTTAAGTCTGCTAATTCGAGCTGAATTAAGTCAACCTGGAGCTCTCCTTGGAGATGACCAGATCTATAATGTTATCGTAACAGCACATGCCTTTGTAATAATTTTCTTTATAGTAATACCAGTTATAATTGGTGGATTCGGCAATTGACTTGTGCCACTAATAATTGGGGCCCCAGACATGGCATTCCCTCGAATAAACAACATAAGCTTCTGATTATTACCACCATCATTTCTTTTATTATTAACCTCATCTGGAGTTGAAGCAGGGGCCGGAACAGGATGAACTGTTTATCCCCCACTATCAGGGAACCTGGCCCACGCTGGGGCATCAGTAGACCTAACAATCTTTTCTCTACACCTAGCAGGTGTCTCATCCATCCTGGGGGCCATTAACTTTATTACTACTATTATTAATATAAAACCACCAGCCACTACACAATATCAAACCCCCCTATTTGTATGGTCTGTTTTAATCACTGCCGTTCTACTACTTTTATCACTCCCTGTTCTTGCTGCGGGTATTACAATGCTTTTAACAGATCGAAATCTTAATACCACCTTCTTTGACCCAGCTGGGGGAGGAGACCCAATTCTTTACCAACACCTAT

>KU236873.1 *Conger myriaster*

CCTTTATTTAGTATTTGGTGCTTGAGCCGGCATAGTAGGAACCGCTTTAAGTCTGCTAATTCGAGCTGAATTAAGTCAACCTGGAGCTCTCCTTGGAGATGACCAGATCTATAATGTTATCGTAACAGCACATGCCTTTGTAATAATTTTCTTTATAGTAATACCAGTTATAATTGGTGGATTCGGCAATTGACTTGTGCCACTAATAATTGGGGCCCCAGACATGGCATTCCCTCGAATAAACAACATAAGCTTCTGATTATTACCACCATCATTTCTTTTATTATTAACCTCATCTGGAGTTGAAGCAGGGGCCGGAACAGGATGAACTGTTTATCCCCCACTATCAGGGAACCTGGCCCACGCTGGGGCATCAGTGGACCTAACAATCTTTTCTCTACACCTAGCAGGTGTCTCATCCATCCTGGGGGCCATTAACTTTATTACTACTATTATTAATATAAAACCACCAGCCACTACACAATATCAAACCCCCCTATTTGTATGGTCTGTTTTAATCACTGCCGTTCTACTACTTTTATCACTCCCTGTTCTTGCTGCGGGTATTACAATGCTTTTAACAGATCGAAATCTTAATACCACCTTCTTTGACCCAGCTGGGGGAGGAGACCCAATTCTTTACCAACACCTAT

>KU236874.1 *Conger myriaster*

CCTTTATTTAGTATTTGGTGCTTGAGCCGGCATAGTAGGAACCGCTTTAAGTCTGCTAATTCGAGCTGAATTAAGTCAACCTGGAGCTCTCCTTGGAGATGACCAGATCTATAATGTTATCGTAACAGCACATGCCTTTGTAATAATTTTCTTTATAGTAATACCAGTTATAATTGGTGGATTCGGCAATTGACTTGTGCCACTAATAATTGGGGCCCCAGACATGGCATTCCCTCGAATAAACAACATAAGCTTCTGATTATTACCACCATCATTTCTTTTATTATTAACCTCATCTGGAGTTGAAGCAGGGGCCGGAACAGGATGAACTGTTTATCCCCCACTATCAGGGAACCTGGCCCACGCTGGGGCATCAGTGGACCTAACAATCTTTTCTCTACACCTAGCAGGTGTCTCATCCATCCTGGGGGCCATTAACTTTATTACTACTATTATTAATATAAAACCACCAGCCACTACACAATATCAAACCCCCCTATTTGTATGGTCTGTTTTAATCACTGCCGTTCTACTACTTTTATCACTCCCTGTTCTTGCTGCGGGTATTACAATGCTTTTAACAGATCGAAATCTTAATACCACCTTCTTTGACCCAGCTGGGGGAGGAGACCCAATTCTTTACCAACACCTAT

> KY275358.1 *Conger myriaster*

CCTTTATTTAGTATTTGGTGCTTGAGCCGGCATAGTAGGAACCGCTTTAAGTCTGCTAATTCGAGCTGAATTAAGTCAACCTGGAGCTCTCCTTGGAGATGACCAGATCTATAATGTTATCGTAACAGCACATGCCTTTGTAATAATTTTCTTTATAGTAATACCAGTTATAATTGGTGGATTCGGCAATTGACTTGTGCCACTAATAATTGGGGCCCCAGACATGGCATTCCCTCGAATAAACAACATAAGCTTCTGATTATTACCACCATCATTTCTTTTATTATTAACCTCATCTGGAGTTGAAGCAGGGGCCGGAACAGGATGAACTGTTTATCCCCCACTATCAGGGAACCTGGCCCACGCTGGGGCATCAGTGGACCTAACAATCTTTTCTCTACACCTAGCAGGTGTCTCATCCATCCTGGGGGCCATTAACTTTATTACTACTATTATTAATATAAAACCACCAGCCACTACACAATATCAAACCCCCCTATTTGTATGGTCTGTTTTAATCACTGCCGTTCTACTACTTTTATCACTCCCTGTTCTTGCTGCGGGTATTACAATGCTTTTAACAGATCGAAATCTTAATACCACCTTCTTTGACCCAGCTGGGGGAGGAGACCCAATTCTTTACCAACACCTAT

> KY275359.1 *Conger myriaster*

CCTTTATTTAGTATTTGGTGCTTGAGCCGGCATAGTAGGAACCGCTTTAAGTCTGCTAATTCGAGCTGAATTAAGTCAACCTGGAGCTCTCCTTGGAGATGACCAGATCTATAATGTTATCGTAACAGCACATGCCTTTGTAATAATTTTCTTTATAGTAATACCAGTTATAATTGGTGGATTCGGCAATTGACTTGTGCCACTAATAATTGGGGCCCCAGACATGGCATTCCCTCGAATAAACAACATAAGCTTCTGATTATTACCACCATCATTTCTTTTATTATTAACCTCATCTGGAGTTGAAGCAGGGGCCGGAACAGGATGAACTGTTTATCCCCCACTATCAGGGAACCTGGCCCACGCTGGGGCATCAGTAGACCTAACAATCTTTTCTCTACACCTAGCAGGTGTCTCATCCATCCTGGGGGCCATTAACTTTATTACTACTATTATTAATATAAAACCACCAGCCACTACACAATATCAAACCCCCCTATTTGTATGGTCTGTTTTAATCACTGCCGTTCTACTACTTTTATCACTCCCTGTTCTTGCTGCGGGTATTACAATGCTTTTAACAGATCGAAATCTTAATACCACCTTCTTTGACCCAGCTGGGGGAGGAGACCCAATTCTTTACCAACACCTAT

> KY275360.1 *Conger myriaster*

CCTTTATTTAGTATTTGGTGCTTGAGCCGGCATAGTAGGAACCGCTTTAAGTCTGCTAATTCGAGCTGAATTAAGTCAACCTGGAGCTCTCCTTGGAGATGACCAGATCTATAATGTTATCGTAACAGCACATGCCTTTGTAATAATTTTCTTTATAGTAATACCAGTTATAATTGGTGGATTCGGCAATTGACTTGTGCCACTAATAATTGGGGCCCCAGACATGGCATTCCCTCGAATAAACAACATAAGCTTCTGATTATTACCACCATCATTTCTTTTATTATTAACCTCATCTGGAGTTGAAGCAGGGGCCGGAACAGGATGAACTGTTTATCCCCCACTATCAGGGAACCTGGCCCACGCTGGGGCATCAGTGGACCTAACAATCTTTTCTCTACACCTAGCAGGTGTCTCATCCATCCTGGGGGCCATTAACTTTATTACTACTATTATTAATATAAAACCACCAGCCACTACACAATATCAAACCCCCCTATTTGTATGGTCTGTTTTAATCACTGCCGTTCTACTACTTTTATCACTCCCTGTTCTTGCTGCGGGTATTACAATGCTTTTAACAGATCGAAATCTTAATACCACCTTCTTTGACCCAGCTGGGGGAGGAGACCCAATTCTTTACCAACACCTAT

> KY275361.1 *Conger myriaster*

CCTTTATTTAGTATTTGGTGCTTGAGCCGGCATAGTAGGAACCGCTTTAAGTCTGCTAATTCGAGCTGAATTAAGTCAACCTGGAGCTCTCCTTGGAGATGACCAGATCTATAATGTTATCGTAACAGCACATGCCTTTGTAATAATTTTCTTTATAGTAATACCAGTTATAATTGGTGGATTCGGCAATTGACTTGTGCCACTAATAATTGGGGCCCCAGACATGGCATTCCCTCGAATAAACAACATAAGCTTCTGATTATTACCACCATCATTTCTTTTATTATTAACCTCATCTGGAGTTGAAGCAGGGGCCGGAACAGGATGAACTGTTTATCCCCCACTATCAGGGAACCTGGCCCACGCTGGGGCATCAGTGGACCTAACAATCTTTTCTCTACACCTAGCAGGTGTCTCATCCATCCTGGGGGCCATTAACTTTATTACTACTATTATTAATATAAAACCACCAGCCACTACACAATATCAAACCCCCCTATTTGTATGGTCTGTTTTAATCACTGCCGTTCTACTACTTTTATCACTCCCTGTTCTTGCTGCGGGTATTACAATGCTTTTAACAGATCGAAATCTTAATACCACCTTCTTTGACCCAGCTGGGGGAGGAGACCCAATTCTTTACCAACACCTAT

>KY275362.1 *Conger myriaster*

CCTTTATTTAGTATTTGGTGCTTGAGCCGGCATAGTAGGAACCGCTTTAAGTCTGCTAATTCGAGCTGAATTAAGTCAACCTGGAGCTCTCCTTGGAGATGACCAGATCTATAATGTTATCGTAACAGCACATGCCTTTGTAATAATTTTCTTTATAGTAATACCAGTTATAATTGGTGGATTCGGCAATTGACTTGTGCCACTAATAATTGGGGCCCCAGACATGGCATTCCCTCGAATAAACAACATAAGCTTCTGATTATTACCACCATCATTTCTTTTATTATTAACCTCATCTGGAGTTGAAGCAGGGGCCGGAACAGGATGAACTGTTTATCCCCCACTATCAGGGAACCTGGCCCACGCTGGGGCATCAGTGGACCTAACAATCTTTTCTCTACACCTAGCAGGTGTCTCATCCATCCTGGGGGCCATTAACTTTATTACTACTATTATTAATATAAAACCACCAGCCACTACACAATATCAAACCCCCCTATTTGTATGGTCTGTTTTAATCACTGCCGTTCTACTACTTTTATCACTCCCTGTTCTTGCTGCGGGTATTACAATGCTTTTAACAGATCGAAATCTTAATACCACCTTCTTTGACCCAGCTGGGGGAGGAGACCCAATTCTTTACCAACACCTAT

>KU236875.1 *Neosalanx anderssoni*

CCTATATCTAATCTTCGGAGCCTGGGCAGGAATAGTGGGGACAGCCCTTAGCCTCCTCATCCGGGCCGAACTTAGCCAACCCGGCGCCCTCCTTGGAGACGACCAGATCTATAACGTCATCGTTACTGCACACGCCTTCGTAATAATCTTCTTTATAGTTATACCCATCCTGATCGGAGGGTTCGGAAACTGACTTGTTCCCCTTATAATCGGGGCCCCCGATATAGCATTTCCCCGAATAAACAACATAAGCTTCTGACTCTTACCCCCTTCTTTCCTGCTTCTACTAGCCTCCTCCGGGGTCGAAGCTGGGGCTGGTACTGGGTGAACCGTATACCCCCCTCTTTCCGGGAACCTCGCCCACGCCGGGGCCTCCGTAGACCTTACGATCTTCTCTCTCCACCTTGCCGGGATCTCTTCCATCCTCGGGGCAATTAACTTCATCACGACCATCATCAACATAAAACCCCCTGCCACTTCTCAGTACCAAACACCCCTGTTCGTCTGATCTGTCCTCATTACTGCCGTCCTATTGCTGTTGTCTCTGCCCGTCCTAGCTGCTGGCATCACCATGCTTCTGACAGATCGAAACCTAAACACCACCTTCTTCGACCCAGCAGGCGGAGGAGACCCCATCTTGTACCAACACCTGTTC

>KU236876.1 *Lophius litulon*

GGTGCCTGAGCCGGAATAGTGGGCACCGCCCTAAGCTTACTAATTCGGGCTGAACTAAGCCAACCCGGCGCCCTCTTAGGGGATGACCAAATCTACAACGTTATTGTTACCGCACATGCCTTTGTAATAATTTTCTTTATGGTTATACCAATTATGATCGGAGGATTCGGCAATTGACTTATCCCCCTAATGATCGGAGCCCCAGACATGGCTTTCCCCCGAATGAATAACATAAGCTTCTGGCTTCTCCCCCCCTCTTTCCTCCTACTACTTGCCTCTTCCGGGGTTGAAGCCGGAGCAGGCACTGGATGAACCGTCTACCCCCCGCTGGCAGGAAACCTTGCACATGCAGGGGCTTCCGTAGACCTAACGATTTTTTCCCTTCATCTAGCCGGGATCTCTTCAATCCTAGGGGCAATCAACTTTATTACAACAATTATTAATATAAAACCCCCCACAATCTCCCAGTACCAGACGCCTTTATTCGTATGGGCTGTTTTAATCACAGCAGTTCTATTACTCCTGTCCCTACCCGTGCTTGCGGCAGGAATTACTATACTCTTAACAGACCGAAACCTAAACACCACTTTTTTTGATCCCACGGGAGGAGGGGACCCTATCCTGTACCAACACTTA

>KU236877.1 *Lophius litulon*

GGTGCCTGAGCCGGAATAGTGGGCACCGCCCTAAGCTTACTAATTCGGGCTGAACTAAGCCAACCCGGCGCCCTCTTAGGGGATGACCAAATCTACAACGTTATTGTTACCGCACATGCCTTTGTAATAATTTTCTTTATGGTTATACCAATTATGATCGGAGGATTCGGCAATTGACTTATCCCCCTAATGATCGGAGCCCCAGACATGGCTTTCCCCCGAATGAATAACATAAGCTTCTGGCTTCTCCCCCCCTCTTTCCTCCTACTACTTGCCTCTTCCGGGGTTGAAGCCGGAGCAGGCACTGGATGAACCGTCTACCCCCCGCTGGCAGGAAACCTTGCACATGCAGGGGCTTCCGTAGACCTAACGATTTTTTCCCTTCATCTAGCCGGGATCTCTTCAATCCTAGGGGCAATCAACTTTATTACAACAATTATTAATATAAAACCCCCCACAATCTCCCAGTACCAGACGCCTTTATTCGTATGGGCTGTTTTAATCACAGCAGTTCTATTACTCCTGTCCCTACCCGTGCTTGCGGCAGGAATTACTATACTCTTAACAGACCGAAACCTAAACACCACTTTTTTTGATCCCACGGGAGGAGGGGACCCTATCCTGTACCAACACTTA

>KU236878.1 *Thamnaconus modestus*

GGAATAGTAGGAACTGCTTTGAGCCTACTGATTCGAGCAGAACTAAGCCAACCCGGCGCCCTCCTTGGAGATGACCAGATTTATAACGTAATCGTAACAGCTCACGCTTTTGTAATGATTTTCTTTATAGTAATGCCAATTATAATTGGAGGTTTCGGAAACTGACTTATCCCTCTAATGATCGGTGCCCCTGATATAGCATTCCCTCGAATGAACAATATGAGCTTCTGATTACTTCCCCCTTCCTTCCTCCTTCTCCTCGCGTCTTCAGGGGTTGAAGCTGGGGCCGGAACCGGATGGACCGTTTACCCCCCTCTGGCAGGAAACCTAGCCCACGCTGGGGCATCCGTAGACCTCACAATTTTCTCCCTCCACTTAGCAGGTATTTCTTCAATTCTTGGTGCAATTAATTTTATCACAACTATCATCAACATGAAACCTCCCGCCATTTCCCAATACCAAACGCCCCTATTTGTTTGAGCTGTACTAATTACAGCCGTACTTCTTCTTCTCTCCCTGCCTGTACTTGCTGCAGGAATCACGATGCTCCTGACTGACCGTAATTTAAACACCACCTTCTTCGACCCAGCTGGAGGGGGAGACCC

>KY275363.1 *Takifugu niphobles*

CCTATACCTAGTTTTTGGTGCCTGAGCCGGAATAGTAGGCACAGCACTAAGTCTTCTTATTCGGGCCGAACTCAGTCAACCCGGTGCACTCTTGGGCGATGACCAGATCTACAATGTAATCGTTACAGCCCATGCATTCGTAATGATTTTCTTTATAGTAATACCAATCATGATTGGAGGCTTTGGGAACTGATTAATTCCCCTTATAATCGGAGCCCCAGACATGGCCTTCCCCCGAATAAACAACATAAGCTTCTGACTGCTTCCCCCATCCTTCCTCCTTCTGCTCGCATCCTCTGGAGTAGAAGCCGGAGCGGGTACAGGCTGAACCGTTTACCCACCCCTAGCAGGAAATCTTGCCCACGCAGGAGCTTCTGTAGACCTTACCATCTTCTCTCTTCATCTTGCAGGGGTCTCCTCTATTCTAGGGGCAATCAACTTCATCACAACTATCATTAACATGAAACCCCCAGCAATCTCACAATACCAAACACCTCTTTTCGTATGAGCCGTTTTAATTACTGCTGTACTTCTCCTGCTCTCCCTTCCTGTCCTTGCAGCAGGAATTACAATGCTTCTCACTGACCGAAACTTAAATACAACCTTCTTTGACCCAGCAGGAGGAGGAGACCCCATCCTGTACCAACACTTATTC

>KU236879.1 *Konosirus punctatus*

AGCAGGAATAGTAGGGACTGCCCTAAGCCTCCTAATCCGAGCGGAACTTAGCCAGCCCGGCGCGCTCCTAGGAGACGATCAAATCTACAATGTTATCGTTACGGCACACGCCTTTGTAATGATTTTCTTCATAGTAATGCCAATCCTGATTGGAGGGTTTGGGAACTGATTGGTTCCCCTAATGATCGGGGCACCCGACATGGCATTCCCGCGAATGAATAACATGAGCTTCTGACTTCTTCCTCCCTCTTTCCTTCTCCTCTTGGCCTCCTCCGGTGTAGAAGCTGGGGCGGGGACAGGATGGACAGTCTACCCCCCTTTATCAGGGAACCTAGCCCATGCAGGTGCATCCGTCGACCTAACCATCTTCTCTCTCCATCTTGCAGGTATTTCATCGATCCTAGGGGCAATCAATTTTATTACCACAATTATTAATATGAAACCCCCTGCAATCTCGCAATACCAAACTCCTTTATTCGTTTGGGCCGTGCTTGTCACTGCTGTATTACTTCTGCTATCTCTTCCGGTGCTGGCTGCGGGAATCACTATGCTTCTAACGGACCGGAATCTTAATACCACCTTCTTCGATCCTGCTGGCGGAGGAGACCCAATCCTTTATCAACACCTC

>KU236880.1 *Konosirus punctatus*

AGCAGGAATAGTAGGGACTGCCCTAAGCCTCCTAATCCGAGCGGAACTTAGCCAGCCCGGCGCGCTCCTAGGAGACGATCAAATCTACAATGTTATCGTTACGGCACACGCCTTTGTAATGATTTTCTTCATAGTAATGCCAATCCTGATTGGAGGGTTTGGGAACTGATTGGTTCCCCTAATGATCGGGGCACCCGACATGGCATTCCCGCGAATGAATAACATGAGCTTCTGACTTCTTCCTCCCTCTTTCCTTCTCCTCTTGGCCTCCTCCGGTGTAGAAGCTGGGGCGGGGACAGGATGGACAGTCTACCCCCCTTTATCAGGGAACCTAGCCCATGCAGGTGCATCCGTCGACCTAACCATCTTCTCTCTCCATCTTGCAGGTATTTCATCGATCCTAGGGGCAATCAATTTTATTACCACAATTATTAATATGAAACCCCCTGCAATCTCGCAATACCAAACTCCTTTATTCGTTTGGGCCGTGCTTGTCACTGCTGTATTACTTCTGCTATCTCTTCCGGTGCTGGCTGCGGGAATCACTATGCTTCTAACGGACCGGAATCTTAATACCACCTTCTTCGATCCTGCTGGCGGAGGAGACCCAATCCTTTATCAACACCTC

>KU236881.1 *Engraulis japonicus*

TATATTATGTGCGGTACCTGAGCAGGAATGGTAGGGACAGCATTTTTTTTCTTTATTCGAGCAGAACTAAGCCAACCACGAGCACTTGTGGGGGACGATCATATTTATAACGTAATCGTTCCTGCTCACGCATTCGTAATAATCTTGTTTATGGTAATGCCCATCCTAATCGGTGGGTTCGGGAATTGACTGGTTCCTCTTATACTAGGGGCCCCAGACATGGCATTCCCCCGAATGAACAATATGAGCTTTTGACTCCTTCCCCCTTCTTTCCTTCTCCTCTTAGCATCATCTGGTGTTGAAGCAGGGGCCGGGACAGGATGAACAGTCTACCCCCCTCTAGCAGGAAACCTCGCCCACGCCGGAGCGTCAGTAGATTTAACAATCTTCTCTCTCCACCTGGCAGGGATTTCATCAATCCTAGGTGCCATTAATTTCATTACTACCATCATTAATATGAAACCACCTGCTATTTCACAATACCAGACACCTCTATTTGTCTGAGCTGTATTAATCACGGCAGTACTTTTACTTCTTTCACTACCCGTTCTAGCTGCTGGGATTACTATGCTTCTTACAGACCGAAACCTAAATACTACTTTCTTCGACCCAGCGGGGGGAGGAGAC

>KU236882.1 *Thryssa kammalensis*

GGTGCCTGGGCAGGAATAGTAGGAACAGCACTTAGCCTTTTAATTCGGGCAGAACTAAGCCAGCCCGGAGCACTCCTAGGGGACGACCAAATTTATAATGTTATTGTTACTGCTCATGCATTCGTAATAATTTTCTTCATGGTTATACCAATTTTAATTGGTGGATTCGGAAACTGATTAGTACCGCTTATACTAGGCGCGCCTGATATAGCCTTTCCCCGAATAAATAACATAAGCTTCTGACTTCTACCACCCTCTTTTCTTCTTTTACTTGCCTCCTCAGGAGTTGAGGCAGGGGCAGGAACTGGATGAACAGTTTATCCCCCACTAGCAGGAAACCTGGCCCACGCAGGAGCCTCAGTAGACCTAACTATTTTTTCACTACACTTAGCTGGTATTTCATCTATTCTCGGGGCCATTAATTTTATTACTACTATTATTAACATAAAACCACCTGCAATCTCACAATACCAAACACCTCTGTTCGTCTGAGCTGTGCTGATTACAGCAGTACTTTTACTTCTATCTCTGCCAGTCCTTGCGGCTGGCATTACAATGCTTCTTACAGACCGAAACCTAAACACCACTTTCTTTGACCCAGCAGGAGGAGGGGACCCTATTCTTTACCAACACCTA

>KU236883.1 *Thryssa kammalensis*

GGTGCCTGGGCAGGAATAGTAGGAACAGCACTTAGCCTTTTAATTCGGGCAGAACTAAGCCAGCCCGGAGCACTCCTAGGGGACGACCAAATTTATAATGTTATTGTTACTGCTCATGCATTCGTAATAATTTTCTTCATGGTTATACCAATTTTAATTGGTGGATTCGGAAACTGATTAGTACCGCTTATACTAGGCGCGCCTGATATAGCCTTTCCCCGAATAAATAACATAAGCTTCTGACTTCTACCACCCTCTTTTCTTCTTTTACTTGCCTCCTCAGGAGTTGAGGCAGGGGCAGGAACTGGATGAACAGTTTATCCCCCACTAGCAGGAAACCTGGCCCACGCAGGAGCCTCAGTAGACCTAACTATTTTTTCACTACACTTAGCTGGTATTTCATCTATTCTCGGGGCCATTAATTTTATTACTACTATTATTAACATAAAACCACCTGCAATCTCACAATACCAAACACCTCTGTTCGTCTGAGCTGTGCTGATTACAGCAGTACTTTTACTTCTATCTCTGCCAGTCCTTGCGGCTGGCATTACAATGCTTCTTACAGACCGAAACCTAAACACCACTTTCTTTGACCCAGCAGGAGGAGGGGACCCCATTCTTTACCAACACCTA

>KU236884.1 *Thryssa kammalensis*

GGTGCCTGGGCAGGAATAGTAGGAACAGCACTTAGCCTTTTAATTCGGGCAGAACTAAGCCAGCCCGGAGCACTCCTAGGGGACGACCAAATTTATAATGTTATTGTTACTGCTCATGCATTCGTAATAATTTTCTTCATGGTTATACCAATTTTAATTGGTGGATTCGGAAACTGATTAGTACCGCTTATACTAGGCGCGCCTGATATAGCCTTTCCCCGAATAAATAACATAAGCTTCTGACTTCTACCACCCTCTTTTCTTCTTTTACTTGCCTCCTCAGGAGTTGAGGCAGGGGCAGGAACTGGATGAACAGTTTATCCCCCACTAGCAGGAAACCTGGCCCACGCAGGAGCCTCAGTAGACCTAACTATTTTTTCACTACACTTAGCTGGTATTTCATCTATTCTCGGGGCCATTAATTTTATTACTACTATTATTAACATAAAACCACCTGCAATCTCACAATACCAAACACCTCTGTTCGTCTGAGCTGTGCTGATTACAGCAGTACTTTTACTTCTATCTCTGCCAGTCCTTGCGGCTGGCATTACAATGCTTCTTACAGACCGAAACCTAAACACCACTTTCTTTGACCCAGCAGGAGGAGGGGACCCTATTCTTTACCAACACCTA

>KU236885.1 *Thryssa kammalensis*

GGTGCCTGGGCAGGAATAGTAGGAACAGCACTTAGCCTTTTAATTCGGGCAGAACTAAGCCAGCCCGGAGCACTCCTAGGGGACGACCAAATTTATAATGTTATTGTTACTGCTCATGCATTCGTAATAATTTTCTTCATGGTTATACCAATTTTAATTGGTGGATTCGGAAACTGATTAGTACCGCTTATACTAGGCGCGCCTGATATAGCCTTTCCCCGAATAAATAACATAAGCTTCTGACTTCTACCACCCTCTTTTCTTCTTTTACTTGCCTCCTCAGGAGTTGAGGCAGGGGCAGGAACTGGATGAACAGTTTATCCCCCACTAGCAGGAAACCTGGCCCACGCAGGAGCCTCAGTAGACCTAACTATTTTTTCACTACACTTAGCTGGTATTTCATCTATTCTCGGGGCCATTAATTTTATTACTACTATTATTAATATAAAACCACCTGCAATCTCACAATACCAAACACCTCTGTTCGTCTGAGCTGTGCTGATTACAGCAGTACTTTTACTTCTATCTCTGCCAGTCCTTGCGGCTGGCATTACAATGCTTCTTACAGACCGAAACCTAAACACCACTTTCTTTGACCCAGCAGGAGGAGGGGACCCTATTCTTTACCAACACCTA

>KU236886.1 *Thryssa kammalensis*

GGTGCCTGGGCAGGAATAGTAGGAACAGCACTTAGCCTTTTAATTCGGGCAGAACTAAGCCAGCCCGGAGCACTCCTAGGGGACGACCAAATTTATAATGTTATTGTTACTGCTCATGCATTCGTAATAATTTTCTTCATGGTTATACCAATTTTAATTGGTGGATTCGGAAACTGATTAGTACCGCTTATACTAGGCGCGCCTGATATAACCTTTCCCCGAATAAATAACATAAGCTTCTGACTTCTACCACCCTCTTTTCTTCTTTTACTTGCCTCCTCAGGAGTTGAGGCAGGGGCAGGAACTGGATGAACAGTTTATCCCCCGCTAGCAGGAAACCTGGCCCACGCAGGAGCCTCAGTAGACCTAACTATTTTTTCACTACACTTAGCTGGTATTTCATCTATTCTCGGGGCCATTAATTTTATTACTACTATTATTAACATAAAACCACCTGCAATCTCACAATACCAAACACCTCTGTTCGTCTGAGCTGTGCTGATTACAGCAGTACTTTTACTTCTATCTCTGCCAGTCCTTGCGGCTGGCATTACAATGCTTCTTACAGACCGAAACCTAAACACCACTTTCTTTGACCCAGCAGGAGGAGGGGACCCTATTCTTTACCAACACCTA

>KU236887.1 *Okamejei kenojei*

TGAGCAGGGATGGTTGGGACTGGCCTAAGCCTCTTAATCCGAGCAGAACTGAGCCAACCGGGGACCCTCCTAGGTGATGATCAGATTTATAATGTCATTGTTACAGCCCACGCCTTAGTAATAATCTTTTTTATGGTTATACCAATTATAATCGGCGGGTTCGGCAACTGACTTGTCCCCTTAATGATCGGCTCCCCAGACATAGCCTTCCCACGCATAAATAATATAAGTTTTTGGCTCCTGCCCCCATCTTTTCTTCTCCTCCTGGCCTCAGCCGGAGTCGAGGCCGGGGCCGGGACAGGTTGAACCGTCTACCCCCCACTAGCAGGGAATCTAGCCCACGCCGGGGCCTCCGTAGACCTGACAATTTTCTCCCTTCACTTGGCAGGCATCTCATCTATCCTAGCCTCCATTAACTTCATCACCACAATTATCAATATAAAACCACCAGCAATCTCCCAATACCAAACACCATTATTCGTATGATCAATTCTTGTTACAACTGTCTTACTTCTTATAGCCCTGCCAGTTCTAGCAGCTGGCATCACCATACTTCTTACAGATCGTAATCTCAACACAACTTTCTTTGACCCGGCGGGAGGAGGCGACCCAATTCTATACCAGCACTTA

>KU236888.1 *Okamejei kenojei*

TGAGCAGGGATGGTTGGAACTGGCCTAAGCCTCTTAATCCGAGCAGAACTGAGCCAACCGGGGGCCCTCCTAGGTGATGATCAGATTTATAATGTCATTGTTACAGCCCACGCCTTAGTAATAATCTTTTTTATGGTTATACCAATTATAATCGGCGGGTTCGGTAACTGACTTGTCCCCTTAATGATCGGCTCCCCAGACATAGCCTTCCCACGCATAAATAATATAAGTTTTTGGCTCCTGCCCCCATCTTTTCTTCTCCTCCTGGCCTCAGCCGGAGTCGAGGCCGGGGCCGGGACAGGTTGAACCGTCTACCCCCCACTAGCAGGGAATCTAGCCCACGCCGGGGCCTCCGTAGACCTGACAATTTTCTCCCTTCACTTGGCAGGCATCTCATCTATCCTAGCCTCCATTAACTTCATCACCACAATTATCAATATAAAACCACCAGCAATCTCCCAATACCAAACACCATTATTCGTATGATCAATTCTTGTTACAACTGTCTTACTTCTTATAGCCCTACCAGTTCTAGCAGCTGGCATCACCATACTTCTTACAGATCGTAATCTCAACACAACTTTCTTTGACCCGGCGGGAGGAGGCGACCCAATTCTATACCAGCACTTA

>KU236889.1 *Okamejei kenojei*

TGAGCAGGGATGGTTGGAACTGGCCTAAGCCTCTTAATCCGAGCAGAACTGAGCCAACCGGGGACCCTCCTAGGTGATGATCAGATTTATAATGTCATTGTTACAGCCCACGCCTTAGTAATAATCTTTTTTATGGTTATACCAATTATAATCGGCGGGTTCGGTAACTGACTTGTCCCCTTAATGATCGGCTCCCCAGACATAGCCTTCCCACGCATAAATAATATAAGTTTTTGGCTCCTGCCCCCATCTTTTCTTCTCCTCCTGGCCTCAGCCGGAGTCGAGGCCGGGGCCGGGACAGGTTGAACCGTCTACCCCCCACTAGCAGGGAATCTAGCCCACGCCGGGGCCTCCGTAGACCTGACAATTTTCTCCCTTCACTTGGCAGGCATCTCATCTATCCTAGCCTCCATTAACTTCATCACCACAATTATCAATATAAAACCACCAGCAATCTCCCAATACCAAACACCATTATTCGTATGATCAATTCTTGTTACAACTGTCTTACTTCTTATAGCCCTACCAGTTCTAGCAGCTGGCATCACCATACTTCTTACAGATCGTAATCTCAACACAACTTTCTTTGACCCGGCGGGAGGAGGCGACCCAATTCTATACCAGCACTTA

>KU236890.1 *Okamejei kenojei*

TGAGCAGGGATGGTTGGGACTGGCCTAAGCCTCTTAATCCGAGCAGAACTAAGCCAACCGGGGACCCTCCTAGGTGATGATCAGATTTATAATGTCATTGTTACAGCCCACGCCTTAGTAATAATCTTTTTTATGGTTATACCAATTATAATCGGCGGGTTCGGTAACTGACTTGTCCCCTTAATGATCGGCTCCCCAGACATAGCCTTCCCCCGCATAAATAATATAAGTTTTTGGCTCCTGCCCCCATCTTTTCTTCTCCTCCTGGCCTCAGCCGGAGTCGAGGCCGGAGCCGGGACAGGTTGAACCGTCTACCCCCCACTAGCAGGGAATCTAGCCCACGCCGGGGCCTCCGTAGACCTGACAATTTTCTCCCTTCACTTGGCAGGCATCTCATCTATCCTAGCCTCCATTAACTTCATCACCACAATTATCAATATAAAACCACCAGCAATCTCCCAATACCAAACACCATTATTCGTATGATCAATTCTTGTTACAACTGTCTTACTTCTTATAGCCCTACCAGTTCTAGCAGCTGGCATCACCATACTTCTTACAGATCGTAATCTCAACACAACTTTCTTTGACCCGGCGGGAGGAGGCGACCCAATTCTATACCAGCACTTA

>KU236891.1 *Okamejei kenojei*

TGAGCAGGGATGGTTGGAACTGGCCTAAGCCTCTTAATCCGAGCAGAACTGAGCCAACCGGGGACCCTCCTAGGTGATGATCAGATTTATAATGTCATTGTTACAGCCCACGCCTTAGTAATAATCTTTTTTATGGTTATACCAATTATAATCGGCGGGTTCGGTAACTGACTTGTCCCCTTAATGATCGGCTCCCCAGACATAGCCTTCCCACGCATAAATAATATAAGTTTTTGGCTCCTGCCCCCATCTTTTCTTCTCCTCCTGGCCTCAGCCGGAGTCGAGGCCGGGGCCGGGACAGGTTGAACCGTCTACCCCCCACTAGCAGGGAATCTAGCCCACGCCGGGGCCTCCGTAGACCTGACAATTTTCTCCCTTCACTTGGCAGGCATCTCATCTATCCTAGCCTCCATTAACTTCATCACCACAATTATCAATATAAAACCACCAGCAATCTCCCAATACCAAACACCATTATTCGTATGATCAATTCTTGTTACAACTGTCTTACTTCTTATAGCCCTACCAGTTCTAGCAGCTGGCATCACCATACTTCTTACAGATCGTAATCTCAACACAACTTTCTTTGACCCAGCGGGAGGAGGCGACCCAATTCTATACCAGCACTTA

>KU236892.1 *Okamejei kenojei*

TGAGCAGGGATGGTTGGAACTGGCCTAAGCCTCTTAATCCGAGCAGAACTGAGCCAACCGGGGACCCTCCTAGGTGATGATCAGATTTATAATGTCATTGTTACAGCCCACGCCTTAGTAATAATCTTTTTTATGGTTATACCAATTATAATCGGCGGGTTCGGTAACTGACTTGTCCCCTTAATGATCGGCTCCCCAGACATAGCCTTCCCACGCATAAATAATATAAGTTTTTGGCTCCTGCCCCCATCTTTTCTTCTCCTCCTGGCCTCAGCCGGAGTCGAGGCCGGGGCCGGGACAGGTTGAACCGTCTACCCCCCACTAGCAGGGAATCTAGCCCACGCCGGGGCCTCCGTAGACCTGACAATTTTCTCCCTTCACTTGGCAGGCATCTCATCTATCCTAGCCTCCATTAACTTCATCACCACAATTATCAATATAAAACCACCAGCAATCTCCCAATACCAAACACCATTATTCGTATGATCAATTCTTGTTACAACTGTCTTACTTCTTATAGCCCTACCAGTTCTAGCAGCTGGCATCACCATACTTCTTACAGATCGTAATCTCAACACAACTTTCTTTGACCCGGCGGGAGGAGGCGACCCAATTCTATACCAGCACTTA
