# Supplementary figures and images for: DNA barcoding of marine fish species from Rongcheng Bay, China
Source: PeerJ. 2018 Jun 25;6:e5013. doi: 10.7717/peerj.5013 (PMC6022726; doi:10.7717/peerj.5013)

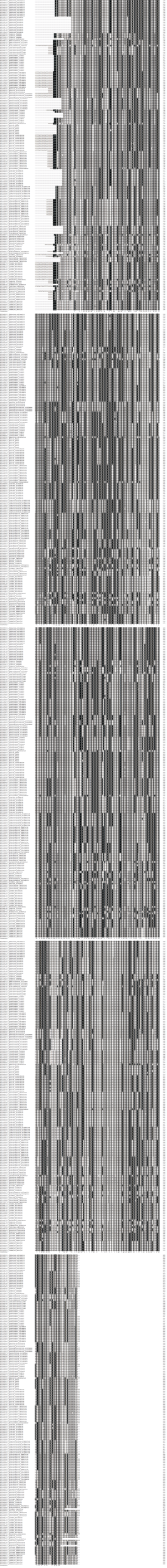

Supplement: File S2 [file peerj-06-5013-s002.png]
