# Supplementary material for: DNA barcoding of marine fish species from Rongcheng Bay, China
Source: PeerJ. 2018 Jun 25;6:e5013. doi: 10.7717/peerj.5013 (PMC6022726; doi:10.7717/peerj.5013)
Supplement: File S3 [file peerj-06-5013-s003.docx]

>*Cynoglossus joyneri*1

GTAGGAACTGCCCTAAGCCTACTCATTCGAGCAGAACTAAGCCAACCCGGCAGCCTACTTGGCGACGACCAAATCTATAATGTAATCGTTACCGCACATGCATTCGTAATGATTTTCTTTATAGTAATGCCTATTATGATTGGAGGCTTCGGAAATTGATTAATTCCACTAATAATCGGAGCCCCAGACATAGCATTTCCACGAATAAATAATATAAGCTTCTGACTTCTCCCTCCTTCTTTCCTCCTTCTTCTTGCTTCCTCTGCTGTAGAGGCCGGAGCTGGTACAGGTTGAACTGTTTACCCACCTCTTGCAGGCAACCTAGCCCATGCTGGTGCATCCGTAGATCTTACCATCTTCTCGCTCCATCTAGCAGGGGTGTCCTCAATTTTAGGGGCAATCAATTTTATTACCACAGTTCTTAATATAAAACCTGAAGGTATAACAATATACCAAGTACCTCTATTTGTATGAGCAGTACTTATTACAGCAGTTCTTTTACTTCTCTCCCTCCCTGTTTTAGCTGCTGGAATTACTATACTACTCACAGATCGAAATCTAAACACCACTTTCTTTGACCCCGCTGGAGGAGGGGATCCAATCCTCTACCAACACCTA

>*Cynoglossus joyneri*2

GTAGGAACTGCCCTAAGCCTACTCATTCGAGCAGAACTAAGCCAACCCGGCAGCCTACTTGGCGACGACCAAATCTATAATGTAATCGTTACCGCACATGCATTCGTAATGATTTTCTTTATAGTAATGCCTATTATGATTGGAGGCTTCGGAAATTGATTAATTCCACTAATAATCGGAGCCCCAGACATAGCATTTCCACGAATAAATAATATAAGCTTCTGACTTCTCCCTCCTTCTTTCCTCCTTCTTCTTGCTTCCTCTGCTGTAGAGGCCGGAGCTGGTACAGGTTGAACTGTTTACCCACCTCTTGCAGGCAACCTAGCCCATGCTGGTGCATCCGTAGATCTTACCATCTTCTCGCTCCATCTAGCAGGGGTATCCTCAATTTTAGGGGCAATCAATTTTATTACCACAGTTCTTAATATAAAACCTGAAGGTATAACAATATACCAAGTACCTCTATTTGTATGAGCAGTACTTATTACAGCAGTTCTTTTACTTCTCTCCCTCCCTGTTTTAGCTGCTGGAATTACTATACTACTCACAGATCGAAATCTAAACACCACTTTCTTTGACCCCGCTGGAGGAGGGGATCCAATCCTCTACCAACACCTA

>*Cynoglossus joyneri3*

GTAGGAACTGCCCTAAGCCTACTCATTCGAGCAGAACTAAGCCAACCCGGCAGCCTACTTGGCGACGACCAAATCTATAATGTAATCGTTACCGCACATGCATTCGTAATGATTTTCTTTATAGTAATGCCTATTATGATTGGAGGCTTCGGAAATTGACTAATTCCACTAATAATCGGAGCCCCAGACATAGCATTTCCACGAATAAATAATATAAGCTTCTGACTTCTCCCTCCTTCTTTCCTCCTTCTTCTTGCTTCCTCTGCTGTAGAGGCCGGAGCTGGTACAGGTTGAACTGTTTACCCACCTCTTGCAGGCAACCTAGCCCATGCTGGTGCATCCGTAGATCTTACCATCTTCTCGCTCCATCTAGCAGGGGTGTCCTCAATTTTAGGGGCAATCAATTTTATTACCACAGTTCTTAATATAAAACCTGAAGGTATAACAATATATCAAGTACCTCTATTTGTATGAGCAGTACTTATTACAGCAGTTCTTTTACTTCTCTCCCTCCCTGTCTTAGCTGCTGGAATTACTATACTACTCACAGATCGAAATCTAAACACCACTTTCTTTGACCCCGCTGGAGGAGGGGATCCAATCCTCTACCAACACCTA

>*Cynoglossus joyneri*4

GGCTGAGCCGGAATAGTAGGAACTGCCCTAAGCCTACTCATTCGAGCAGAACTAAGCCAACCCGGCAGCCTACTTGGCGACGACCAAATCTATAATGTAATCGTTACCGCACATGCATTCGTAATGATTTTCTTTATAGTAATGCCTATTATGATTGGAGGCTTCGGAAATTGATTAATTCCACTAATAATCGGAGCCCCAGACATAGCATTTCCACGAATAAATAATATAAGCTTCTGACTTCTCCCTCCTTCTTTCCTCCTTCTTCTTGCTTCCTCTGCTGTAGAGGCCGGAGCTGGTACAGGTTGAACTGTTTACCCACCTCTTGCAGGCAACCTAGCCCATGCTGGTGCATCCGTAGATCTTACCATCTTCTCGCTCCATCTAGCAGGGGTGTCCTCAATTTTAGGGGCAATCAATTTTATTACCACAGTTCTTAATATAAAACCTGAAGGTATAACAATATACCAAGTACCTCTATTTGTATGAGCAGTACTTATTACAGCAGTTCTTTTACTTCTCTCCCTCCCTGTTTTAGCTGCTGGAATTACTATACTACTCACAGATCGAAATCTAAACACCACTTTCTTTGACCCCGCTGGAGGAGGGGATCCAATCCTCTACCAACACCTATTC

>*Cynoglossus joyneri*5

AGCCGGAATAGTAGGAACTGCCCTAAGCCTACTCATTCGAGCAGAACTAAGCCAACCCGGCAGCCTACTTGGCGACGACCAAATCTATAATGTAATCGTTACCGCACATGCATTCGTAATGATTTTCTTTATAGTAATGCCTATTATGATTGGAGGCTTCGGAAATTGATTAATTCCACTAATAATCGGAGCCCCAGACATAGCATTTCCACGAATAAATAATATAAGCTTCTGACTTCTCCCTCCTTCTTTCCTCCTTCTTCTTGCTTCCTCTGCTGTAGAGGCCGGAGCTGGTACAGGTTGAACTGTTTACCCACCTCTTGCAGGCAACCTAGCCCATGCTGGTGCATCCGTAGATCTTACCATCTTCTCGCTCCATCTAGCAGGGGTATCCTCAATTTTAGGGGCAATCAATTTTATTACCACAGTTCTTAATATAAAACCTGAAGGTATAACAATATACCAAGTACCTCTATTTGTATGAGCAGTACTTATTACAGCAGTTCTTTTACTTCTCTCCCTCCCTGTTTTAGCTGCTGGAATTACTATACTACTCACAGATCGAAATCTAAACACCACTTTCTTTGACCCCGCTGGAGGAGGGGATCCAATCCTCTACCAACACCTATTC

>*Cynoglossus lighti*1

GAGCCGGAATAGTAGGAACTGCCCTAAGCCTACTCATTCGAGCAGAACTAAGCCAACCCGGCAGCCTACTTGGCGACGACCAAATCTATAATGTAATCGTTACCGCACATGCATTCGTAATGATTTTCTTTATAGTAATGCCTATTATGATTGGAGGCTTCGGAAATTGACTAATTCCACTAATAATCGGAGCCCCAGACATAGCATTTCCACGAATAAATAATATAAGCTTCTGACTTCTCCCTCCTTCTTTCCTCCTTCTTCTTGCTTCCTCTGCTGTAGAGGCCGGAGCTGGTACAGGTTGAACTGTTTACCCACCTCTTGCAGGCAACCTAGCCCATGCTGGTGCATCCGTAGATCTTACCATCTTCTCGCTCCATCTAGCAGGGGTGTCCTCAATTTTAGGGGCAATCAATTTTATTACCACAGTTCTTAATATAAAACCTGAAGGTATAACAATATATCAAGTACCTCTATTTGTATGAGCAGTACTTATTACAGCAGTTCTTTTACTTCTCTCCCTCCCTGTCTTAGCTGCTGGAATTACTATACTACTCACAGATCGAAATCTAAACACCACTTTCTTTGACCCCGCTGGAGGAGGGGATCCAATCCTCTACCAACACCTA

>*Cynoglossus lighti*2

GAGCCGGAATAGTAGGAACTGCCCTAAGCCTACTCATTCGAGCAGAACTAAGCCAACCCGGCAGCCTACTTGGCGACGACCAAATCTATAATGTAATCGTTACCGCACATGCATTCGTAATGATTTTCTTTATAGTAATGCCTATTATGATTGGAGGCTTCGGAAATTGATTAATTCCACTAATAATCGGAGCCCCAGACATAGCATTTCCACGAATAAATAATATAAGCTTCTGACTTCTCCCTCCTTCTTTCCTCCTTCTTCTTGCTTCCTCTGCTGTAGAGGCCGGAGCTGGTACAGGTTGAACTGTTTACCCACCTCTTGCAGGCAACCTAGCCCATGCTGGTGCATCCGTAGATCTTACCATCTTCTCGCTCCATCTAGCAGGGGTGTCCTCAATTTTAGGGGCAATCAATTTTATTACCACAGTTCTTAATATAAAACCTGAAGGTATAACAATATACCAAGTACCTCTATTTGTATGAGCAGTACTCATTACAGCAGTTCTTTTACTTCTCTCCCTCCCTGTTTTAGCTGCTGGAATTACTATACTACTCACAGATCGAAATCTAAACACCACTTTCTTTGACCCCGCTGGAGGAGGGGATCCAATCCTCTACCAACACCTA

>*Cynoglossus lighti*3

GAGCCGGAATAGTAGGAACTGCCCTAAGCCTACTCATTCGAGCAGAACTAAGCCAACCCGGCAGCCTACTTGGCGACGACCAAATCTATAATGTAATCGTTACCGCACATGCATTCGTAATGATTTTCTTTATAGTAATGCCTATTATGATTGGAGGCTTCGGAAATTGATTAATTCCACTAATAATCGGAGCCCCAGACATAGCATTTCCACGAATAAATAATATAAGCTTCTGACTTCTCCCTCCTTCTTTCCTCCTTCTTCTTGCTTCCTCTGCTGTAGAGGCCGGAGCTGGTACAGGTTGAACTGTTTACCCACCTCTTGCAGGCAACCTAGCCCATGCTGGTGCATCCGTAGATCTTACCATCTTCTCGCTCCATCTAGCAGGGGTGTCCTCAATTTTAGGGGCAATCAATTTTATTACCACAGTTCTTAATATAAAACCTGAAGGTATAACAATATACCAAGTACCTCTATTTGTATGAGCAGTACTTATTACAGCAGTTCTTTTACTTCTCTCCCTCCCTGTTCTAGCTGCTGGAATTACTATACTACTCACAGATCGAAATCTAAACACCACTTTCTTTGACCCCGCTGGAGGAGGGGATCCAATCCTCTACCAGCACCTA

>GU479053.1

GCCGGAATAGTAGGAACTGCCCTAAGCCTACTCATTCGAGCAGAACTAAGCCAACCCGGCAGCCTACTTGGCGACGACCAAATCTATAATGTAATCGTTACCGCACATGCATTCGTAATGATTTTCTTTATAGTAATGCCTATTATGATTGGAGGCTTCGGAAATTGATTAATTCCACTAATAATCGGAGCCCCAGACATAGCATTTCCACGAATAAATAATATAAGCTTCTGACTTCTCCCTCCTTCTTTCCTCCTTCTTCTTGCTTCCTCTGCTGTAGAGGCCGGAGCTGGTACAGGTTGAACTGTTTACCCACCTCTTGCAGGCAACCTAGCCCATGCTGGTGCATCCGTAGATCTTACCATCTTCTCGCTCCATCTAGCAGGGGTATCCTCAATTTTAGGGGCAATCAATTTTATTACCACAGTTCTTAATATAAAACCTGAAGGTATAACAATATACCAAGTACCTCTATTTGTATGAGCAGTACTTATTACAGCAGTTCTTTTACTTCTCTCCCTCCCTGTTTTAGCTGCTGGAATTACTATACTACTCACAGATCGAAATCTAAACACCACTTTCTTTGACCCCGCTGGAGGAGGGGATCCAATCCTCTACCAACACCTATTCTGATTCTTCGGTCACCCC

>KF979127.1

AGACATTGGCACCTTATATATAGTATTTGGGGCCTGAGCCGGAATAGTAGGAACTGCCCTAAGCCTACTCATTCGAGCAGAACTAAGCCAACCCGGCAGCCTACTTGGCGACGACCAAATCTATAATGTAATCGTTACCGCACATGCATTCGTAATGATTTTCTTTATAGTAATGCCTATTATGATTGGAGGCTTCGGAAATTGATTAATTCCACTAATAATTGGAGCCCCAGACATAGCATTTCCACGAATAAATAATATAAGCTTCTGACTTCTCCCTCCTTCTTTCCTCCTCCTTCTTGCTTCCTCTGCTGTAGAGGCCGGAGCTGGTACAGGTTGAACTGTTTACCCACCTCTTGCAGGCAACCTAGCCCATGCTGGTGCATCCGTAGATCTTACCATCTTCTCGCTCCATCTAGCAGGGGTGTCCTCAATTTTAGGGGCAATCAATTTTATTACCACAGTTCTTAATATAAAACCTGAAGGTATAACAATATACCAAGTACCTCTATTTGTATGAGCAGTACTTATTACAGCAGTTCTTTTACTTCTCTCCCTCCCTGTTTTAGCTGCTGGAATTACTATACTACTCACAGATCGAAATCTAAACACCACTTTCTTTGACCCCGCTGGAGGAGGGGATCCAATCCTCTACCAACACCTATTCTGATTCTTCGG

>JQ738602.1

AGGAACTGCCCTAAGCCTACTCATTCGAGCAGAACTAAGCCAACCCGGCAGCCTACTTGGCGACGACCAAATCTATAATGTAATCGTTACCGCACATGCATTCGTAATGATTTTCTTTATAGTAATGCCTATTATGATTGGAGGCTTCGGAAATTGATTAATTCCACTAATAATCGGTGCCCCAGACATAGCATTTCCACGAATAAATAATATAAGCTTCTGACTTCTCCCTCCTTCTTTCCTCCTTCTTCTTGCTTCCTCTGCTGTAGAGGCCGGAGCTGGTACAGGTTGAACTGTTTACCCACCTCTTGCAGGCAACCTAGCCCATGCTGGTGCATCCGTAGATCTTACCATCTTCTCGCTCCATCTAGCAGGGGTGTCCTCAATTTTAGGGGCAATCAATTTTATTACCACAGTTCTTAATATAAAACCTGAAGGTATAACAATATACCAAGTACCTCTATTTGTATGAGCAGTACTTATTACAGCAGTTCTTTTACTTCTCTCCCTCCCTGTTCTAGCTGCTGGAATTACTATACTACTCACAGATCGAAATCTAAACACCACTTTCTTTGACCCCGCTGGAGGAGGAGATCCAATCCTCTACCAGCACCTA

>JQ738613.1

TAGTAGGAACTGCCCTAAGCCTACTCATTCGAGCAGAACTAAGCCAACCCGGCAGCCTACTTGGCGACGACCAAATCTATAATGTAATCGTTACCGCACATGCATTCGTAATGATTTTCTTTATAGTAATGCCTATTATGATTGGAGGCTTCGGAAATTGATTAATTCCACTAATAATCGGAGCCCCAGACATAGCATTCCCACGAATAAATAATATAAGCTTCTGACTTCTCCCTCCTTCTTTCCTCCTTCTTCTTGCTTCCTCTGCTGTAGAGGCCGGAGCTGGTACAGGTTGAACTGTTTACCCACCTCTTGCAGGCAACCTAGCCCATGCTGGTGCATCCGTAGATCTTACCATCTTCTCGCTTCATCTAGCAGGGGTGTCCTCAATTTTAGGGGCAATCAATTTTATTACCACAGTTCTTAATATAAAACCTGAAGGTATAACAATATACCAAGTACCTCTATTTGTATGAGCAGTACTTATTACAGCAGTTCTTTTACTTCTCTCCCTCCCTGTTTTAGCTGCTGGAATTACTATACTACTCACAGATCGAAATCTAAACACCACTTTCTTTGACCCCGCTGGAGGAGGGGATGCAATCCTCTACCAACACCTA

>DQ116752.1

GGAACTGCCCTAAGCCTACTCATTCGAGCAGAACTAAGCCAACCCGGCAGCCTACTTGGCGACGACCAAATCTATAATGTAATCGTTACCGCACATGCATTCGTAATGATTTTCTTTATAGTAATGCCTATTATGATTGGAGGCTTCGGAAATTGACTAATTCCACTAATAATCGGAGCCCCAGACATAGCATTTCCACGAATAAATAATATAAGCTTCTGACTTCTCCCTCCTTCTTTCCTCCTTCTTCTTGCTTCCTCTGCTGTAGAGGCCGGAGCTGGTACAGGTTGAACTGTTTACCCACCTCTTGCAGGCAACCTAGCCCATGCTGGTGCATCCGTAGATCTTACCATCTTCTCGCTCCATCTAGCAGGGGTGTCCTCAATTTTAGGGGCAATCAATTTTATTACCACAGTTCTTAATATAAAACCTGAGGGTATAACAATATACCAAGTACCTCTATTTGTATGAGCAGTACTTATTACAGCAGTTCTTTTACTTCTCTCCCTCCCTGTTTTAGCTGCTGGAATTACTATACTACTCACAGATCGAAATCTAAACACCACTTTCTTTGACCCCGCTGGAGGAGGGGATCCAATCCTCTACCAACACCTATTCTGATTTTTTGGTCAC

>HQ711865.1

GCCCTAAGCCTACTCATTCGAGCAGAACTAAGCCAACCCGGCAGCCTACTTGGCGACGACCAAATCTATAATGTAATCGTTACCGCACATGCATTCGTAATGATTTTCTTTATAGTAATGCCTATTATGATTGGAGGCTTCGGAAATTGATTAATTCCACTAATAATCGGAGCCCCAGACATAGCATTTCCACGAATAAATAATATAAGCTTCTGACTTCTCCCTCCTTCTTTCCTCCTTCTTCTTGCTTCCTCTGCTGTAGAGGCCGGAGCTGGTACAGGTTGAACTGTTTACCCACCTCTTGCAGGCAACCTAGCCCATGCTGGTGCATCCGTAGATCTTACCATCTTCTCGCTCCATCTAGCAGGGGTATCCTCAATTTTAGGGGCAATCAATTTTATTACCACAGTTCTTAATATAAAACCTGAAGGTATAACAATATACCAAGTACCTCTATTTGTATGAGCAGTACTTATTACAGCAGTTCTTTTACTTCTCTCCCTCCCTGTTTTAGCTGCTGGAATTACTATACTACTCACAGATCGAAATCTAAACACCACTTTCTTTGACCCCGCTGGAGGAGGGGATCCAATCCTCTACCAACACCTATTCTGATTCTTCGGTCACCC

>JQ738430.1

CTTATATATAGTATTTGGGGCCTGAGCCGGAATAGTAGGGACTGCCCTAAGCCTACTCATTCGAGCAGAACTAAGCCAACCCGGCAGCCTACTTGGCGACGACCAAATCTATAATGTAATCGTTACCGCACATGCATTCGTAATGATTTTCTTTATAGTAATGCCTATTATGATTGGAGGCTTCGGAAATTGATTAATTCCACTAATAATCGGAGCCCCAGACATAGCATTTCCACGAATAAATAATATAAGCTTCTGACTTCTCCCTCCTTCTTTCCTCCTTCTTCTTGCTTCCTCTGCTGTAGAGGCCGGAGCTGGTACAGGTTGAACTGTTTACCCACCTCTTGCAGGCAACCTAGCCCATGCTGGTGCATCCGTAGATCTTACCATCTTCTCGCTCCATCTAGCAGGGGTGTCCTCAATTTTAGGGGCAATCAATTTTATTACCACAGTTCTTAATATAAAACCTGAAGGTATAACAATATACCAAGTACCTCTATTTGTATGAGCAGTACTTATTACAGCAGTTCTTTTACTTCTCTCCCTCCCTGTTTTAGCTGCTGGARTTACTATACTACTCACAGATCGAAATCTAAACACCACTTTCTTTGACCCCGCTGGAGGGAG

>JQ738456.1

CTTATATATAGTATTTGGGGCCTGAGCCGGAATAGTAGGAACTGCCCTAAGCCTACTCATTCGAGCAGAACTAAGCCAACCCGGCAGCCTACTTGGCGACGACCAAATCTATAATGTAATCGTTACCGCACATGCATTCGTAATGATTTTCTTTATAGTAATGCCTATTATGATTGGAGGCTTCGGAAATTGATTAATTCCACTAATAATCGGAGCCCCAGACATAGCATTTCCACGAATAAATAATATAAGCTTCTGACTTCTCCCTCCTTCTTTCCTCCTTCTTCTTGCTTCCTCTGCTGTAGAGGCCGGAGCTGGTACAGGTTGAACTGTTTACCCACCTCTTGCAGGCAACCTAGCCCATGCTGGTGCATCCGTAGATCTTACCATCTTCTCGCTCCATCTAGCAGGGGTGTCCTCAATTTTAGGGGCAATCAATTTTATTACCACAGTTCTTAATATAAAACCTGAAGGTATAACAATATACCAAGTACCTCTATTTGTATGAGCAGTACTTATTACAGCAGTTCTTTTACTTCTCTCCCTCCCTGTTTTAGCTGCTGGAATTACTATACTACTTACAGATCGAAATCTAAACACCACTTTCTTTGACCCCGCTCGAGGTAATCATCCAGCATCTGCACCCACTCGGG

>JQ738468.1

CTTATATATAGTATTTGGGGCCTGAGCCGGAATAGTAGGAACTGCCCTAAGCCTACTCATTCGAGCAGAACTAAGCCAACCCGGCAGCCTACTTGGCGACGACCAGATCTATAATGTAATCGTTACCGCACATGCATTCGTAATGATTTTCTTTATAGTAACGCCTATTATGATCGGAGGCTTCGGAAATTGATTAATTCCACTAATAATCGGAGCCCCAGACATAGCATTTCCACGAATAAATAATATAAGCTTCTGACTTCTCCCTCCTTCTTTCCTCCTTCTTCTTGCTTCCTCTGCTGTAGAGGCCGGAGCTGGTACAGGTTGAACTGTTTACCCACCTCTTGCAGGCAACCTAGCCCATGCTGGTGCATCCGTAGATCTAACCATCTTCTCGCTCCATCTAGCAGGGGTGTCCTCAATTTTAGGGGCAATCAATTTTATTACCACAGTTCTTAATATAAAACCTGAAGGTATAACAATATACCAAGTACCTCTATTTGTATGAGCAGTACTTATTACAGCAGTTCTTTTACTTCTCTCCCTCCCTGTTTTAGCTGCTGGAATTACTATACTACTCACAGATCGAAATCTAAACACCACTTTCTTTGACCCCGTCTC
